# Supplementary material for: Exploiting the Nucleophilicity of the Nitrogen Atom of Imidazoles: One-Pot Three-Component Synthesis of Imidazo-Pyrazines
Source: Molecules. 2019 May 21;24(10):1959. doi: 10.3390/molecules24101959 (PMC6572241; doi:10.3390/molecules24101959)

SUPPORTING INFORMATION

Exploiting the Nucleophilicity of the Nitrogen Atom of Imidazoles: One-pot Three-component Synthesis of Imidazopyperazines

Ubaldina Galli,[a] Rejdia Hysenlika,[a] Fiorella Meneghetti,[b] Erika Del Grosso,[a] Sveva Pelliccia,[c] Ettore Novellino,[c] Mariateresa Giustiniano,[c*] and Gian Cesare Tron[a*]

a) Dipartimento di Scienze del Farmaco, Università degli Studi del Piemonte Orientale "A. Avogadro", Largo Donegani 2, 28100 Novara, Italy;

b) Dipartimento di Scienze Farmaceutiche, Università degli Studi di Milano, Via L. Mangiagalli 25, 20133 Milano, Italy;

c) Dipartimento di Farmacia, Università degli Studi di Napoli "Federico II", via D. Montesano 49, 80131 Napoli, Italy.

Contents:

- Copies of ^{1}H and ^{13}C spectra…………………………………………………...………....S3

^{1}H NMR
300 MHz, CD_3OD

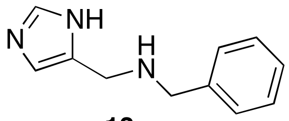

13

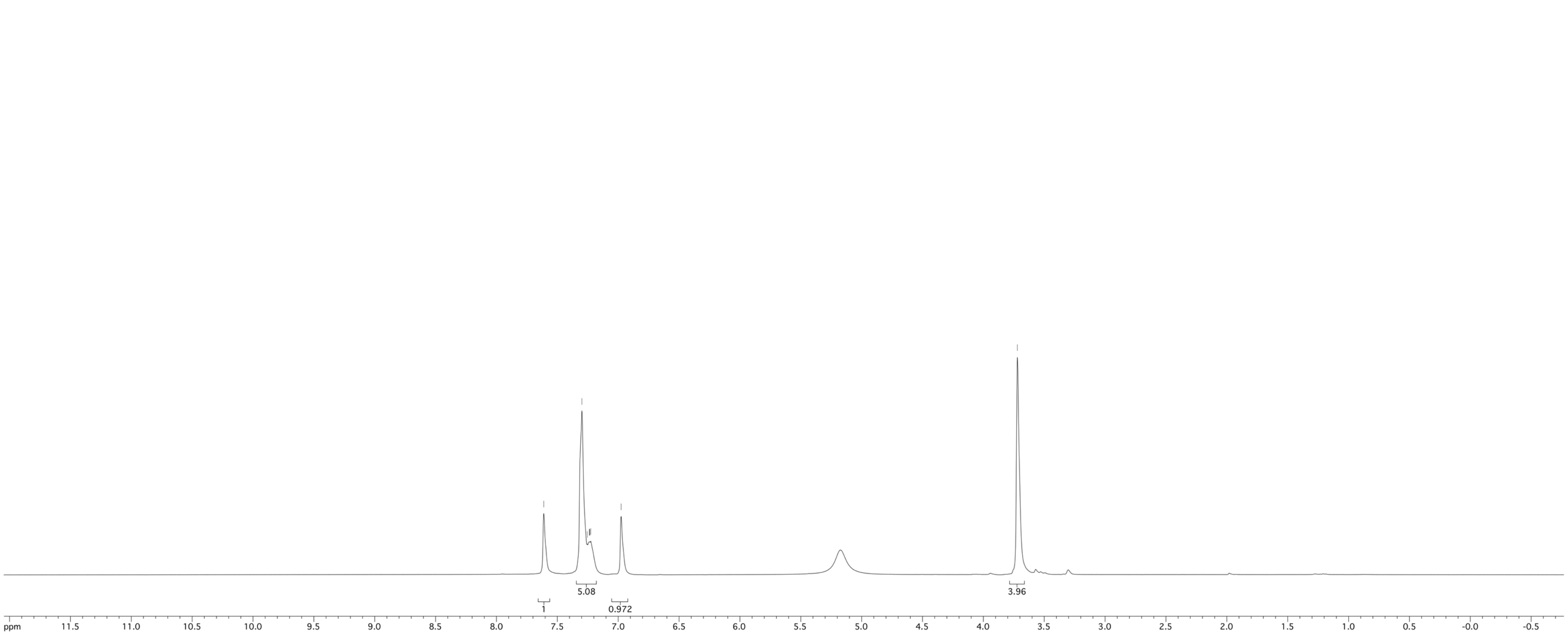

^{13}C NMR
75 MHz, CD_3OD

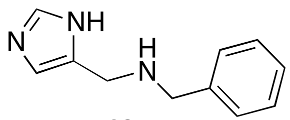

13

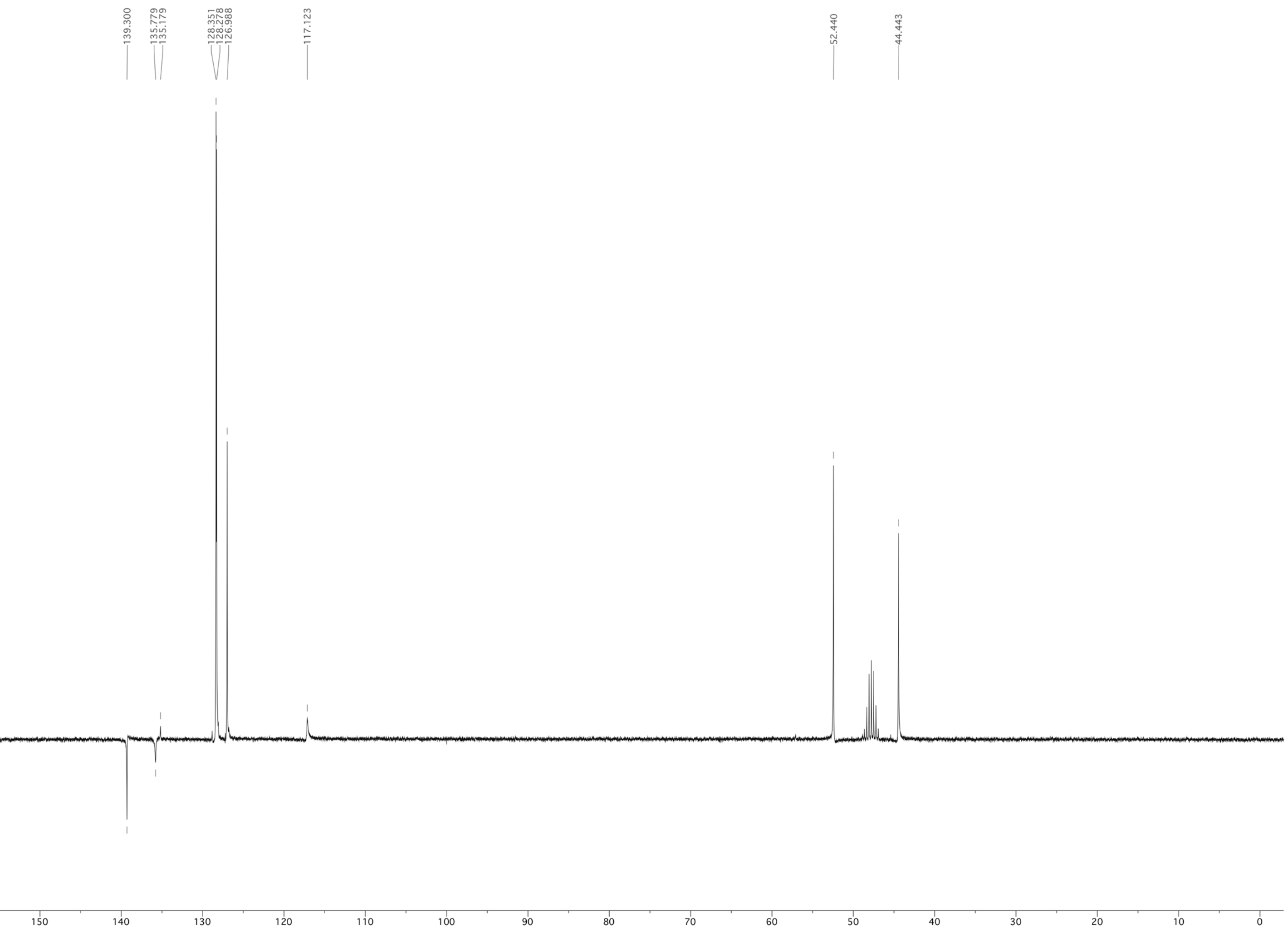

^{1}H NMR
300 MHz, CD_3OD

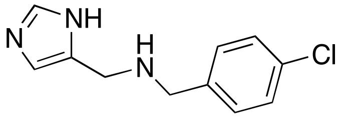

14

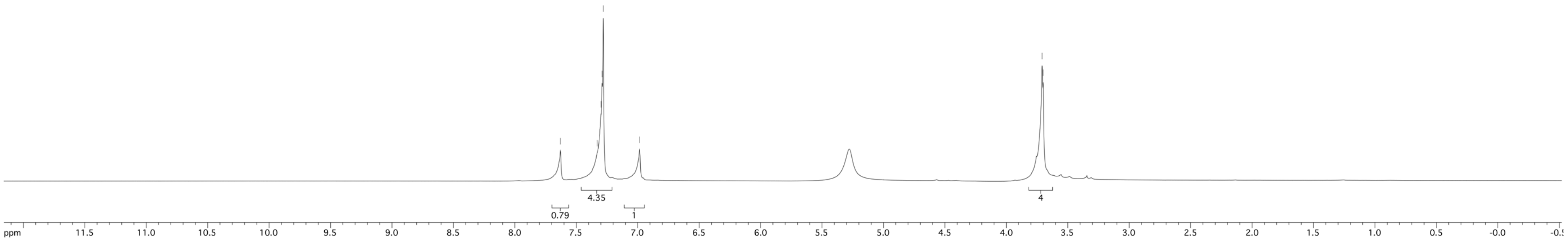

^{13}C NMR
75 MHz, CD_3OD

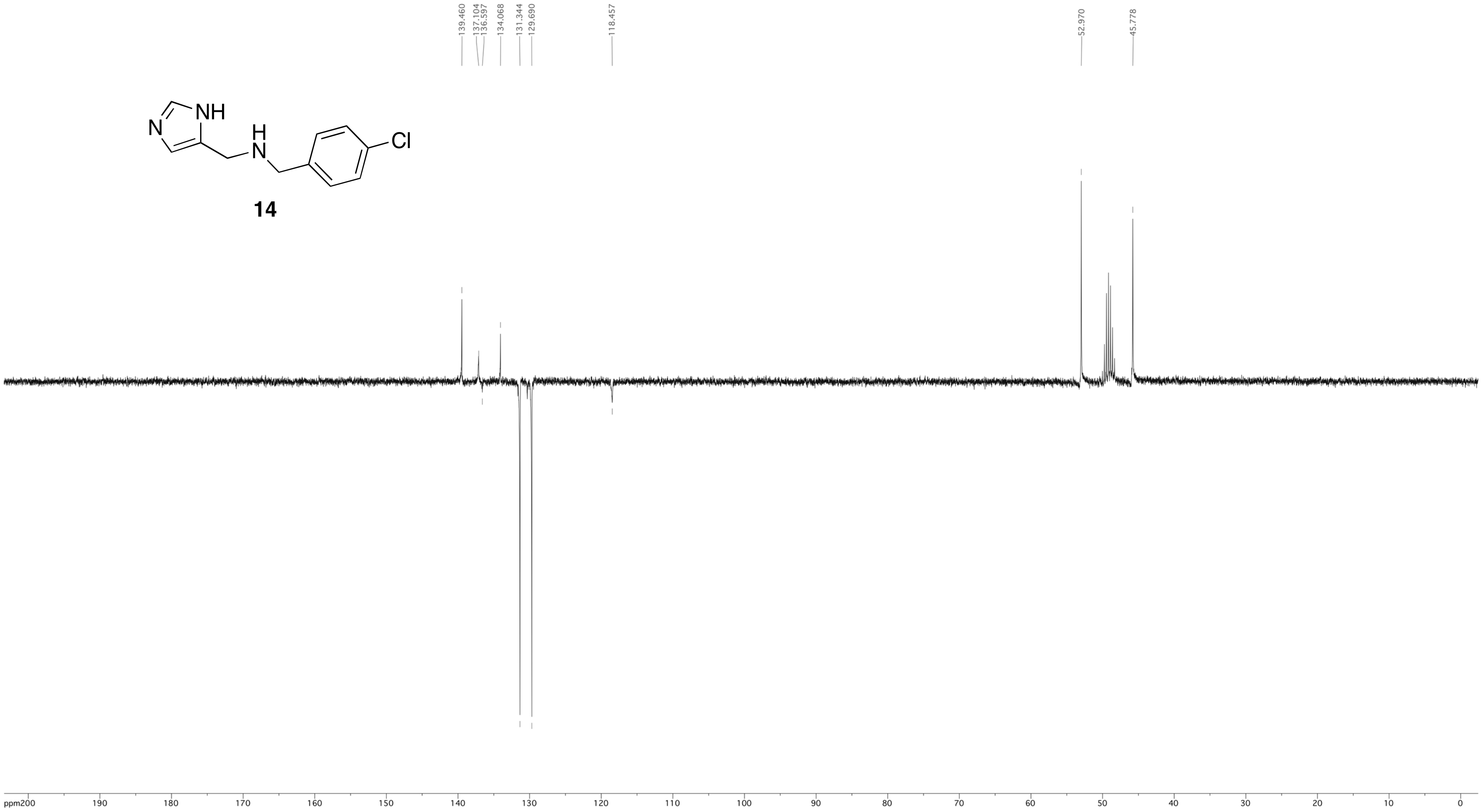

14

^{1}H NMR
400 MHz, CD_3OD

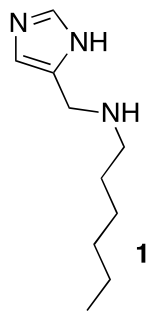

15

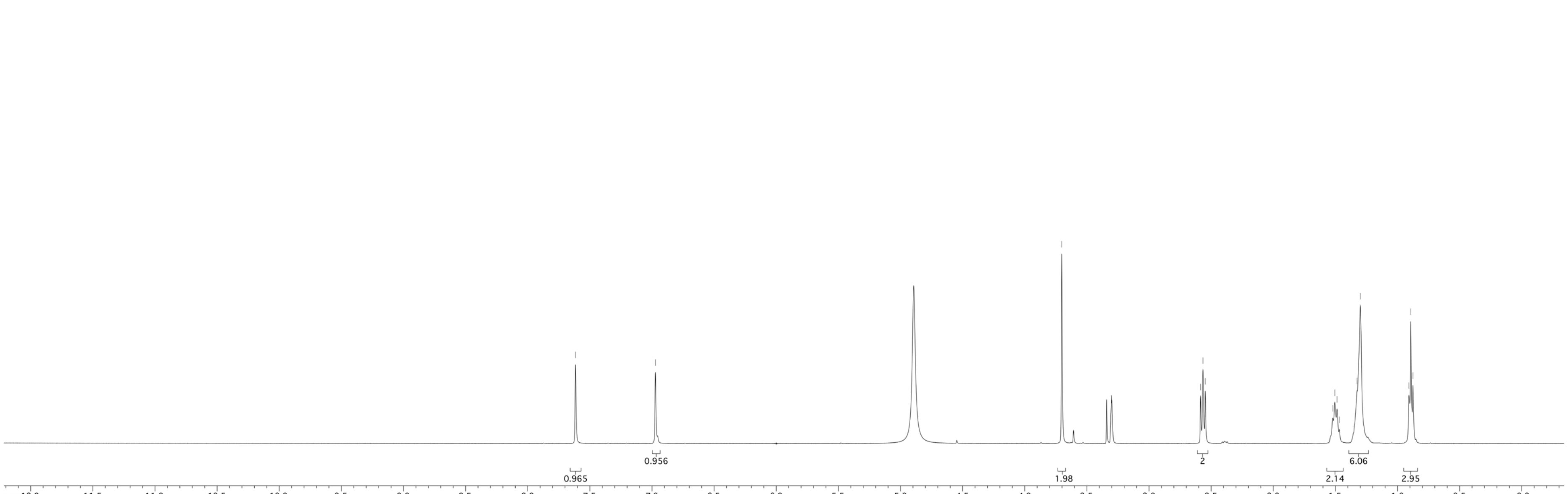

^{13}C NMR
100 MHz, CD_3OD

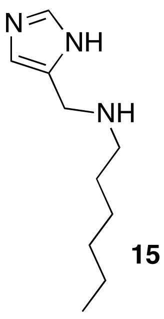

15

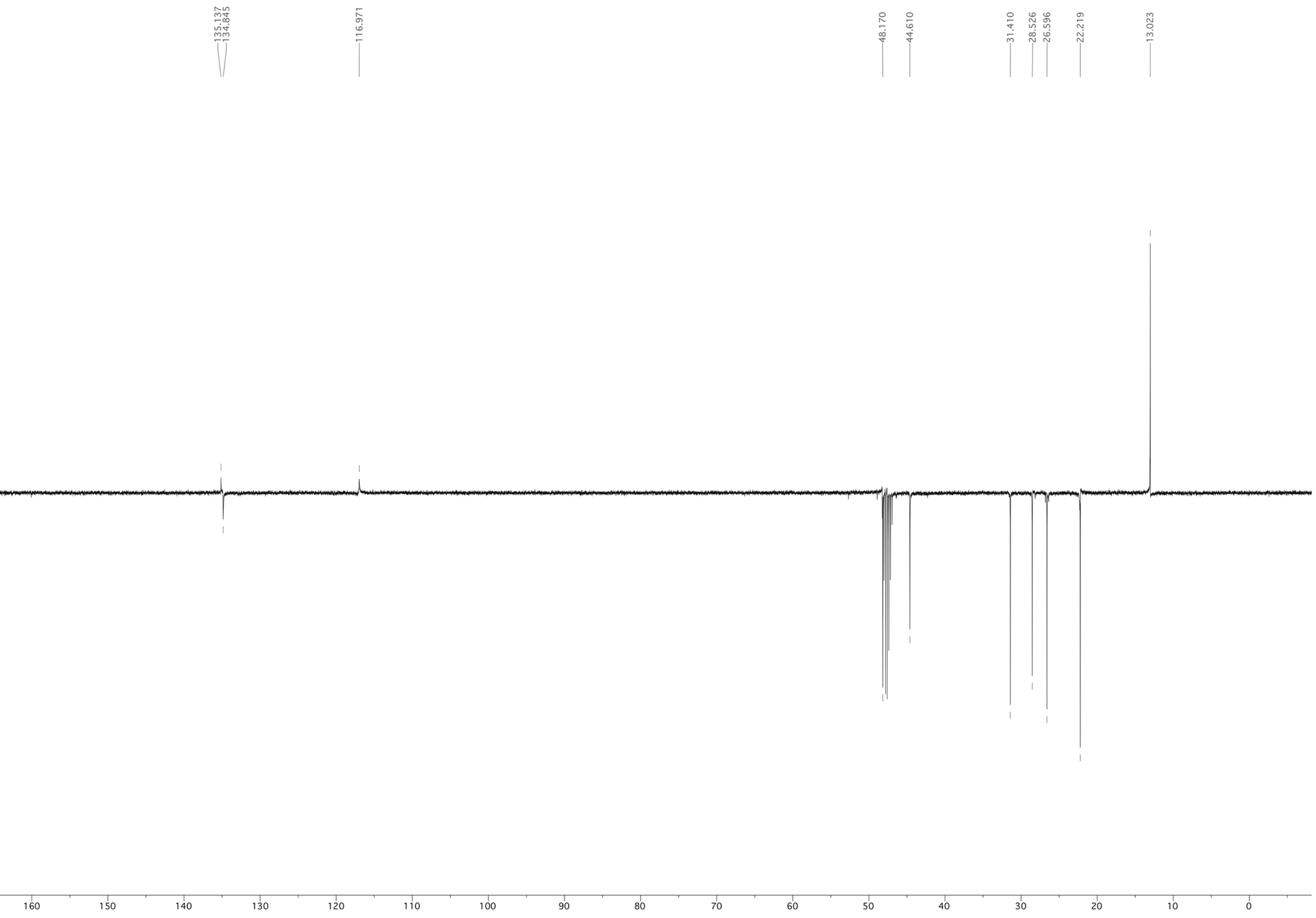

^1H NMR
400 MHz, CD_3OD

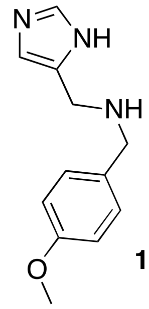

16

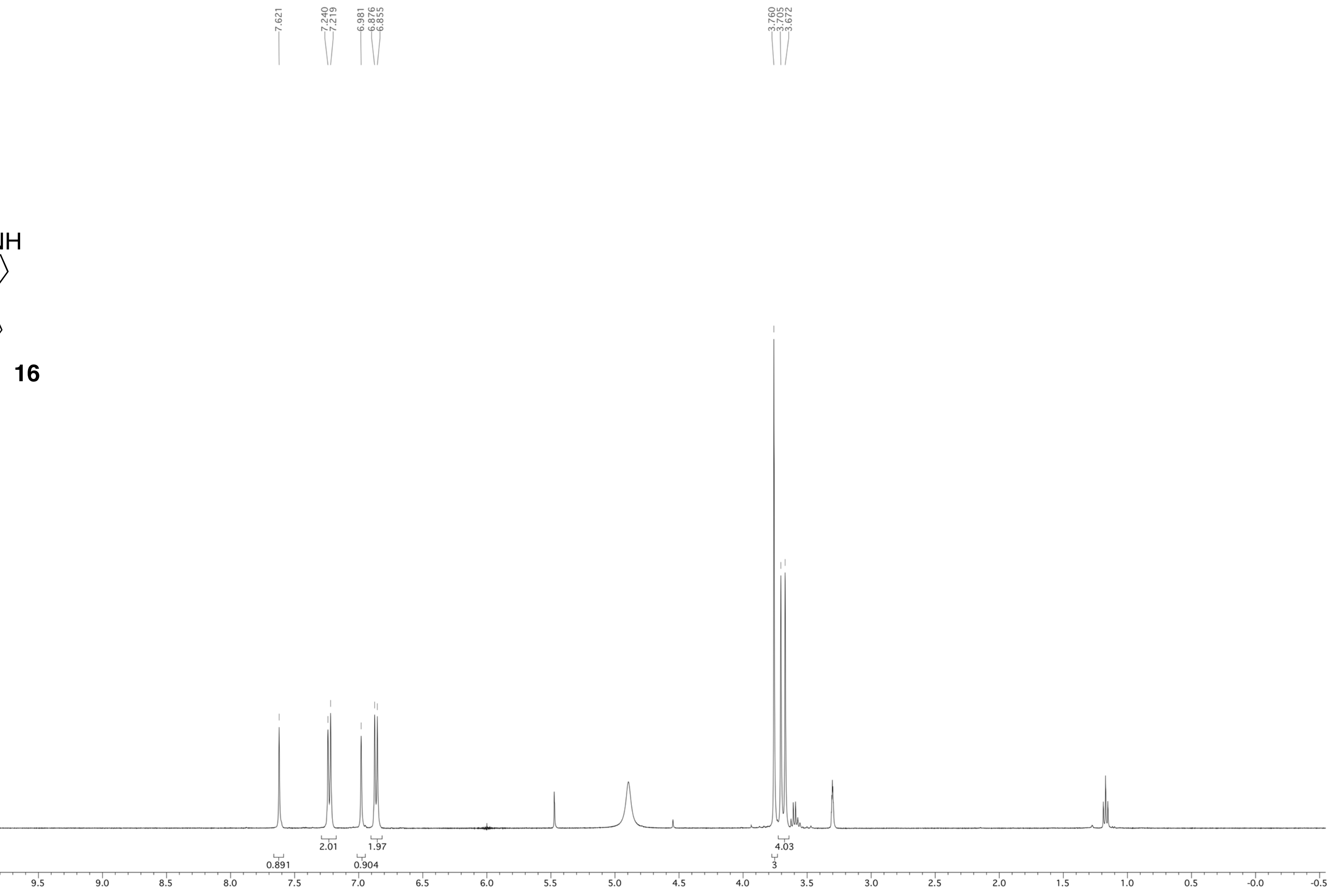

^{13}C NMR
100 MHz, CD_3OD

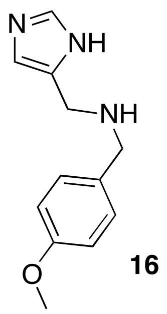

16

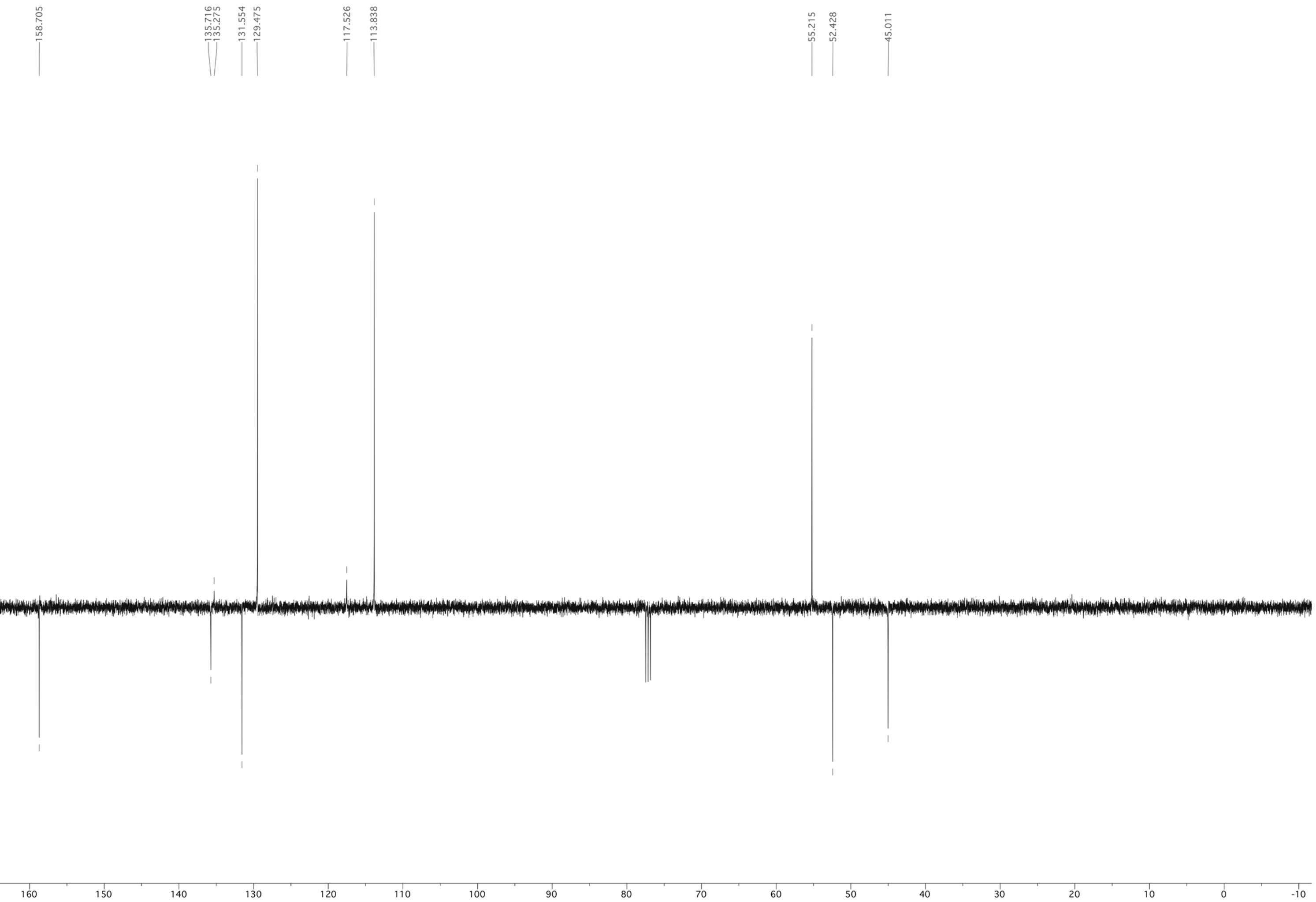

^1H NMR
400 MHz, CD_3OD

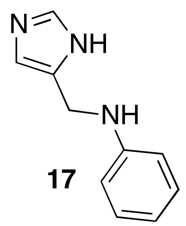

17

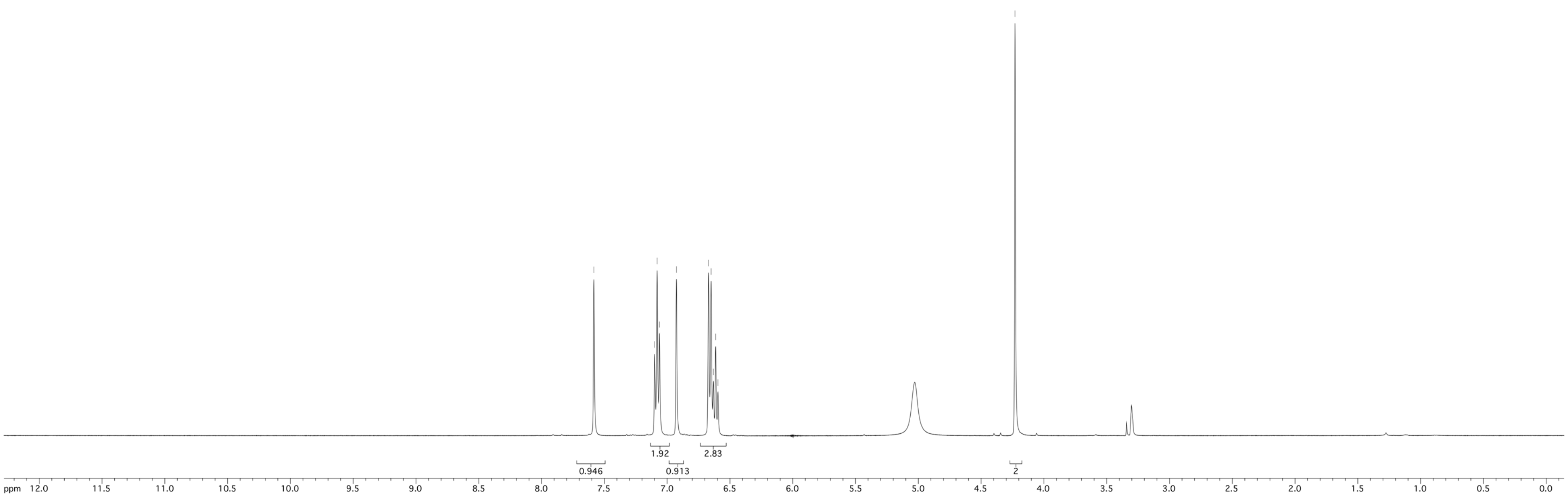

^{13}C NMR
100 MHz, CD_3OD

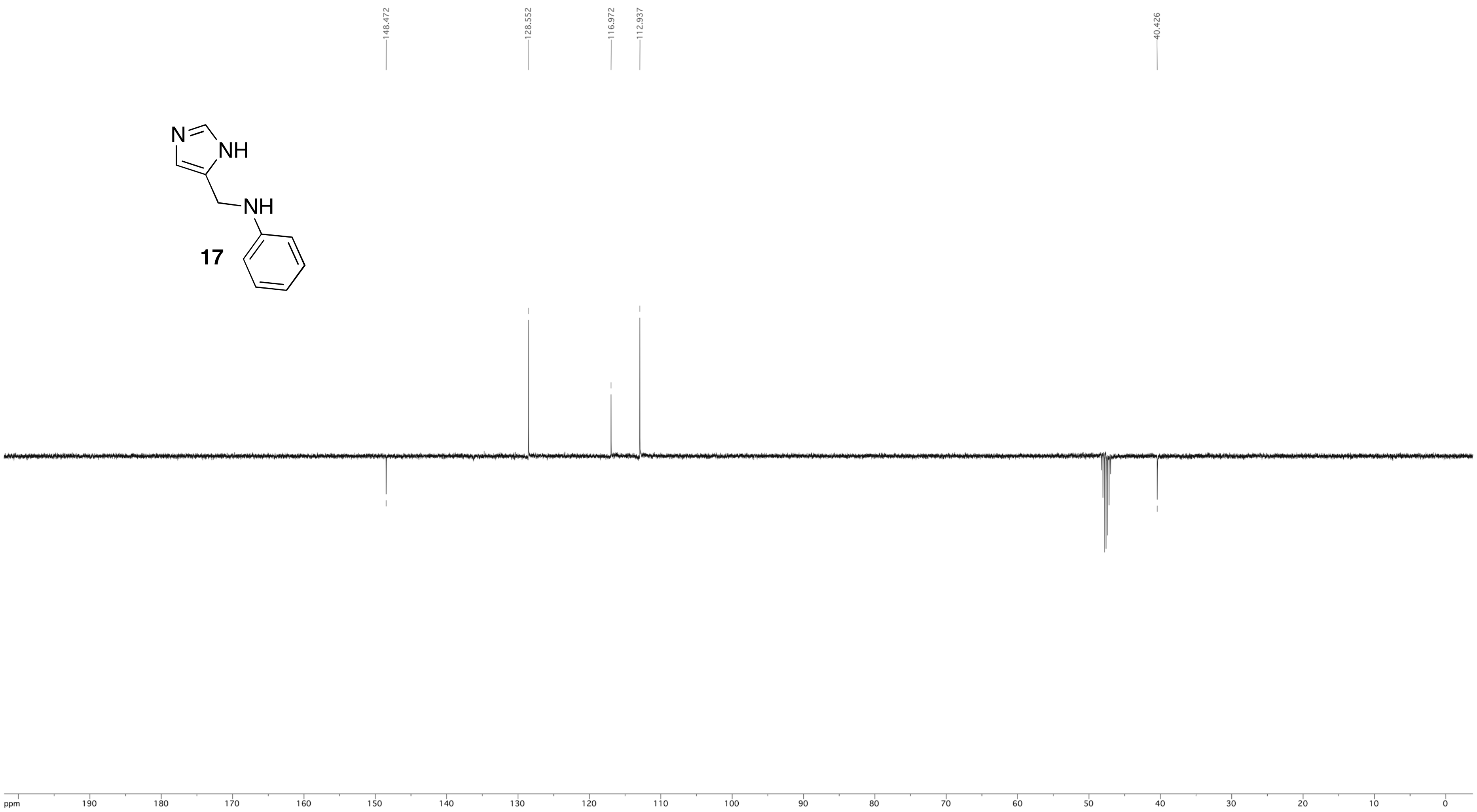

^{1}H NMR
300 MHz, CD_3OD

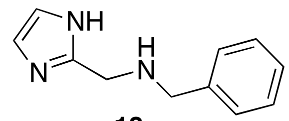

18

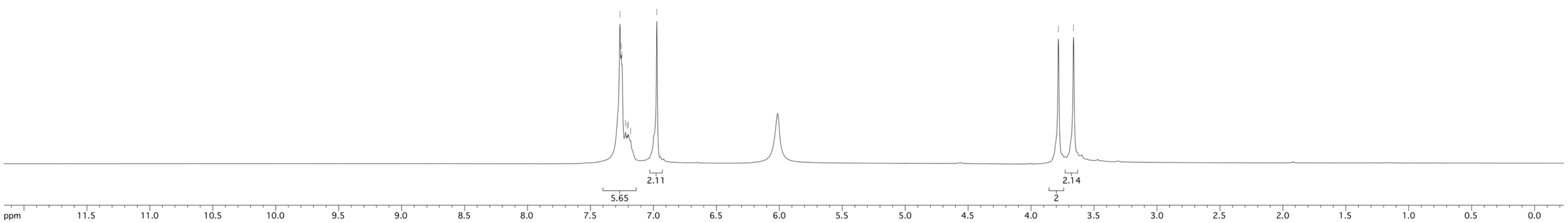

^{13}C NMR
75 MHz, CD_3OD

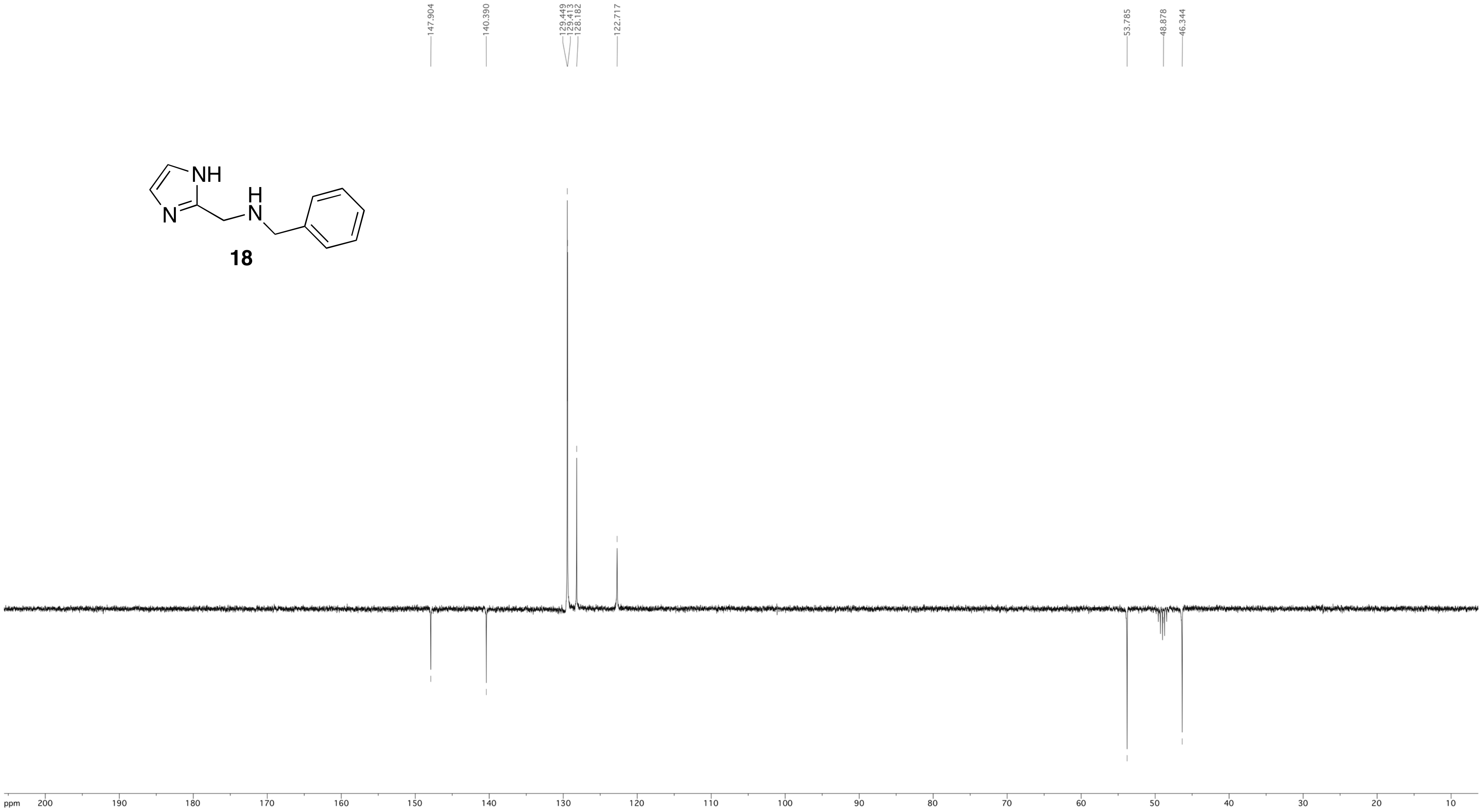

18

^{1}H NMR
300 MHz, CD_3OD

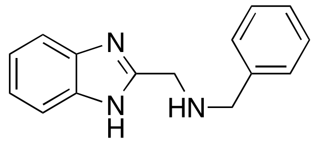

19

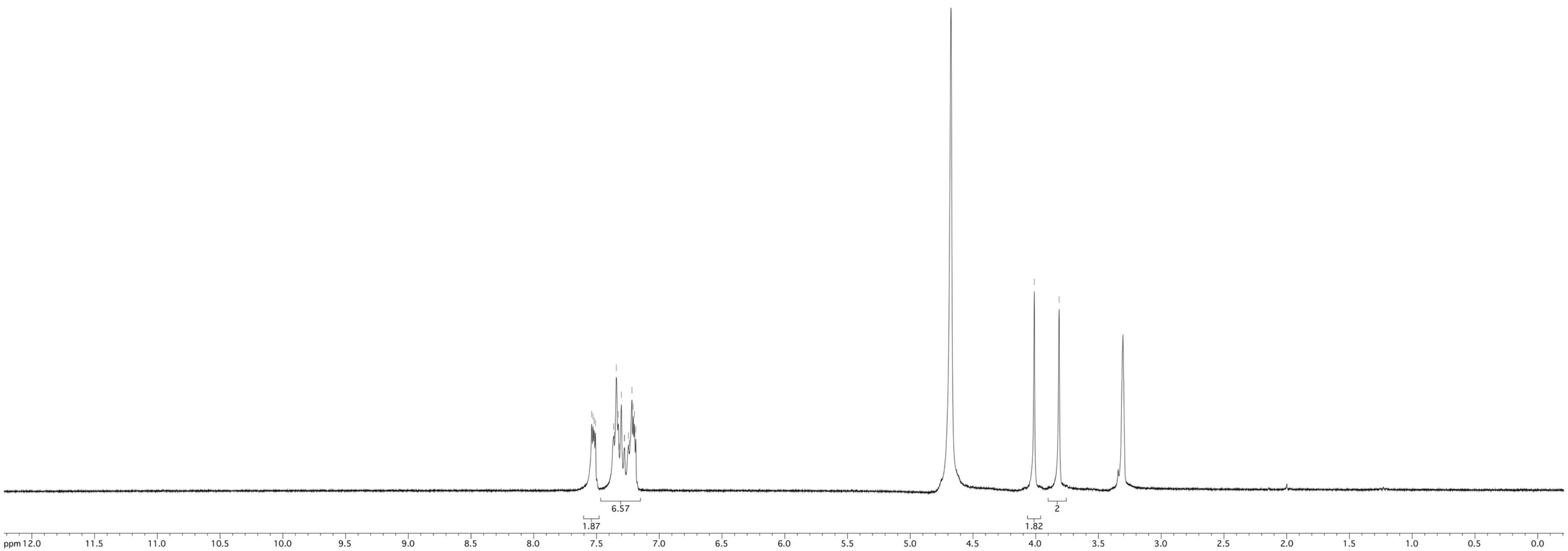

^{13}C NMR
75 MHz, CD_3OD

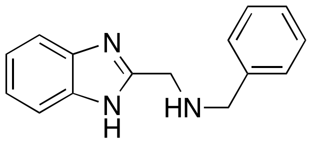

19

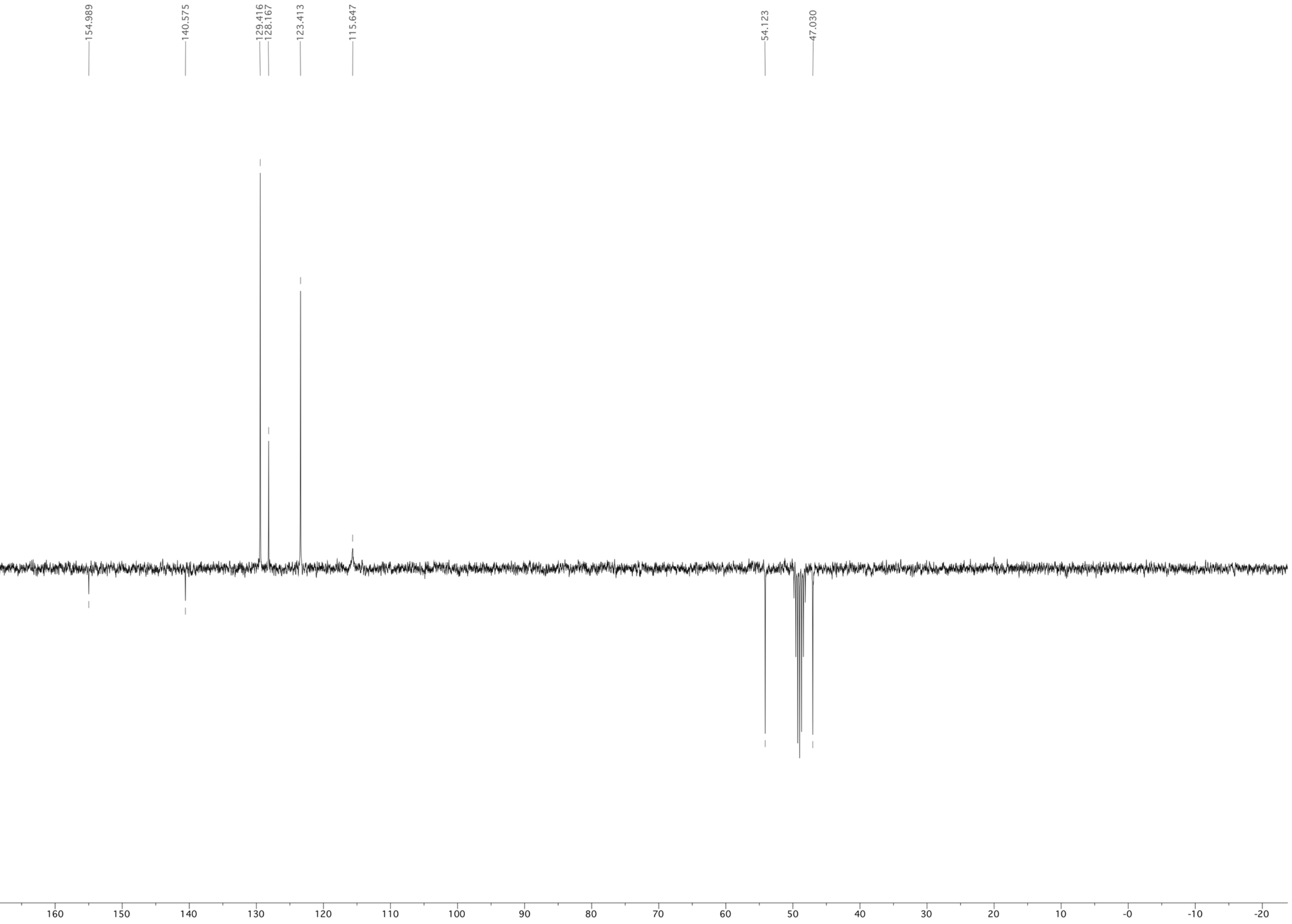

^{1}H NMR
300 MHz, CD_3OD

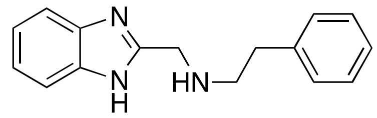

20

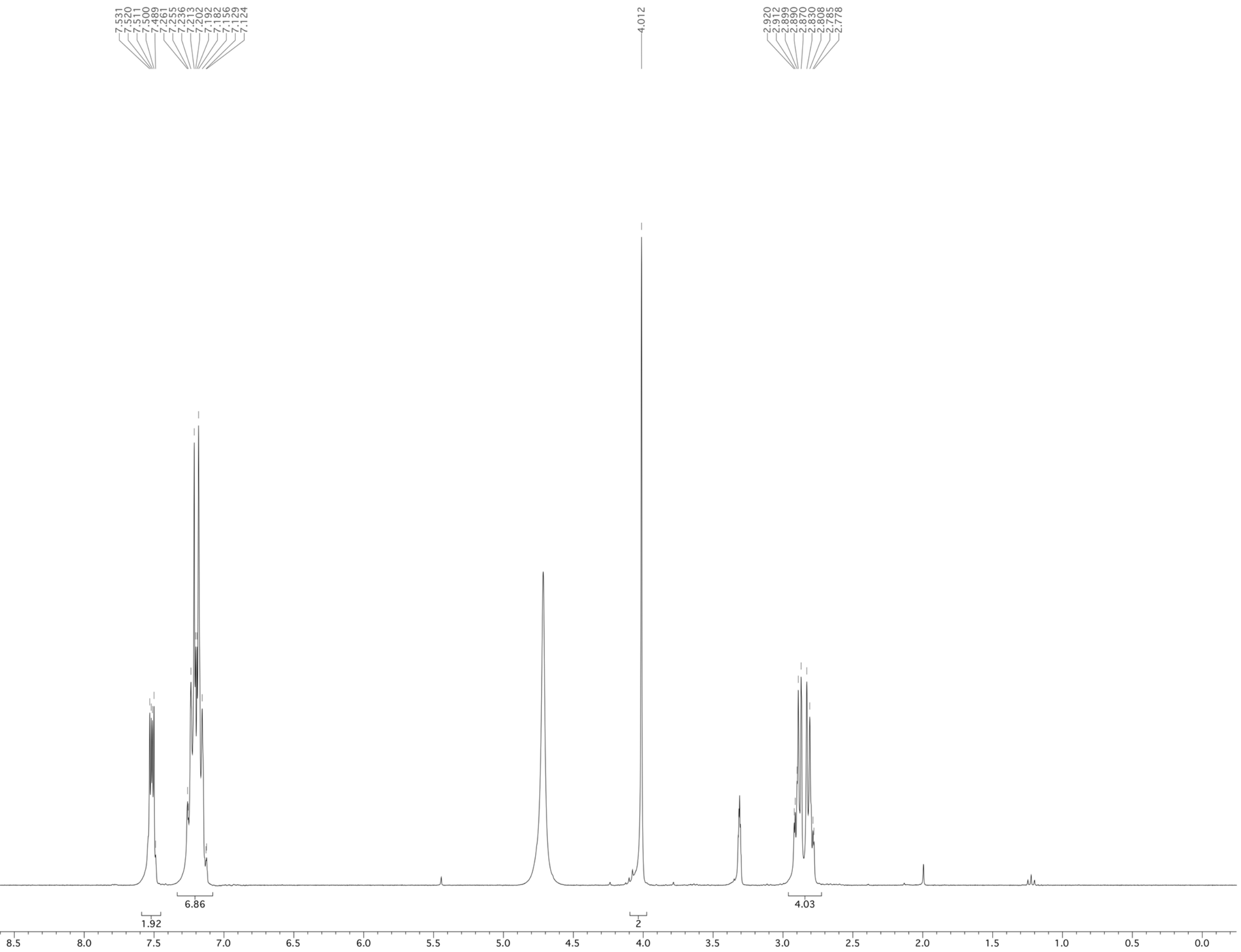

^{13}C NMR
75 MHz, CD_3OD

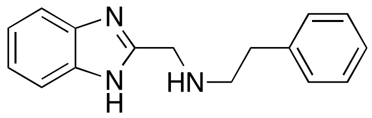

20

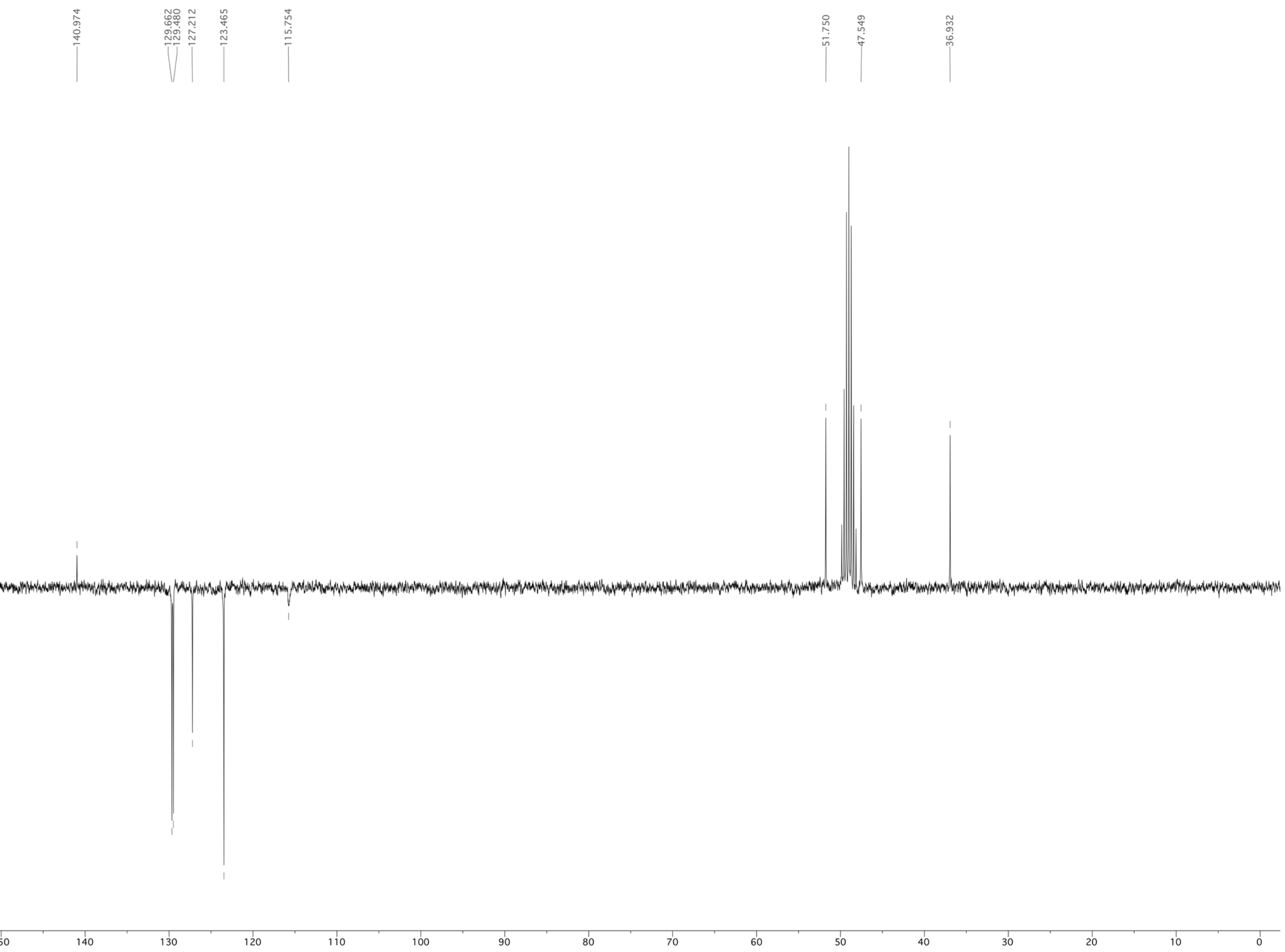

^{1}H NMR
300 MHz, $CDCl_3$

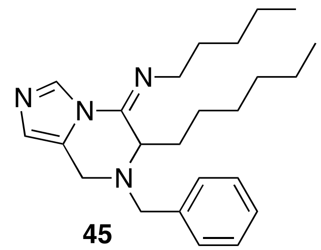

45

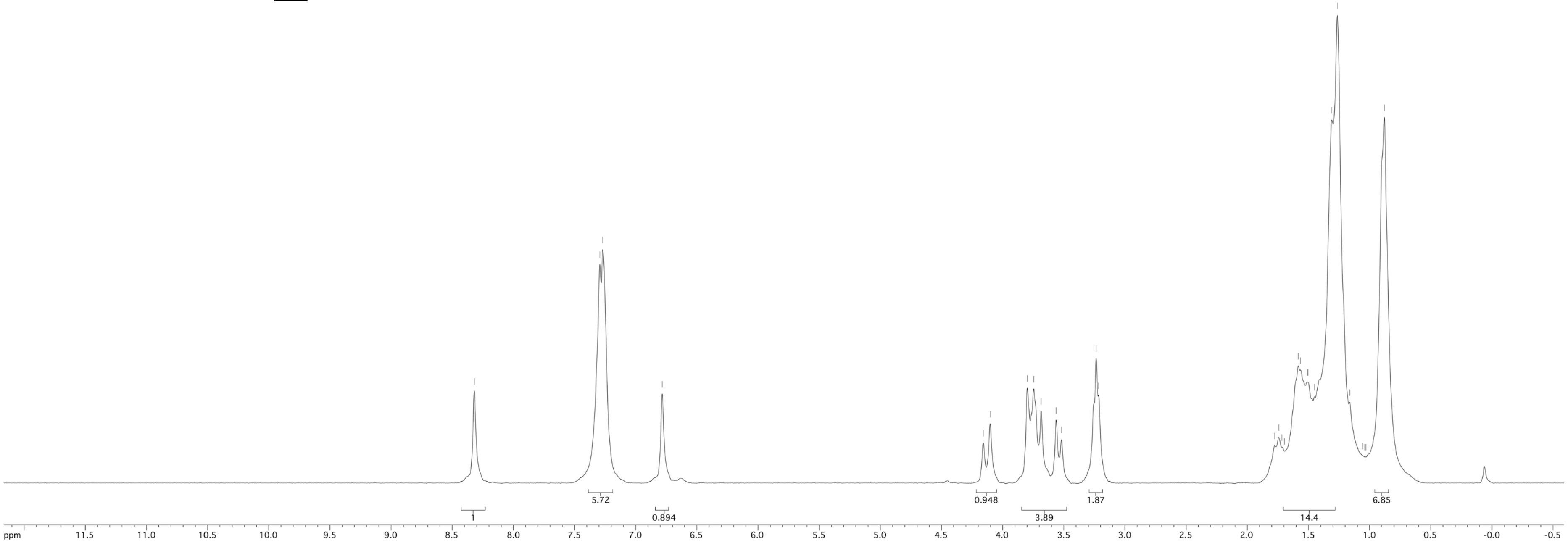

^{13}C NMR
75 MHz, $CDCl_3$

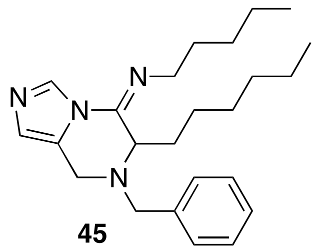

45

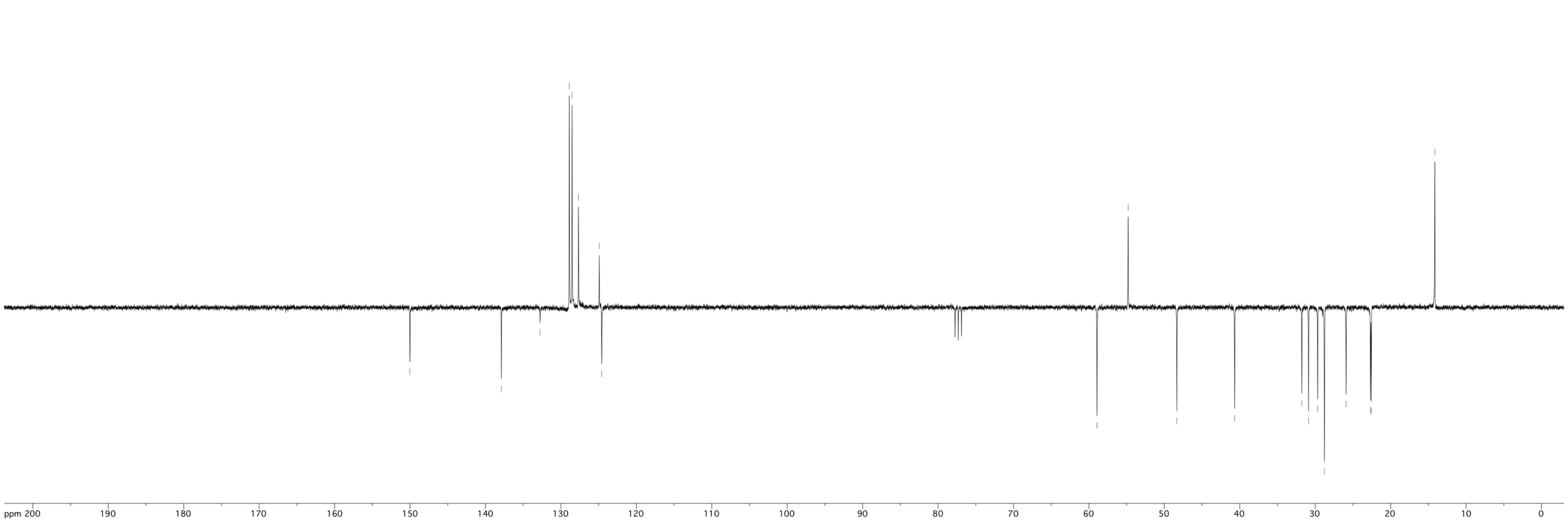

^{1}H NMR
300 MHz, $CDCl_3$

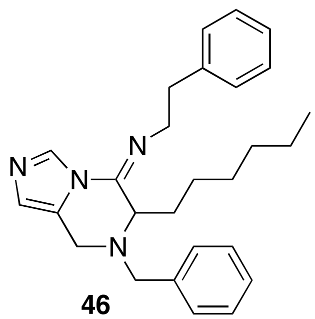

46

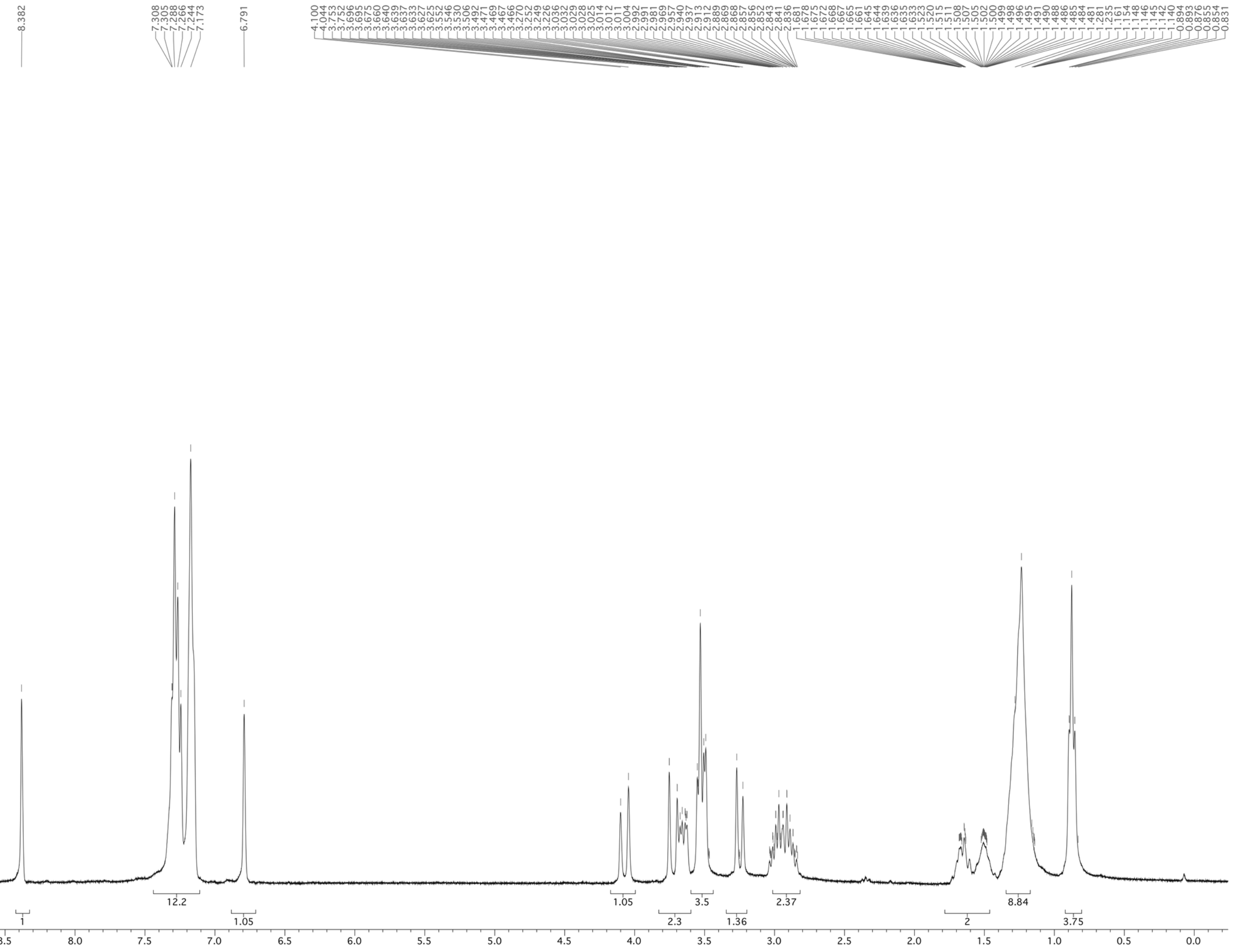

^{13}C NMR
75 MHz, $CDCl_3$

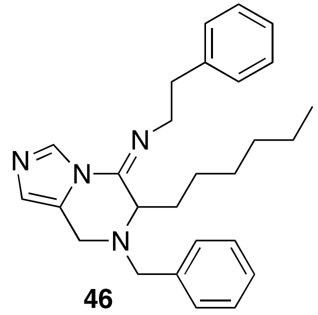

46

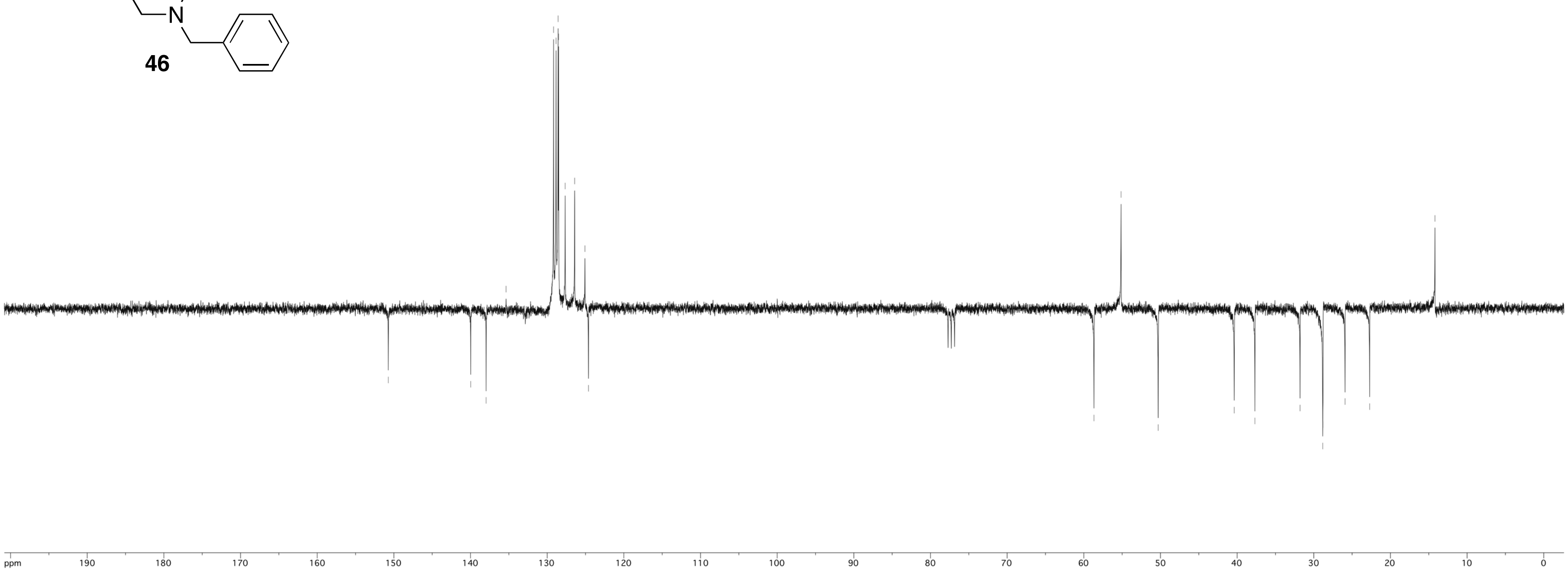

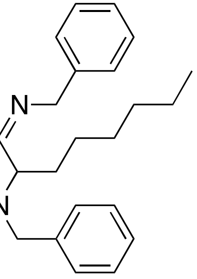

^1H NMR
300 MHz, $CDCl_3$

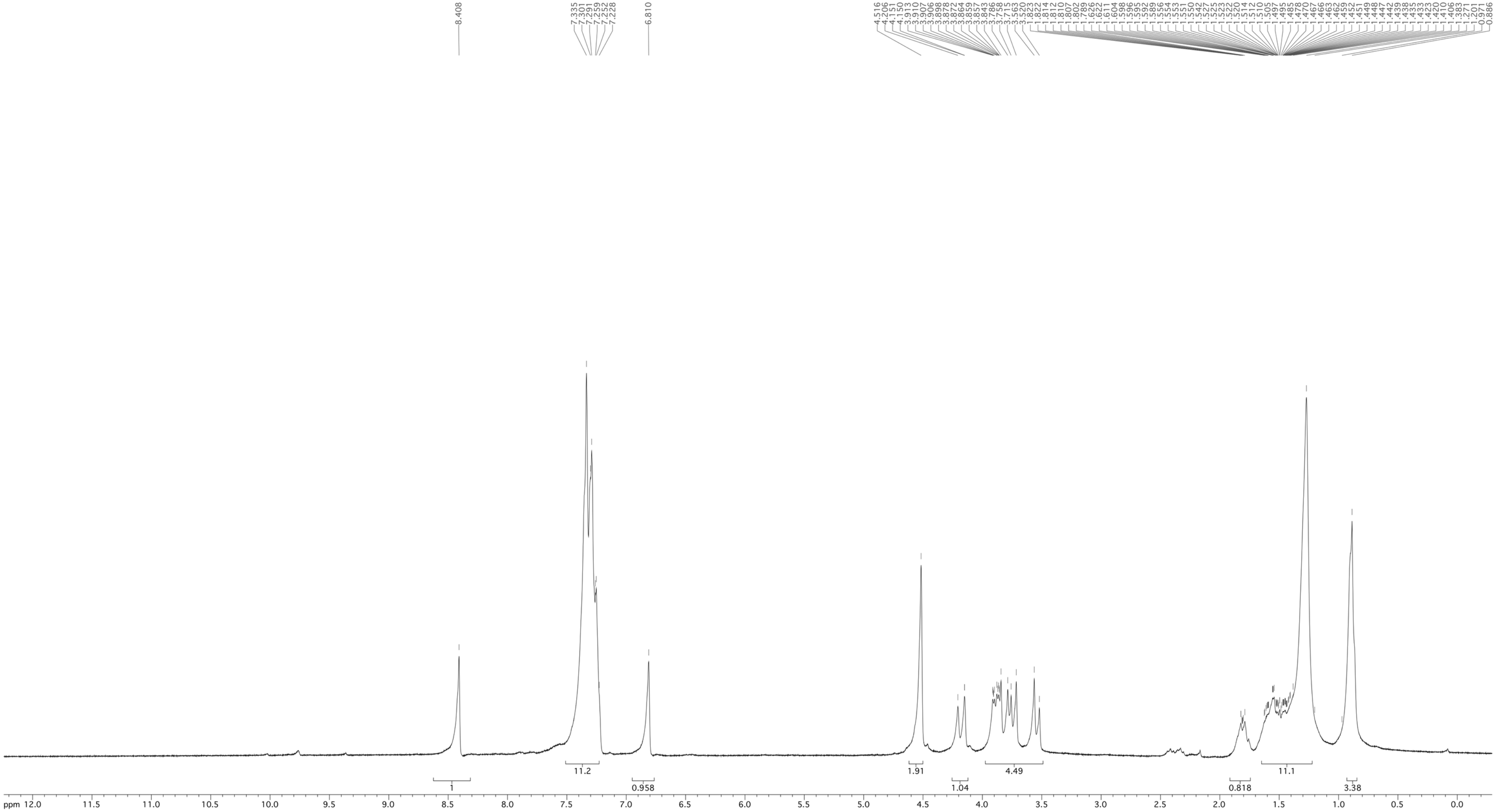

^{13}C NMR
75 MHz, $CDCl_3$

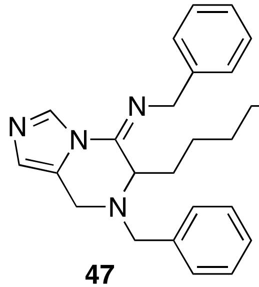

47

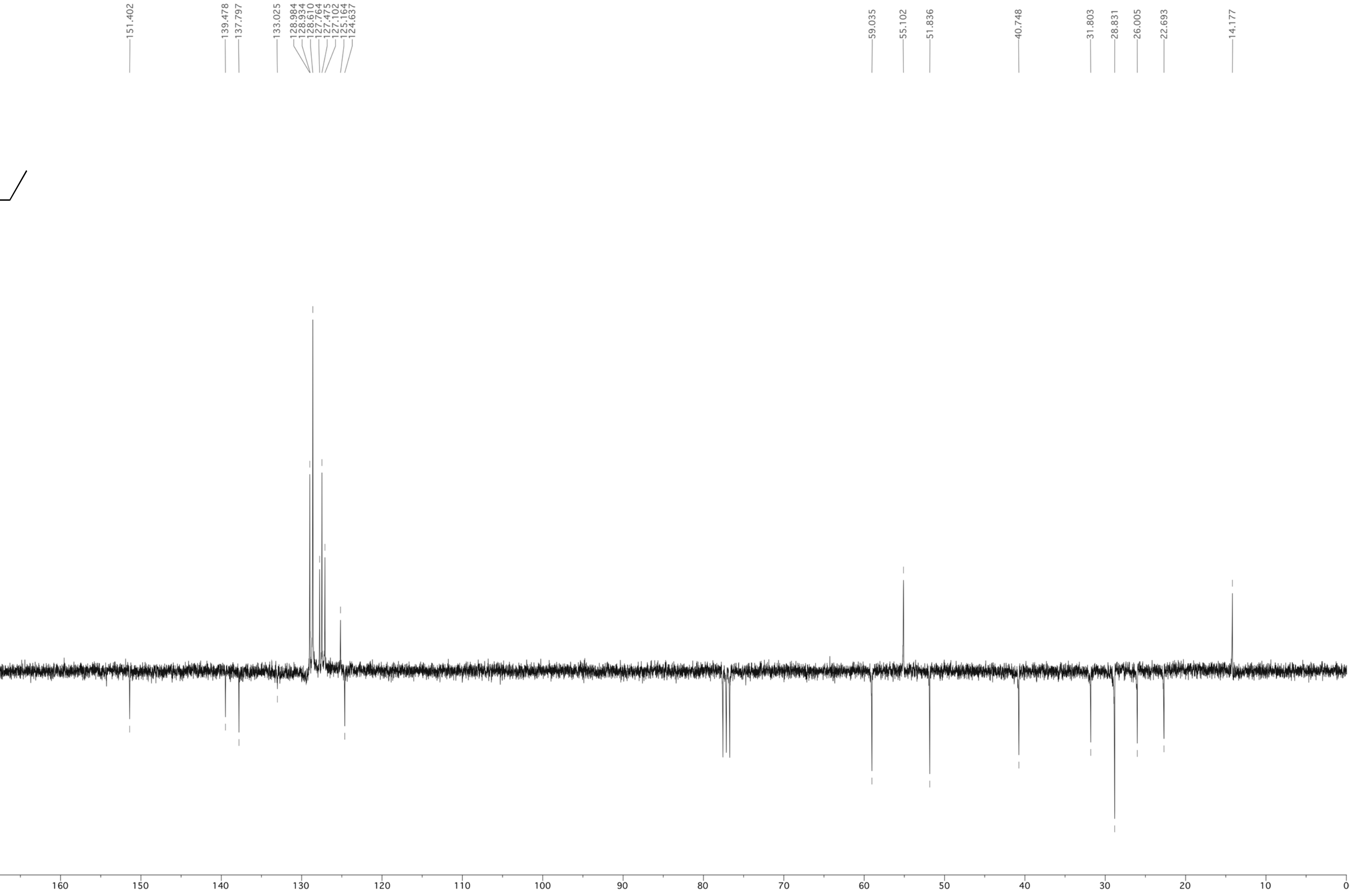

^{1}H NMR
300 MHz, $CDCl_3$

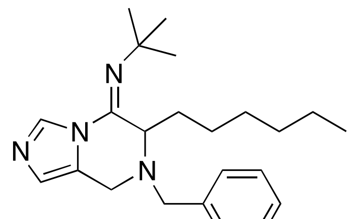

48

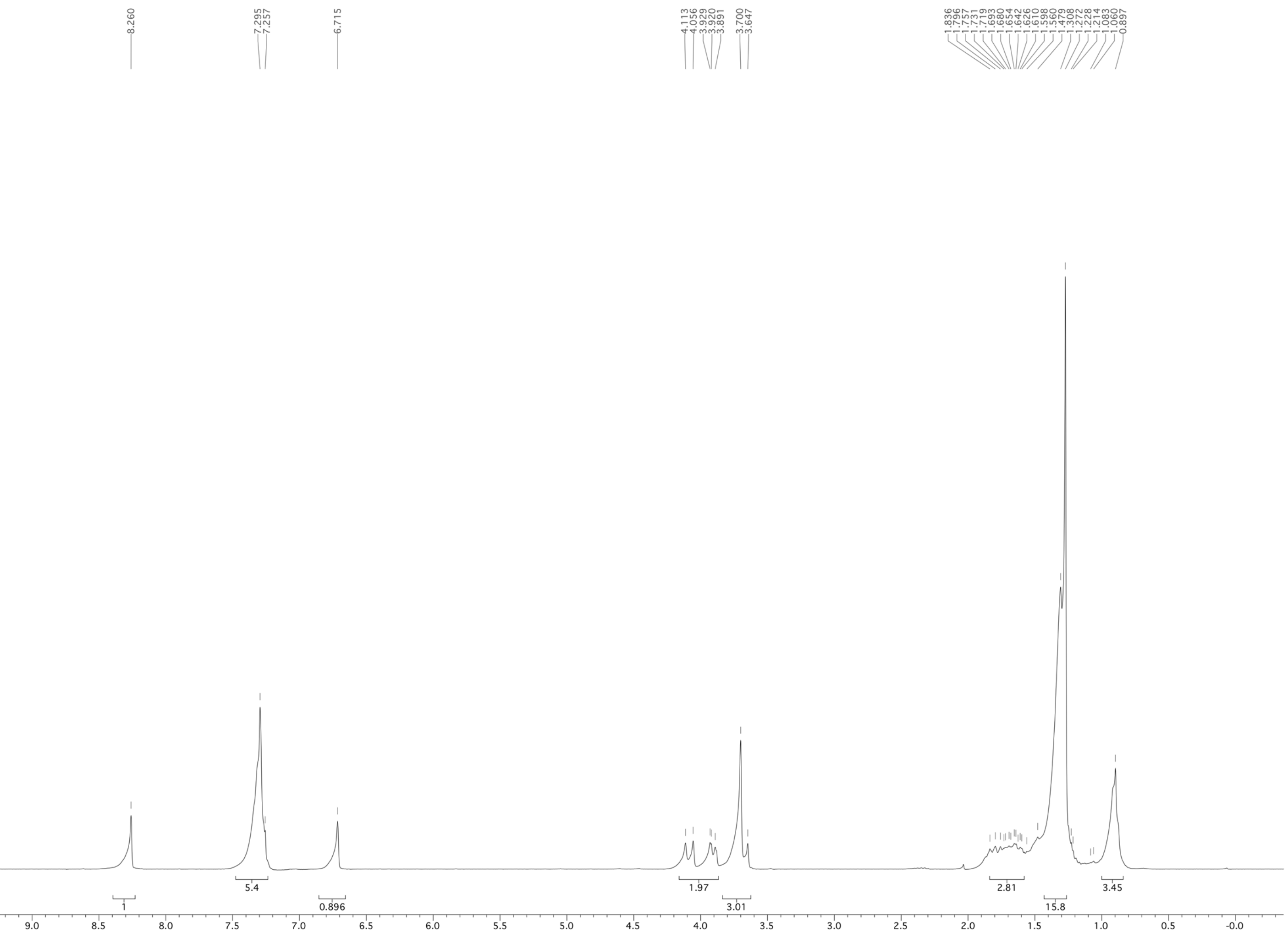

^{13}C NMR
75 MHz, $CDCl_3$

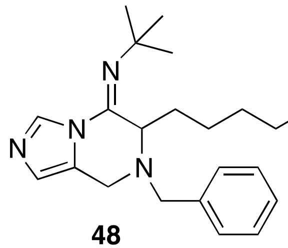

48

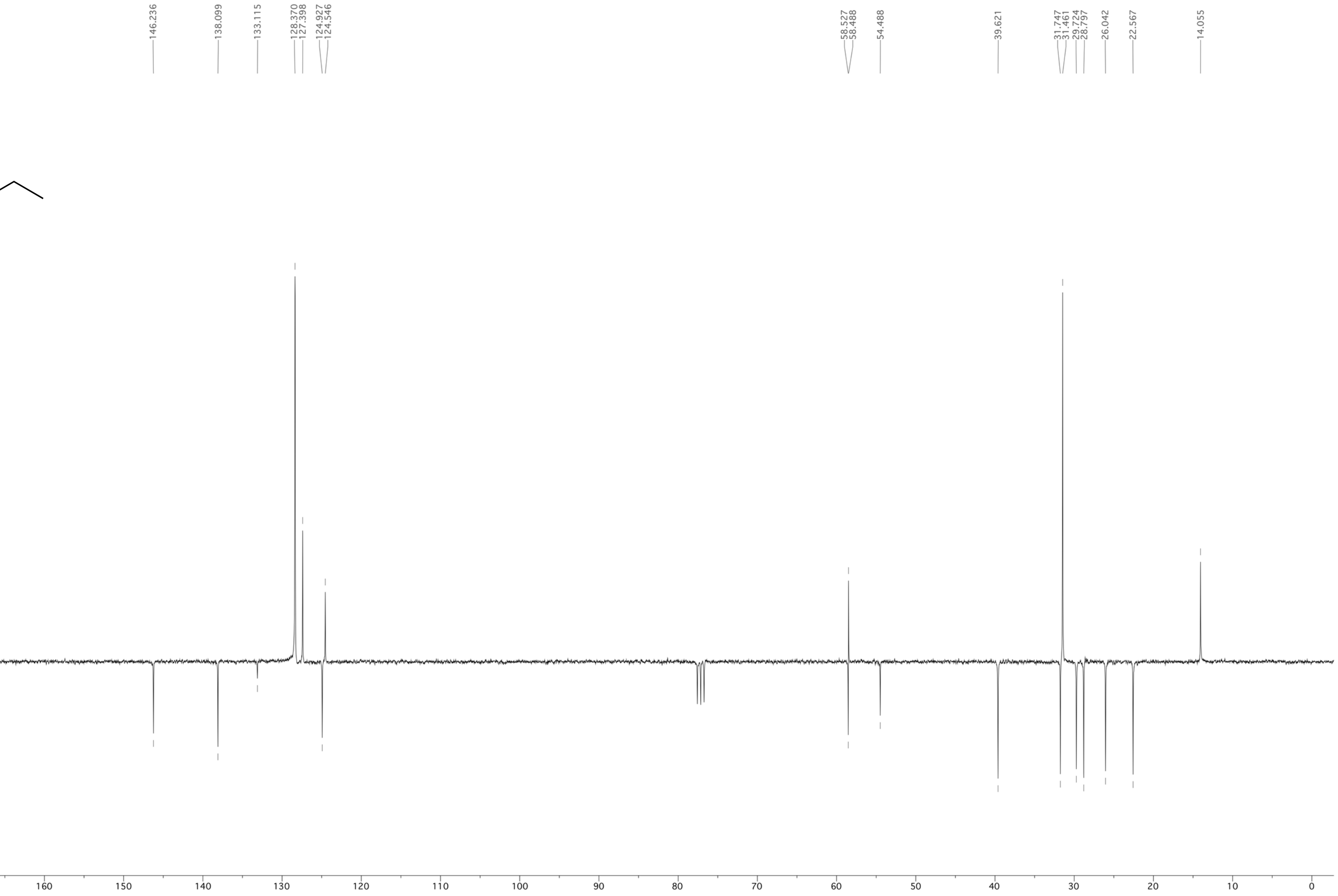

^{1}H NMR
300 MHz, $CDCl_3$

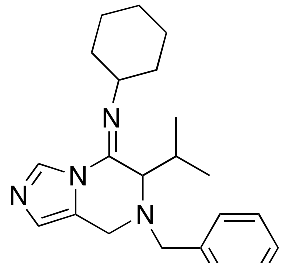

49

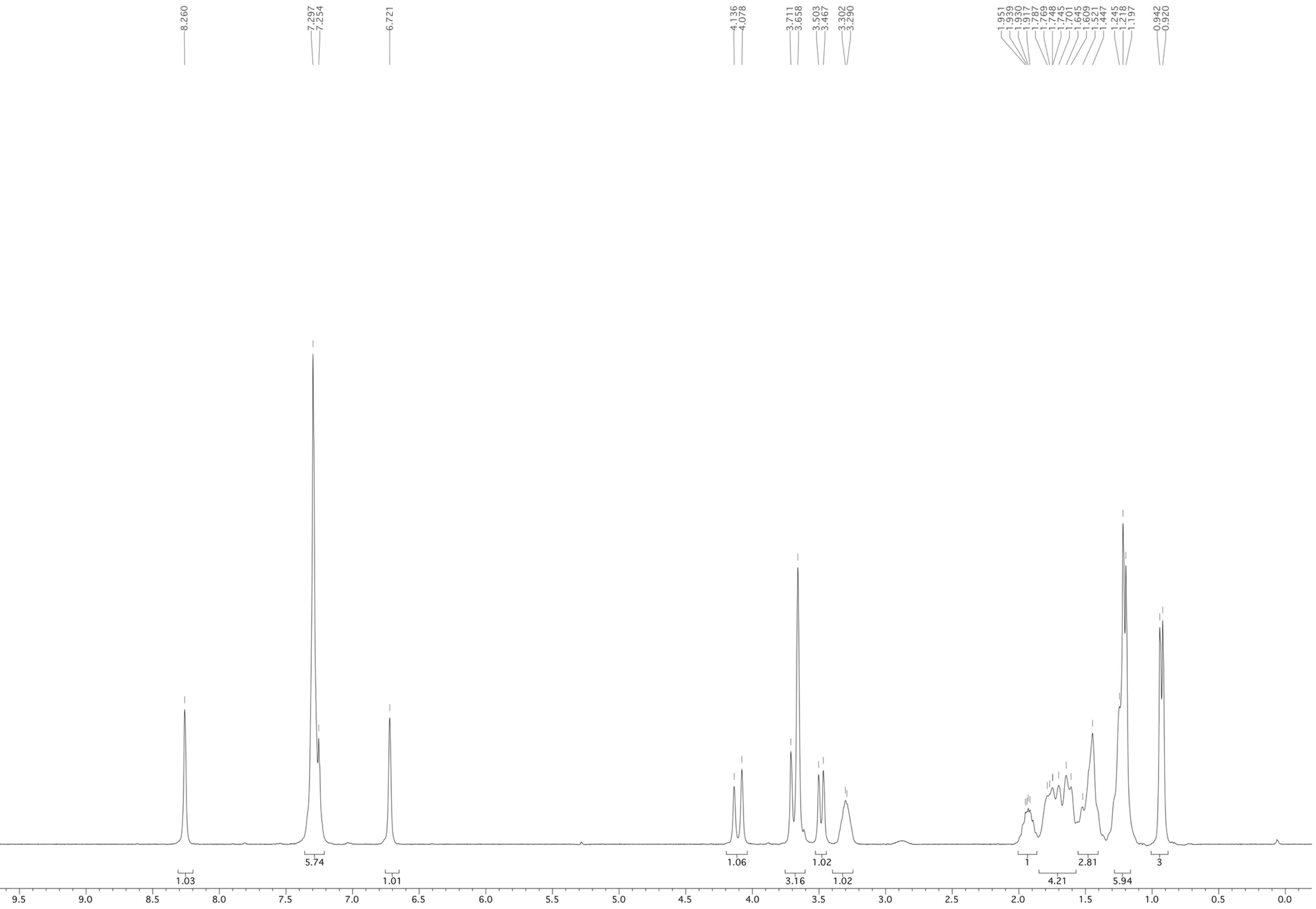

^{13}C NMR
75 MHz, $CDCl_3$

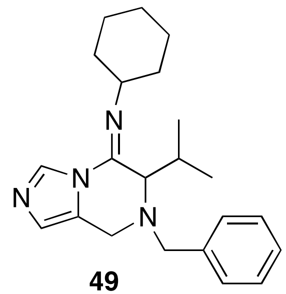

49

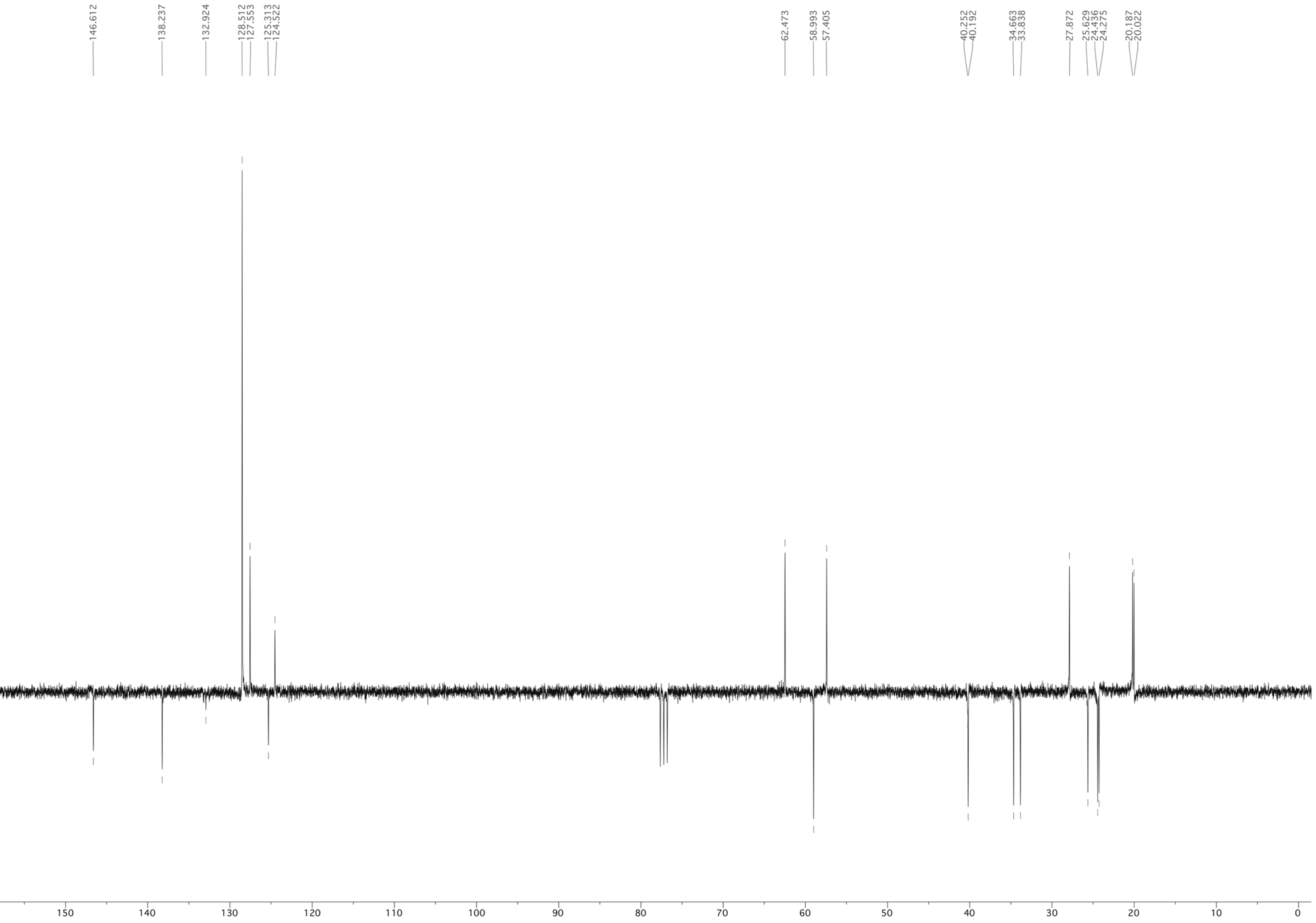

^{1}H NMR
300 MHz, $CDCl_3$

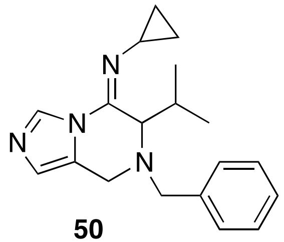

50

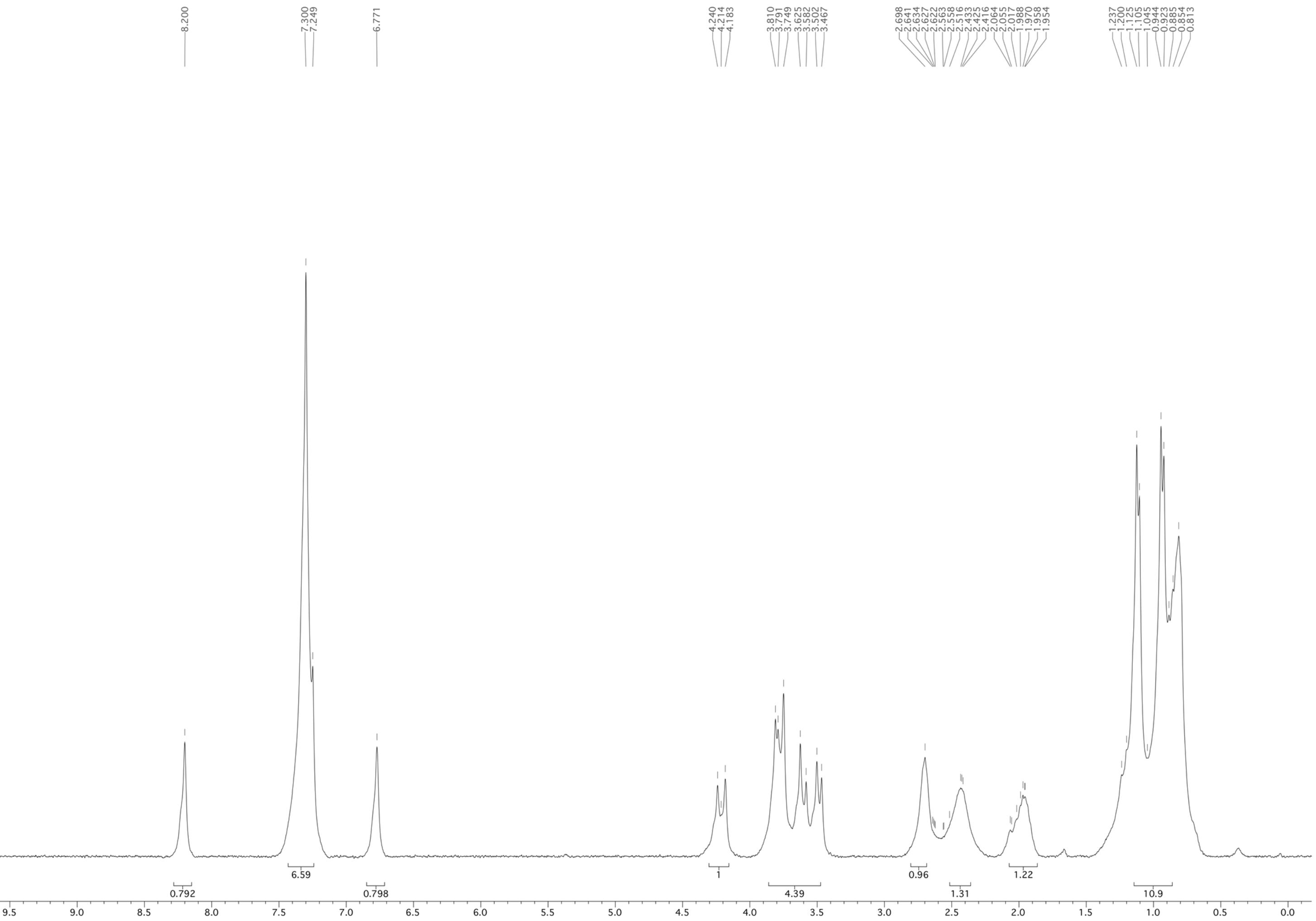

^{13}C NMR
75 MHz, $CDCl_3$

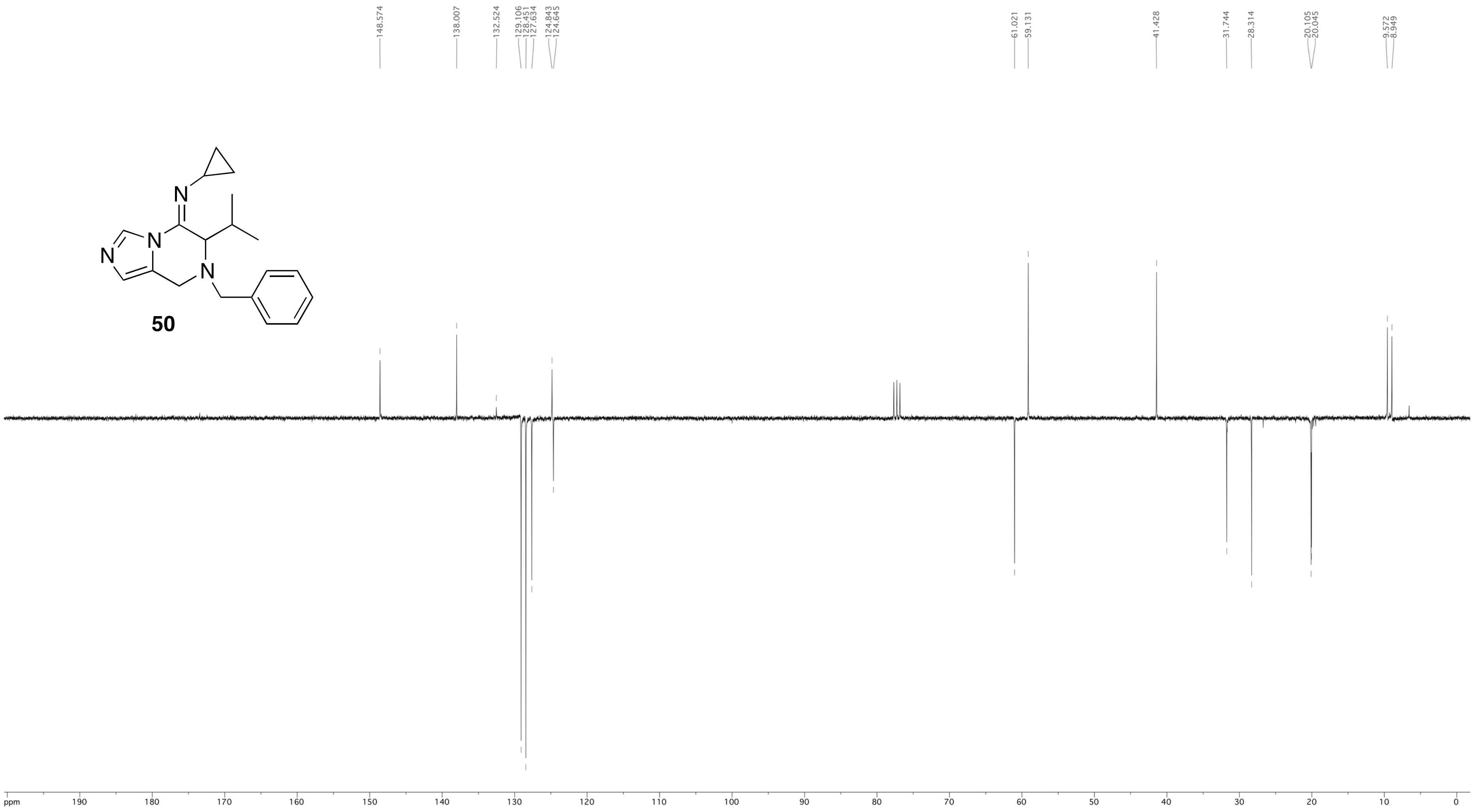

50

^{1}H NMR
300 MHz, $CDCl_3$

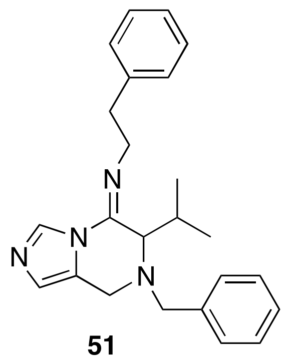

51

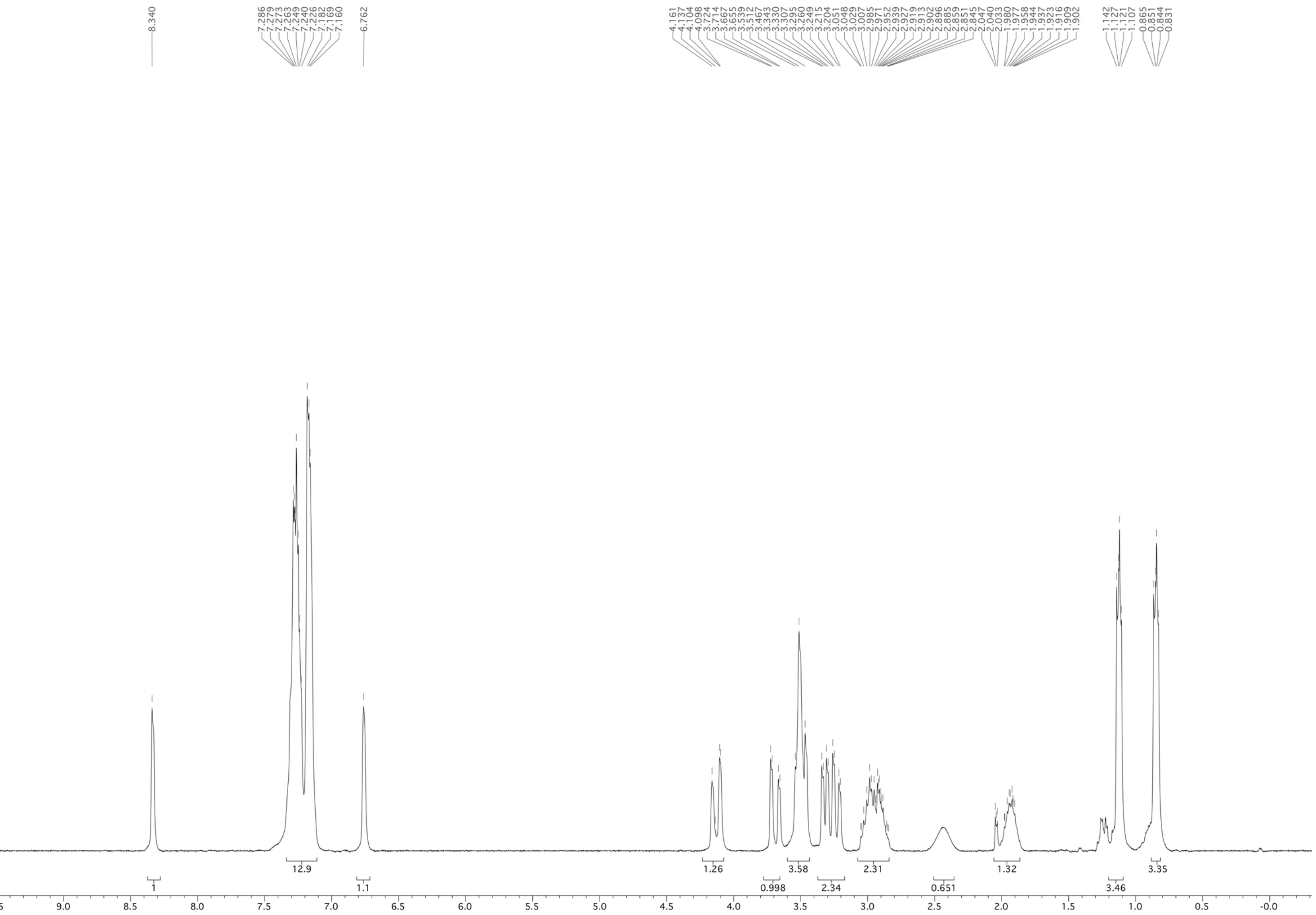

^{13}C NMR
75 MHz, $CDCl_3$

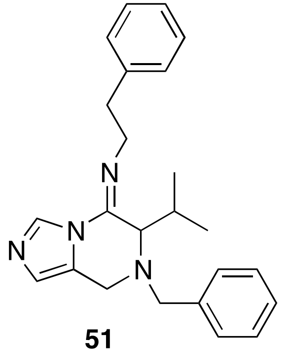

51

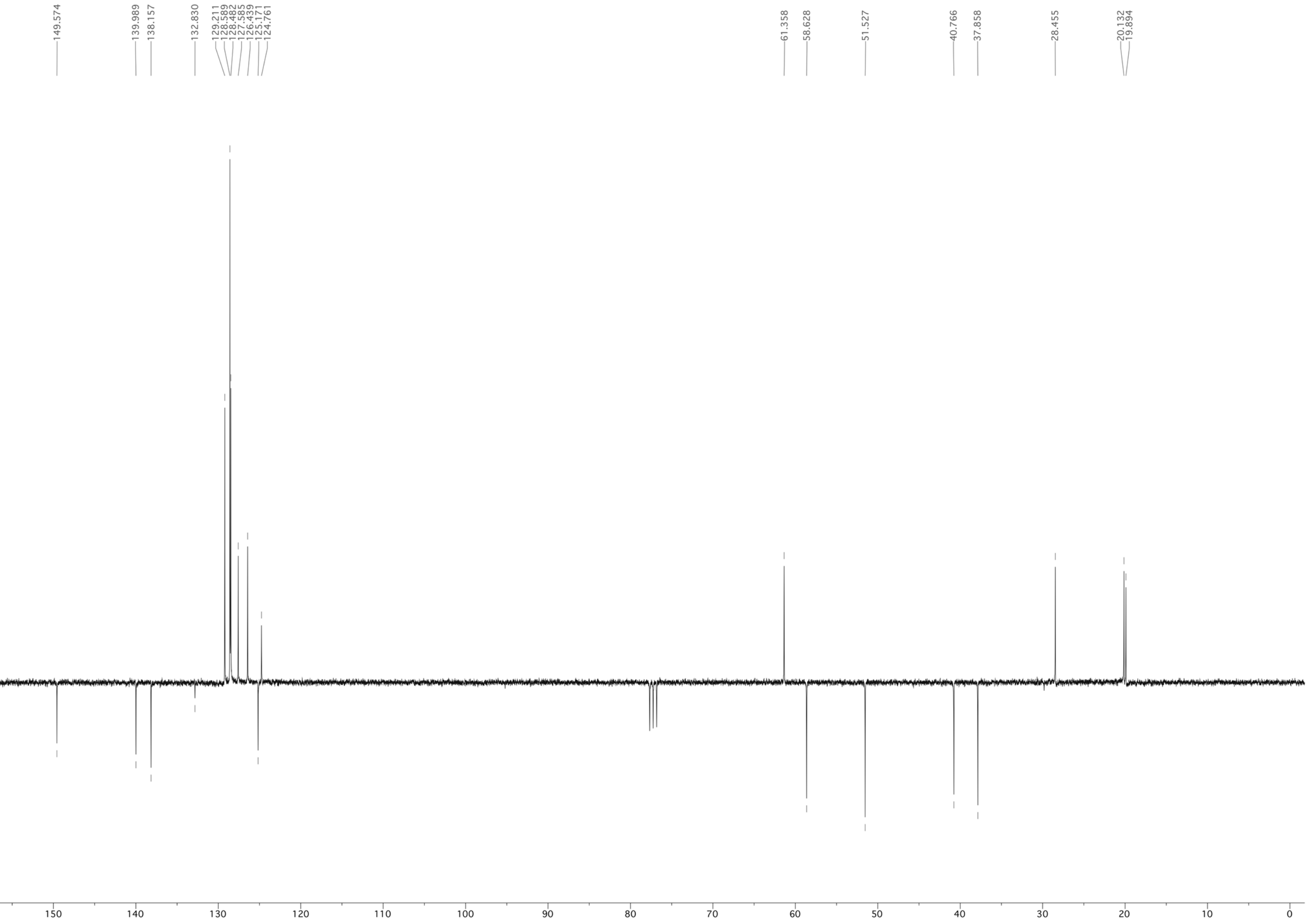

^{1}H NMR
300 MHz, $CDCl_3$

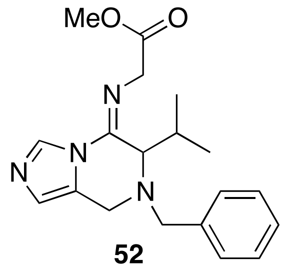

52

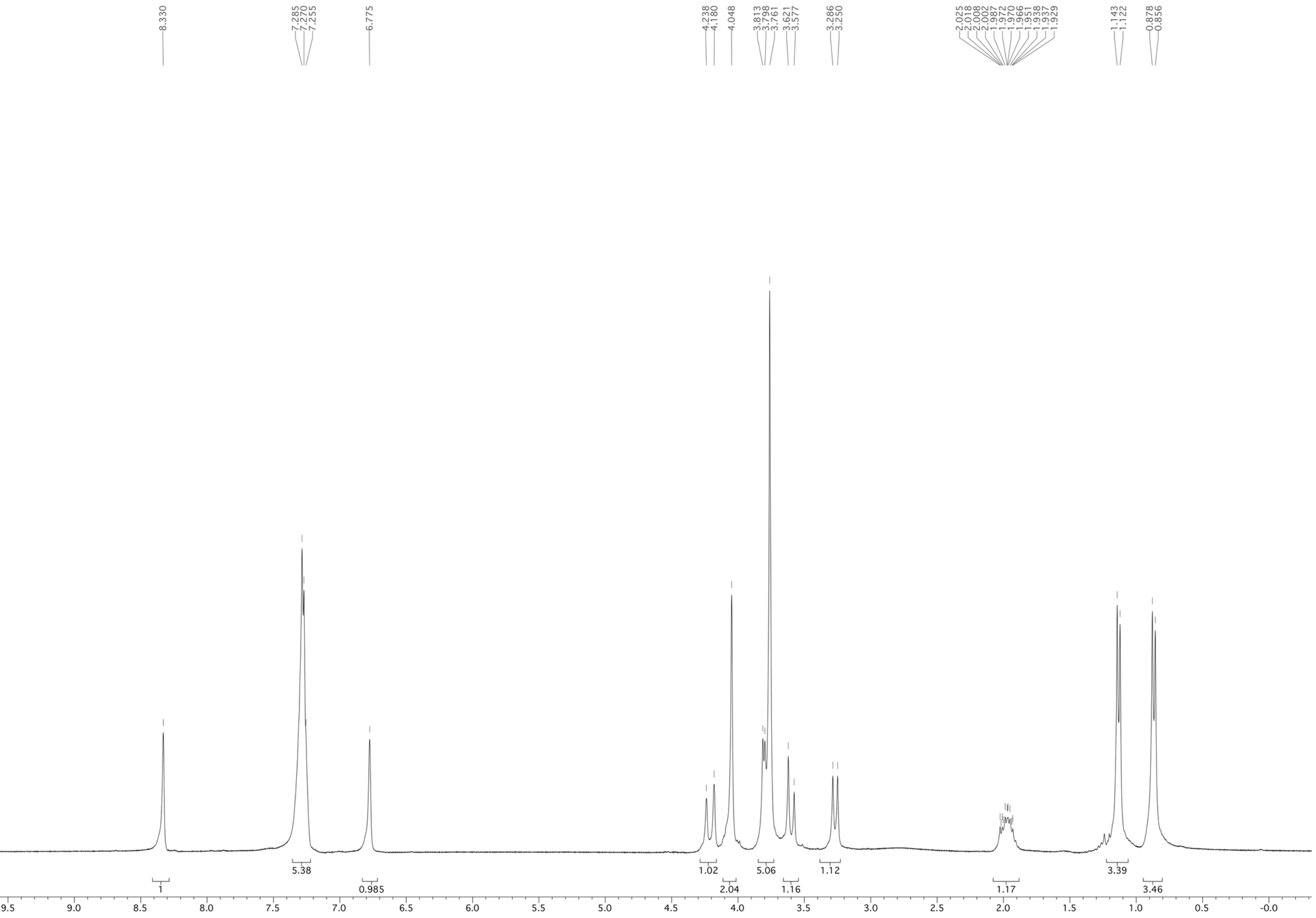

^{13}C NMR
75 MHz, $CDCl_3$

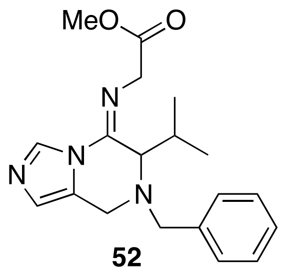

52

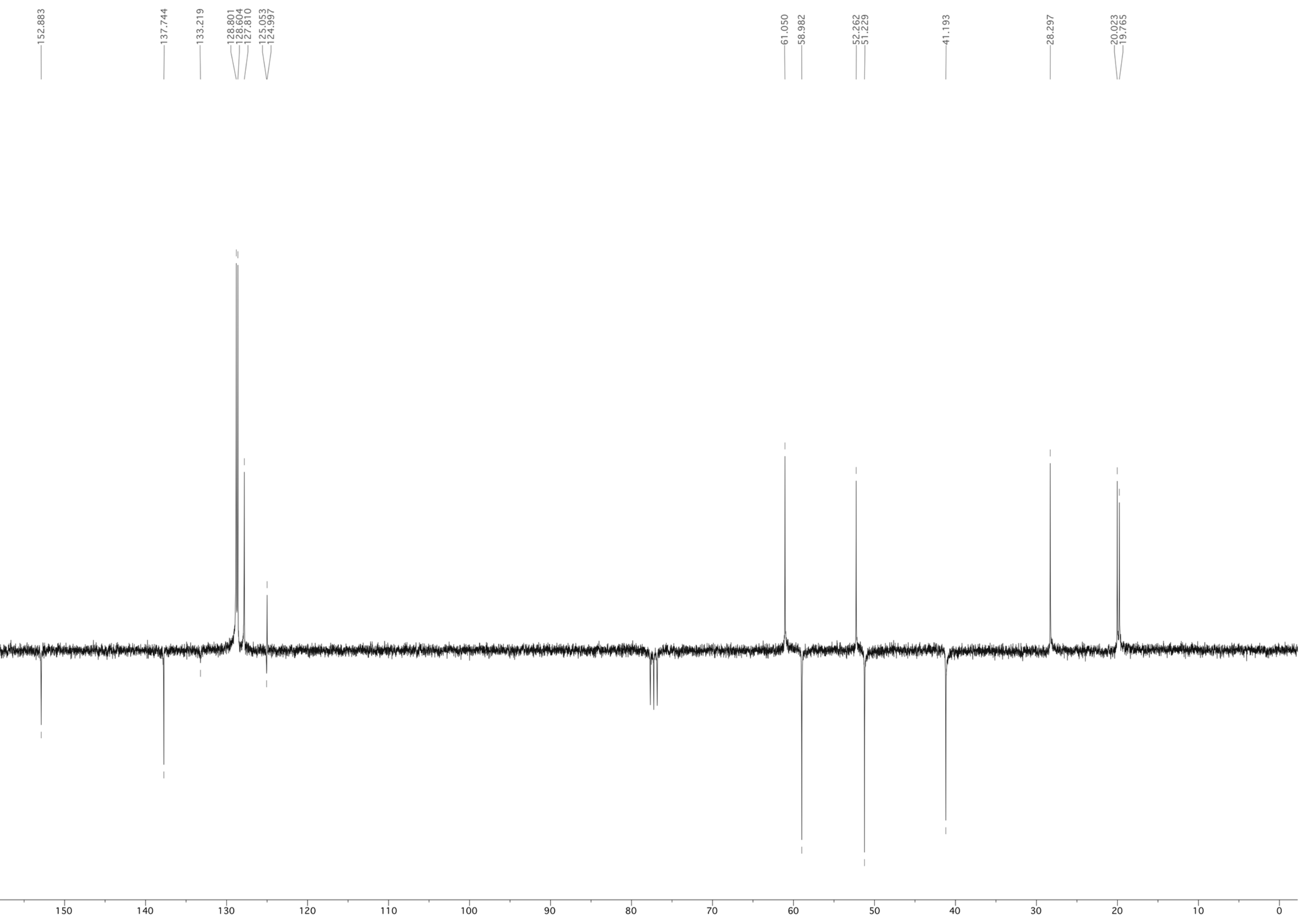

^{1}H NMR
300 MHz, $CDCl_3$

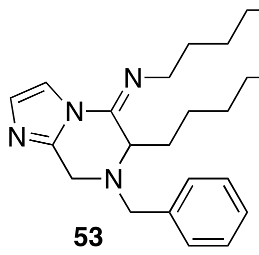

53

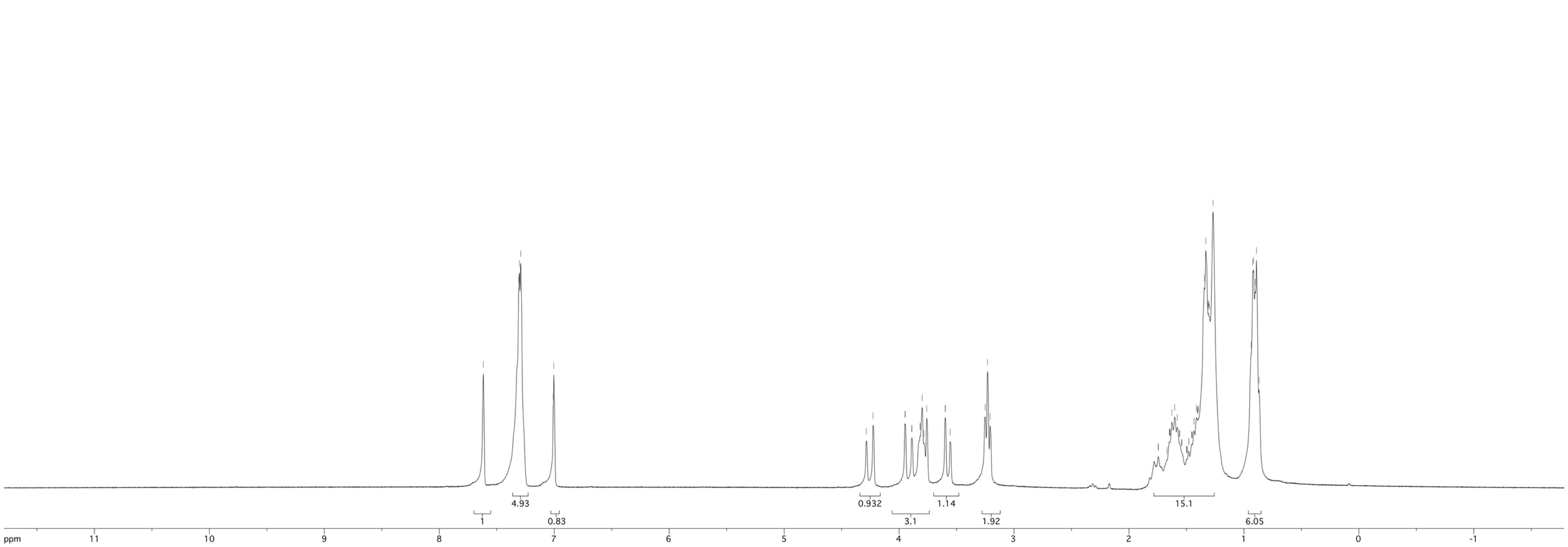

^{13}C NMR
75 MHz, $CDCl_3$

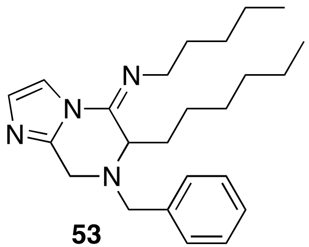

53

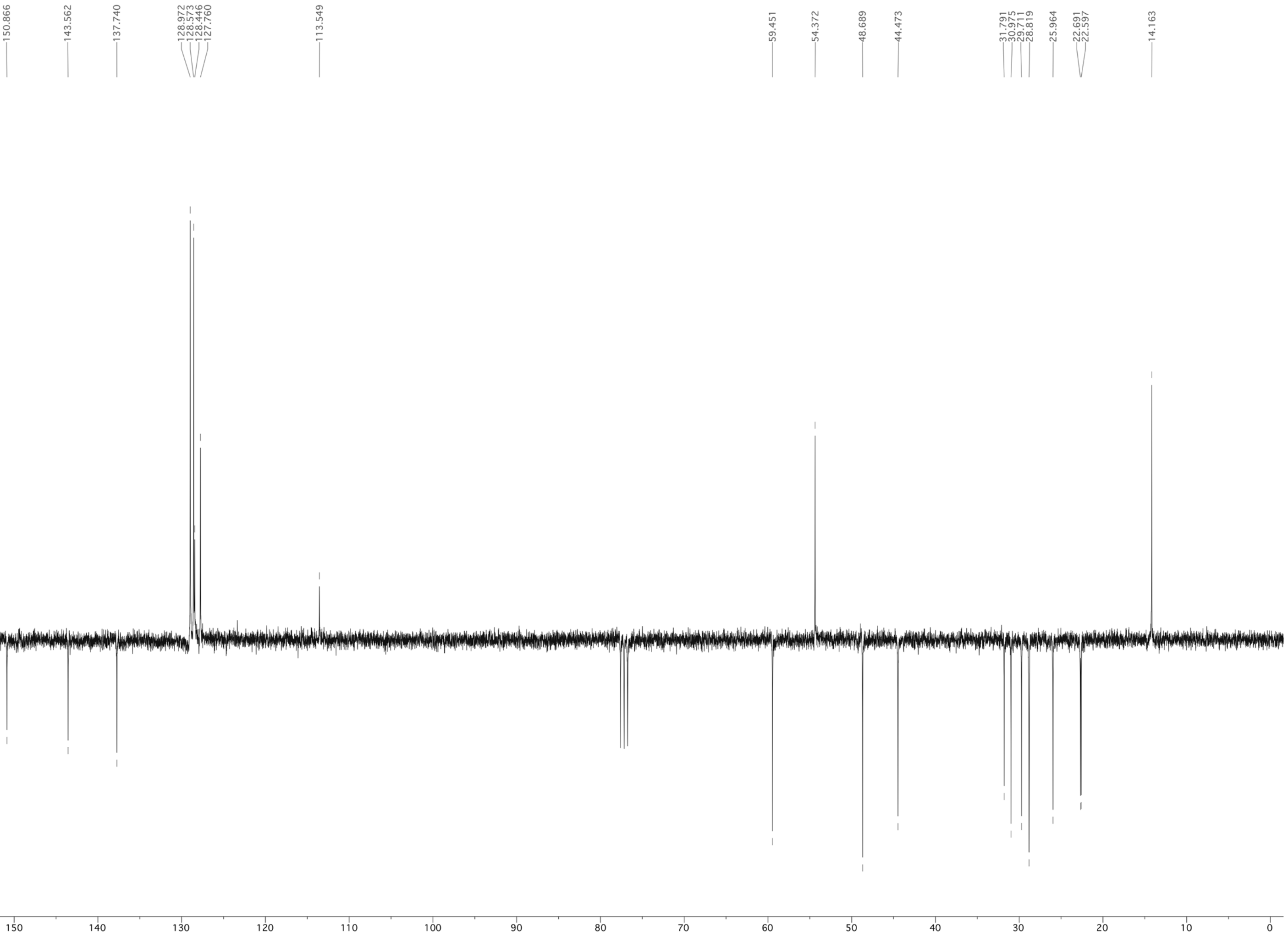

^{1}H NMR
300 MHz, $CDCl_3$

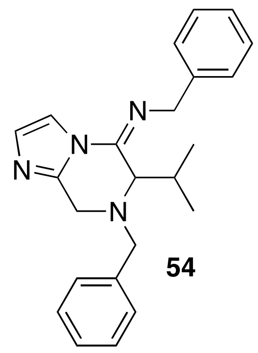

54

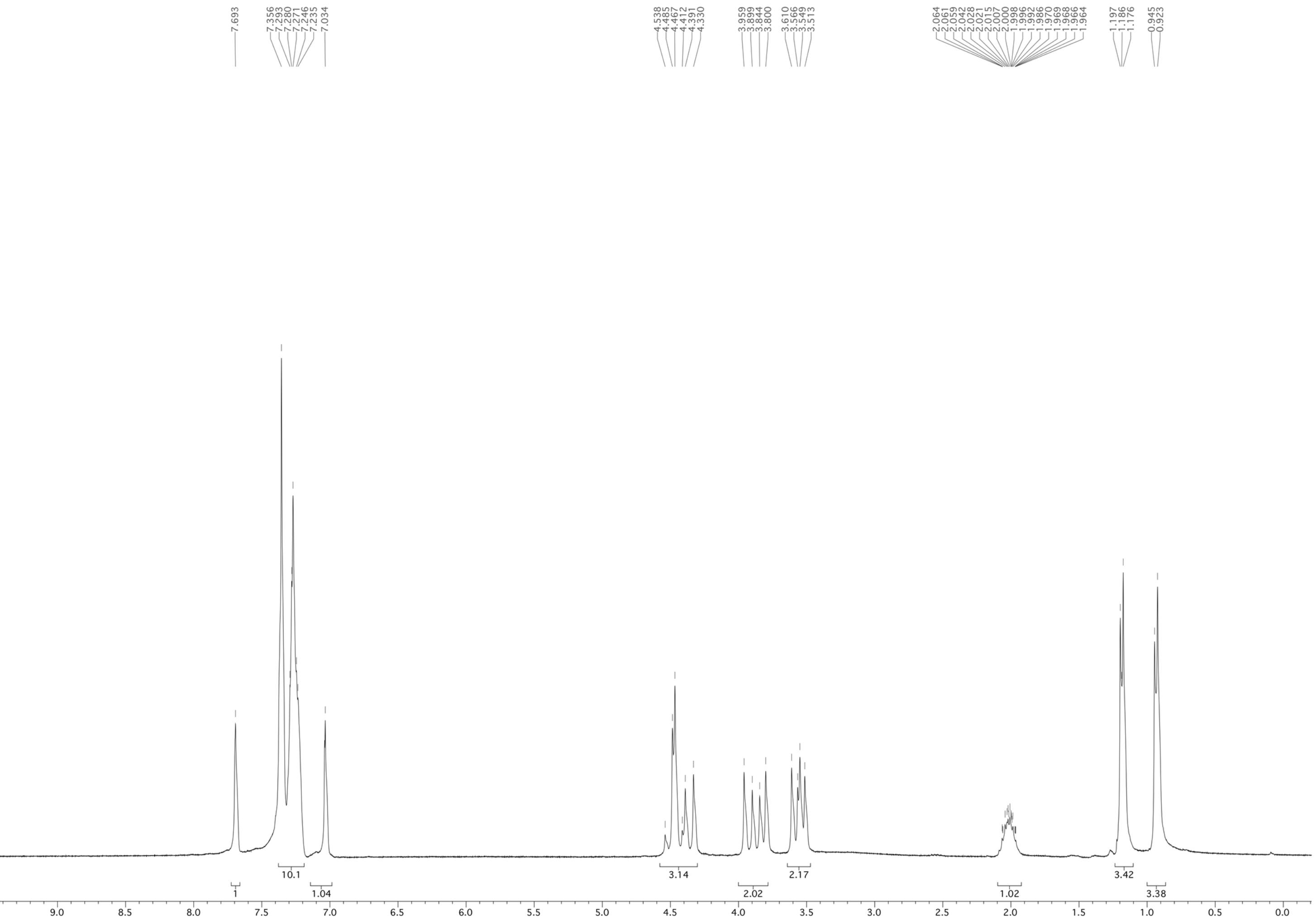

^{13}C NMR
75 MHz, $CDCl_3$

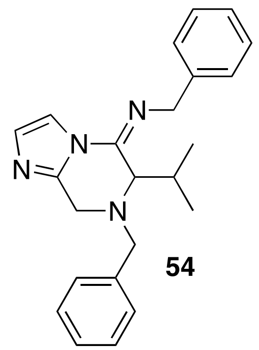

54

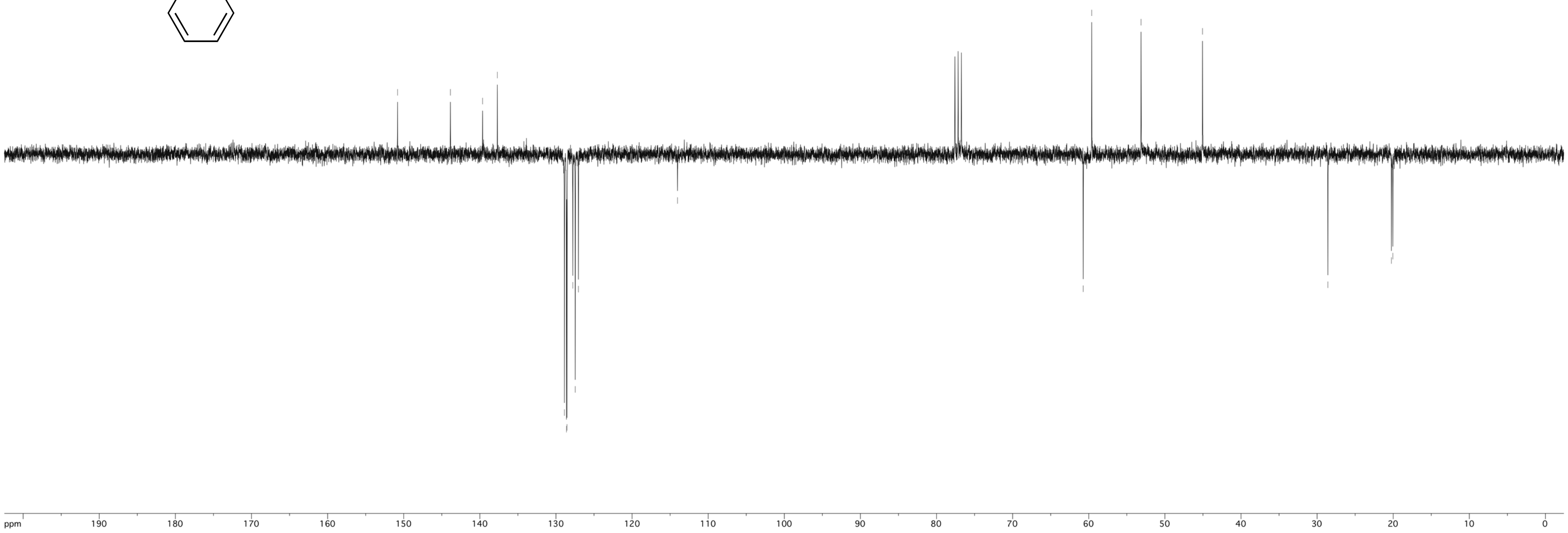

^{1}H NMR
300 MHz, $CDCl_3$

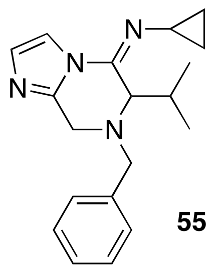

55

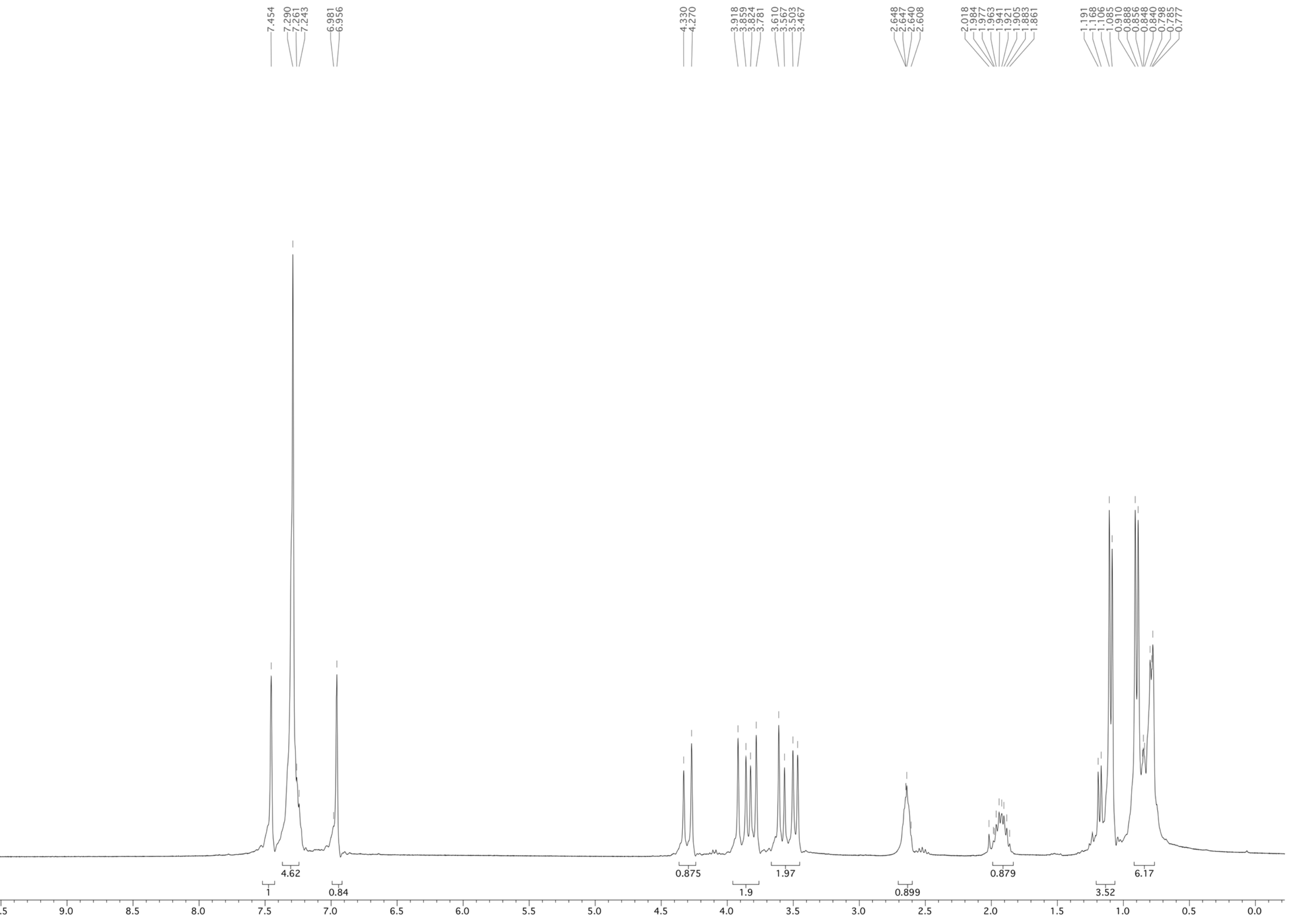

^{13}C NMR
75 MHz, $CDCl_3$

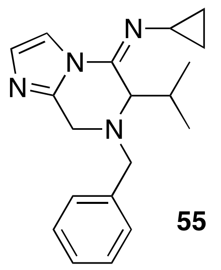

55

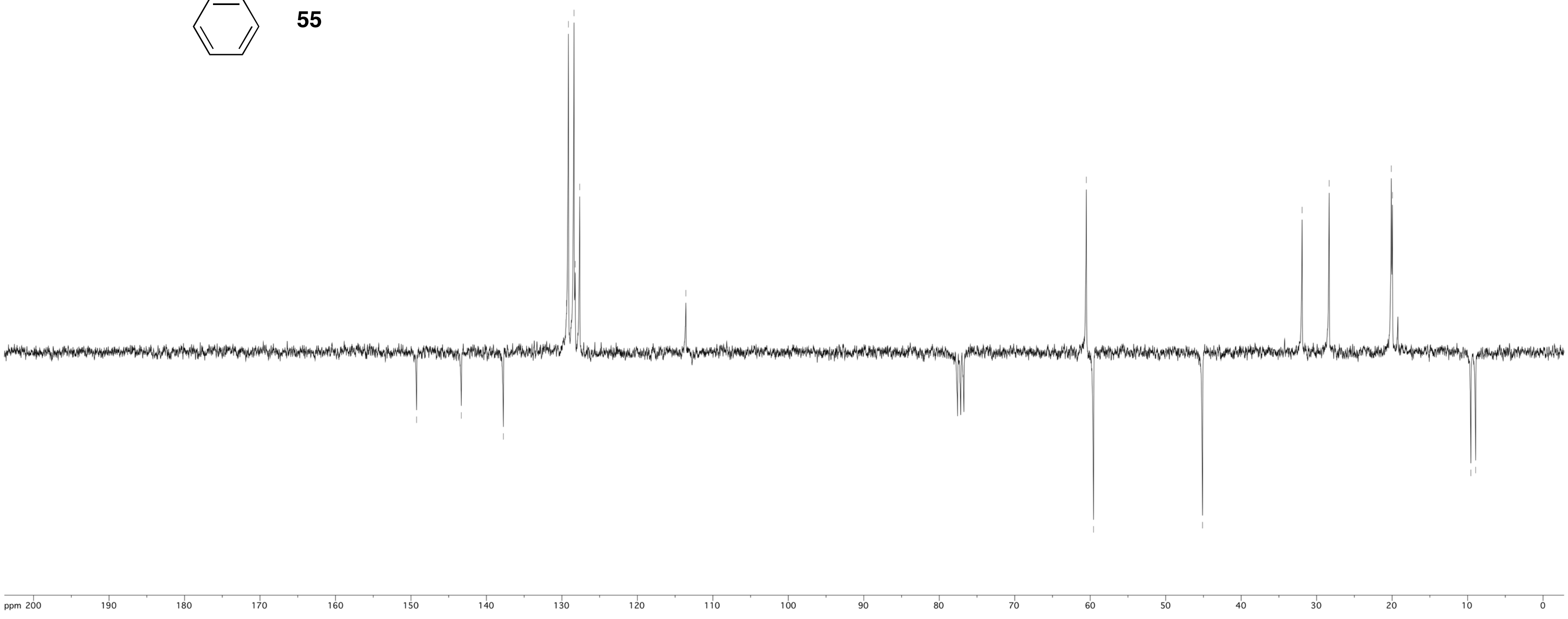

^{1}H NMR
300 MHz, $CDCl_3$

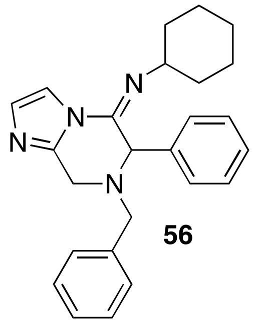

56

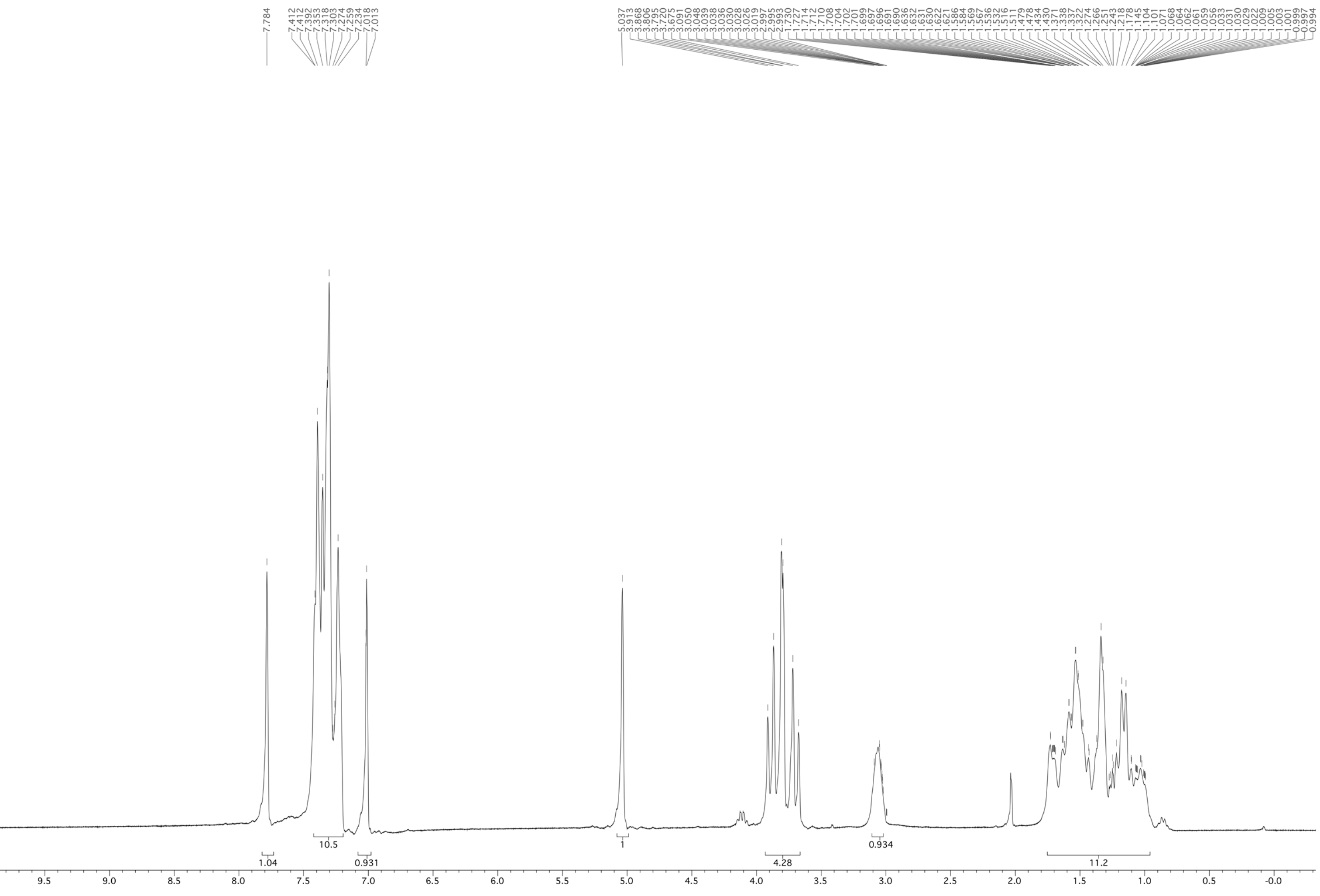

^{13}C NMR
75 MHz, $CDCl_3$

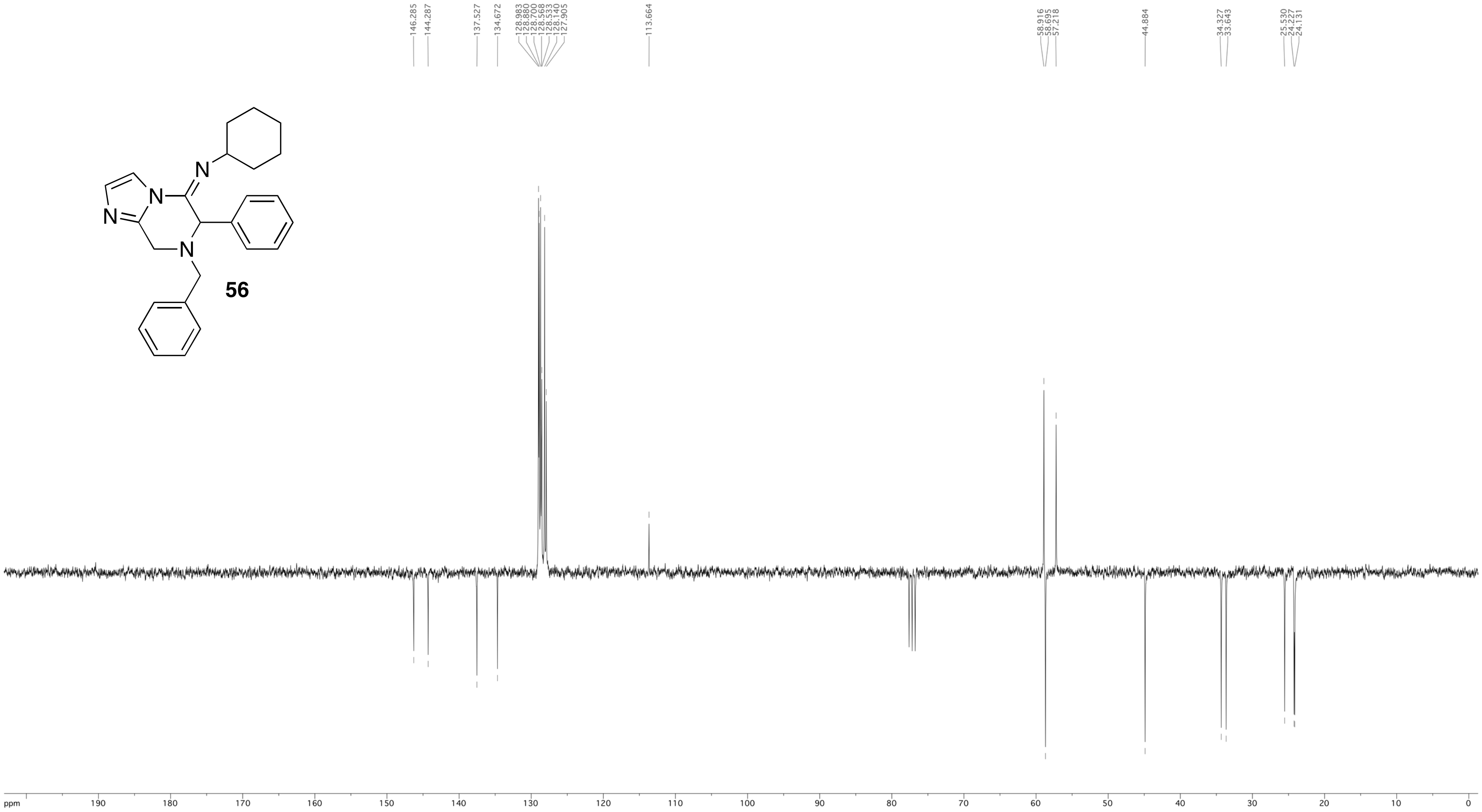

56

^{1}H NMR
300 MHz, $CDCl_3$

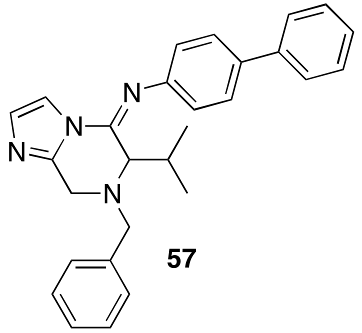

57

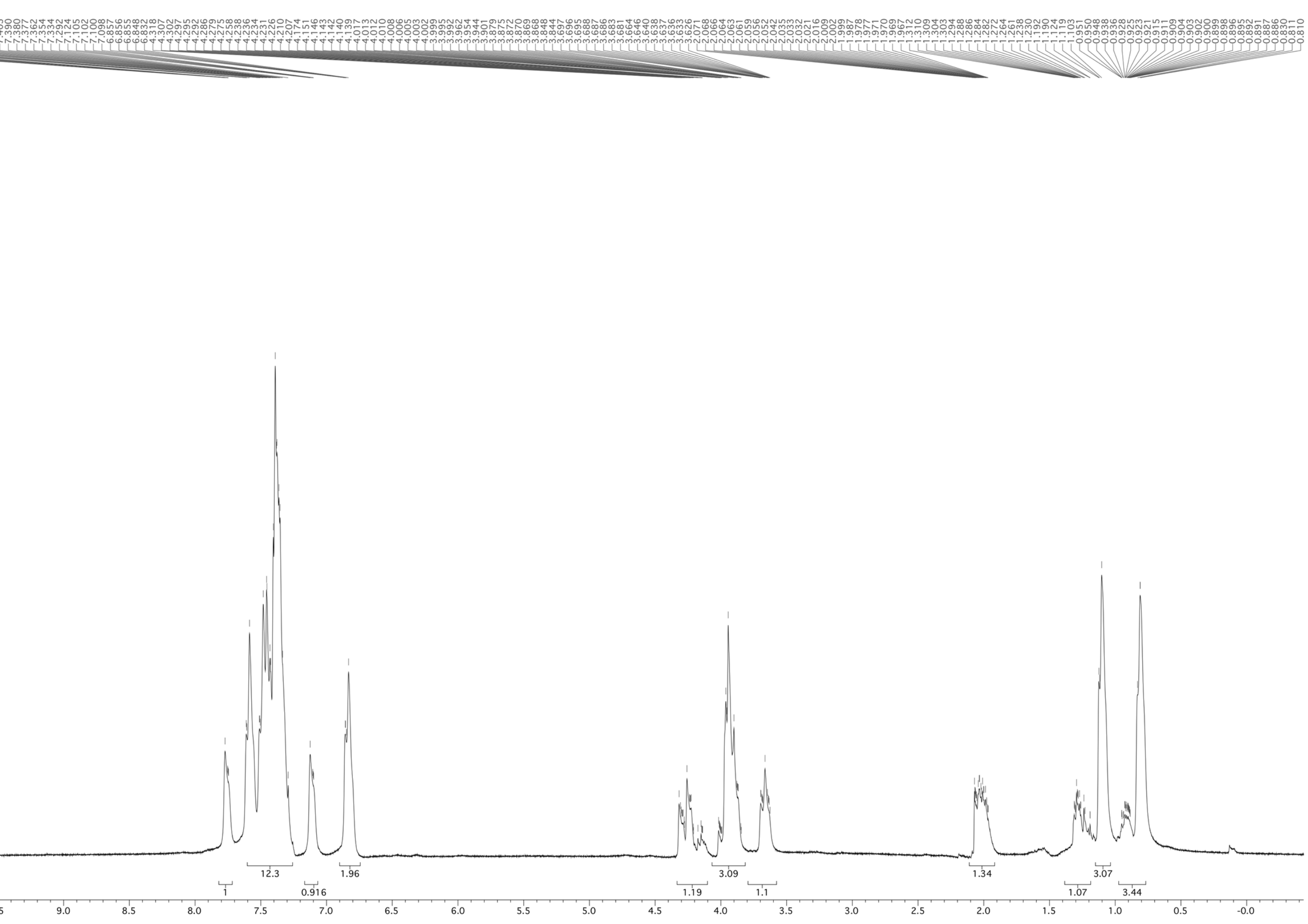

^{13}C NMR
75 MHz, $CDCl_3$

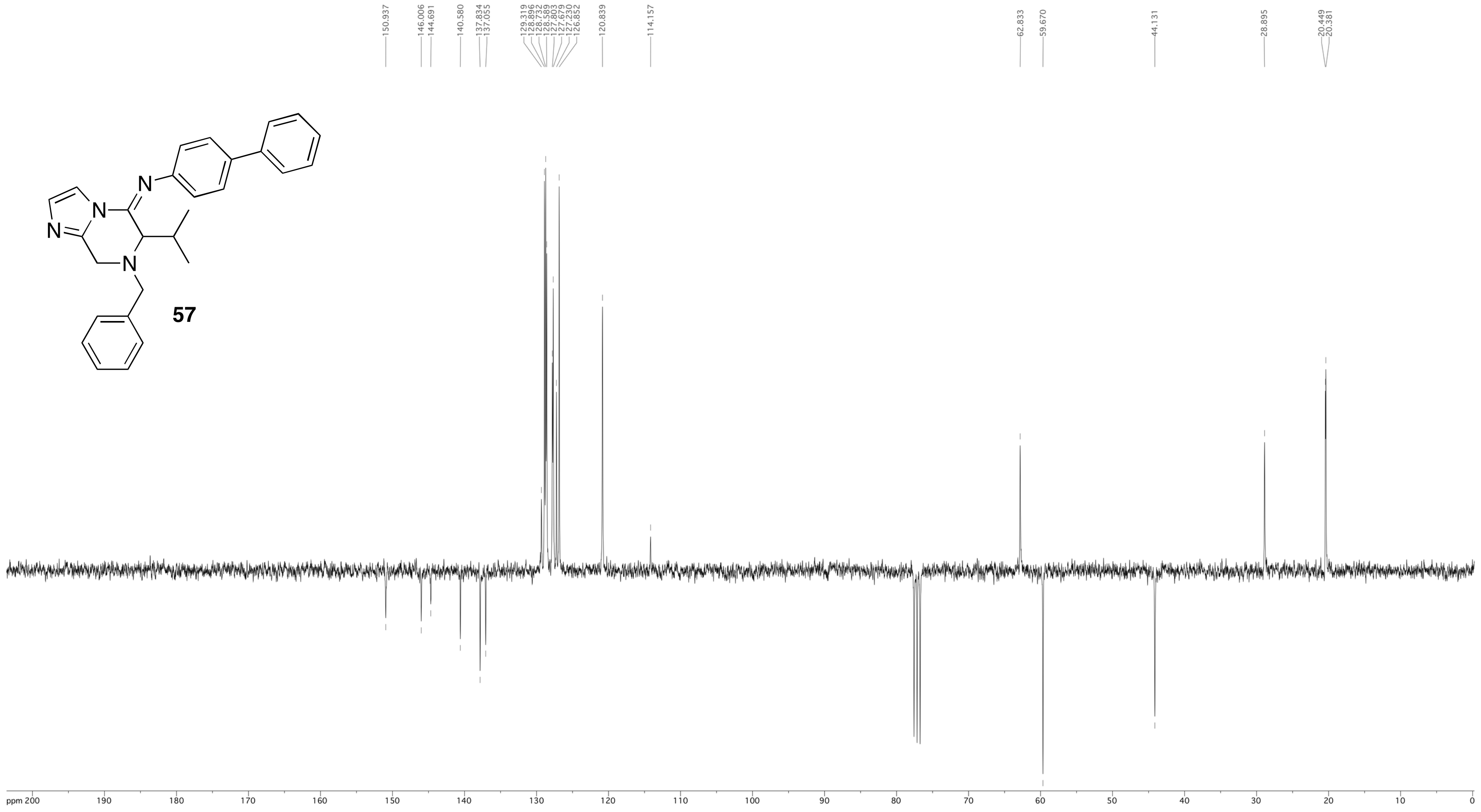

^{1}H NMR
300 MHz, $CDCl_3$

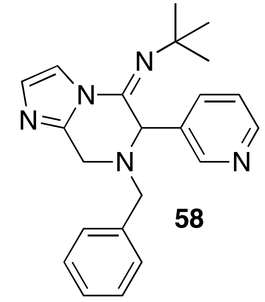

58

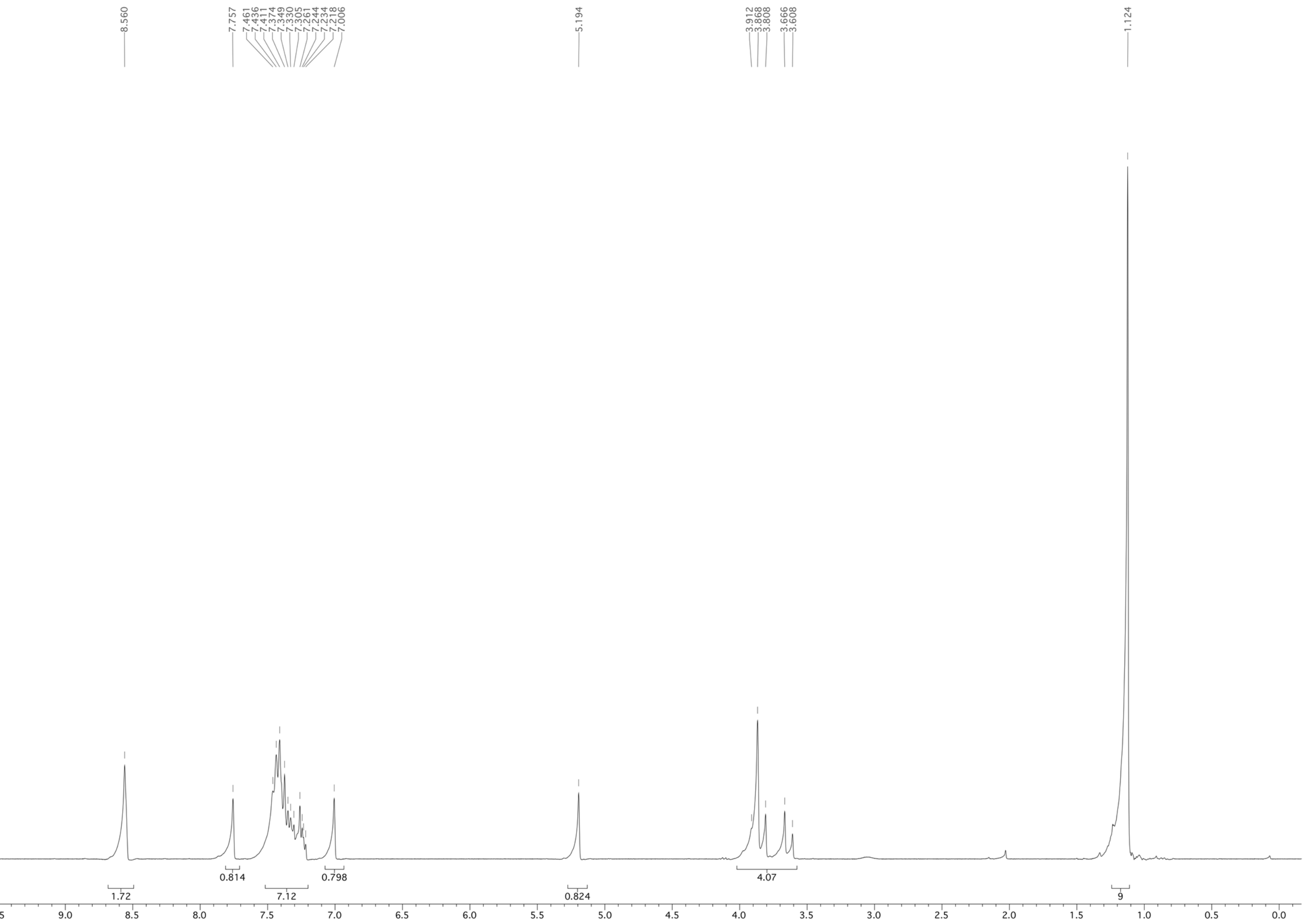

^{13}C NMR
75 MHz, $CDCl_3$

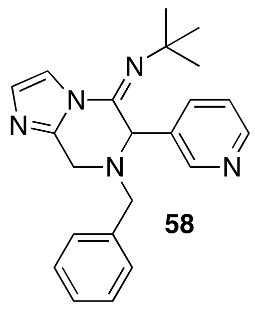

58

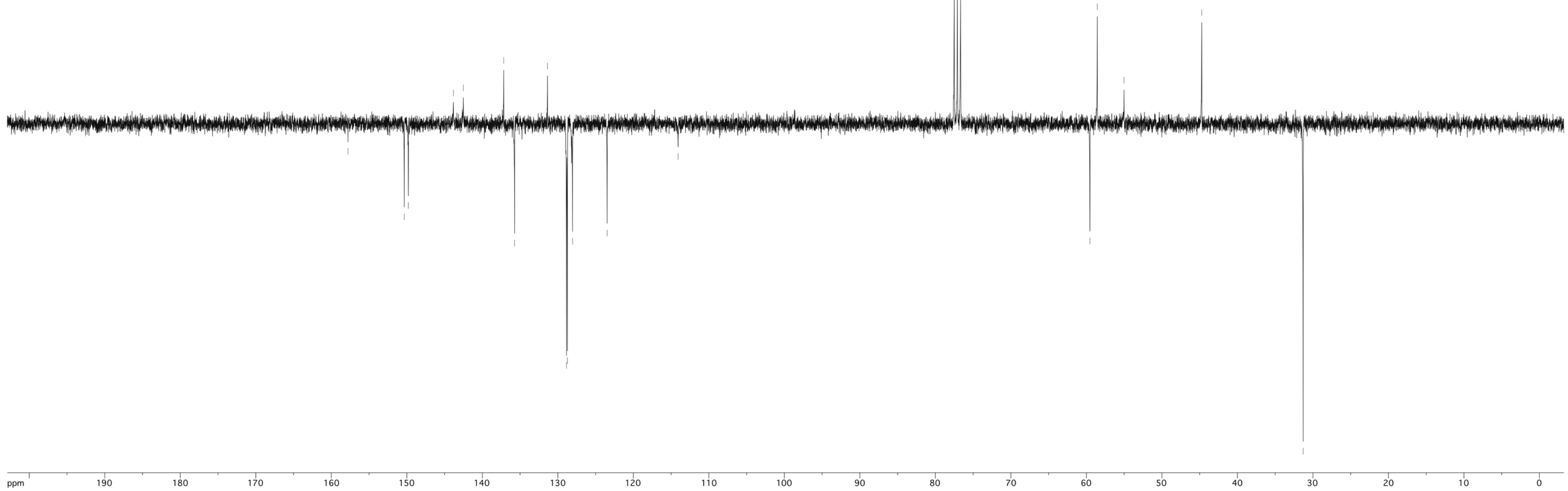

^1H NMR
300 MHz, $CDCl_3$

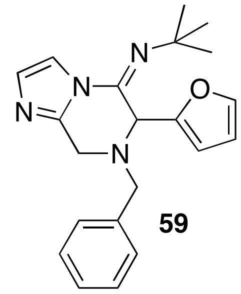

59

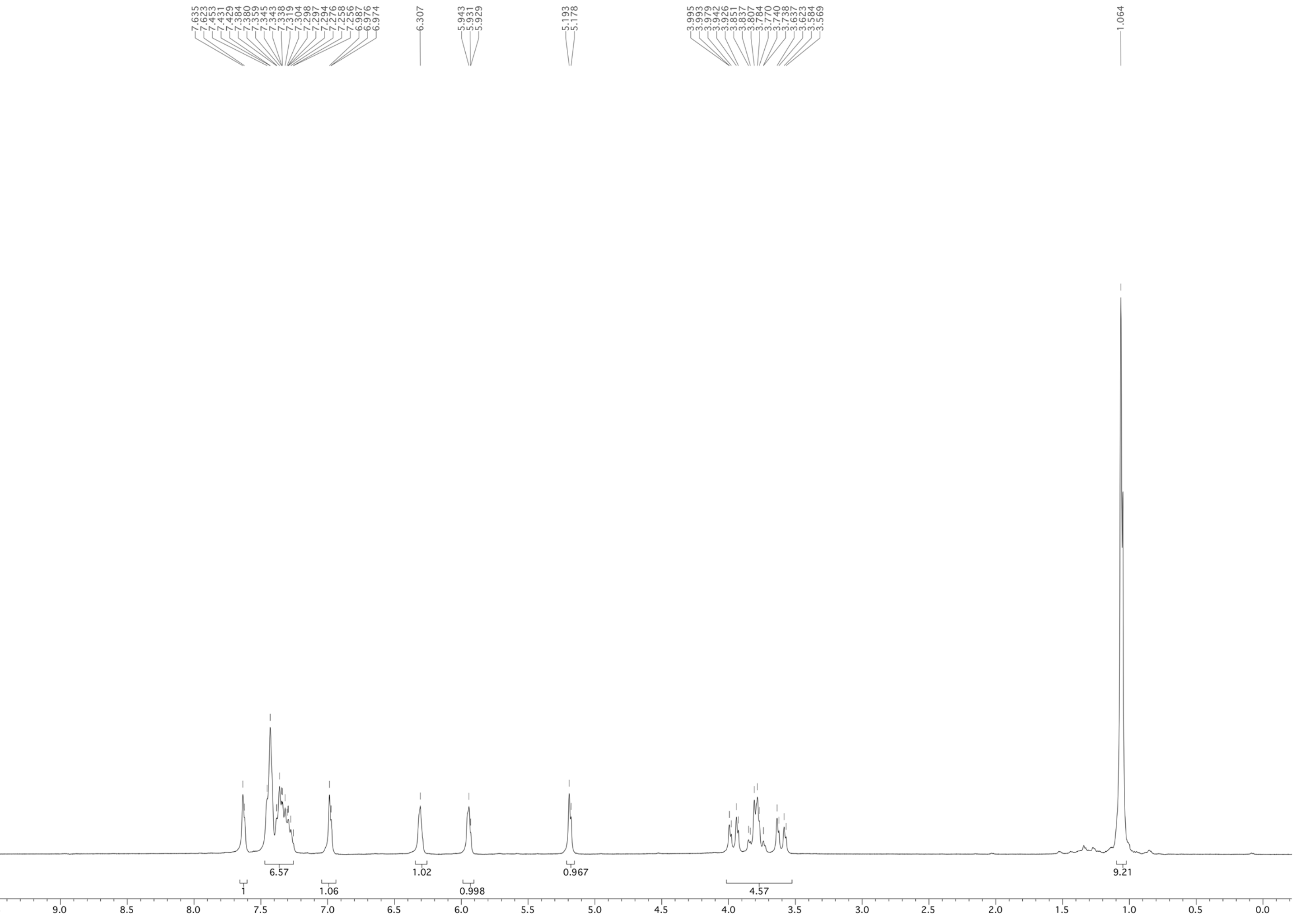

^{13}C NMR
75 MHz, $CDCl_3$

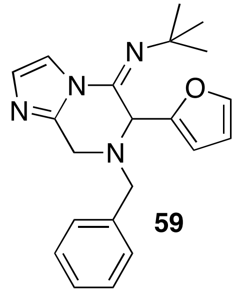

59

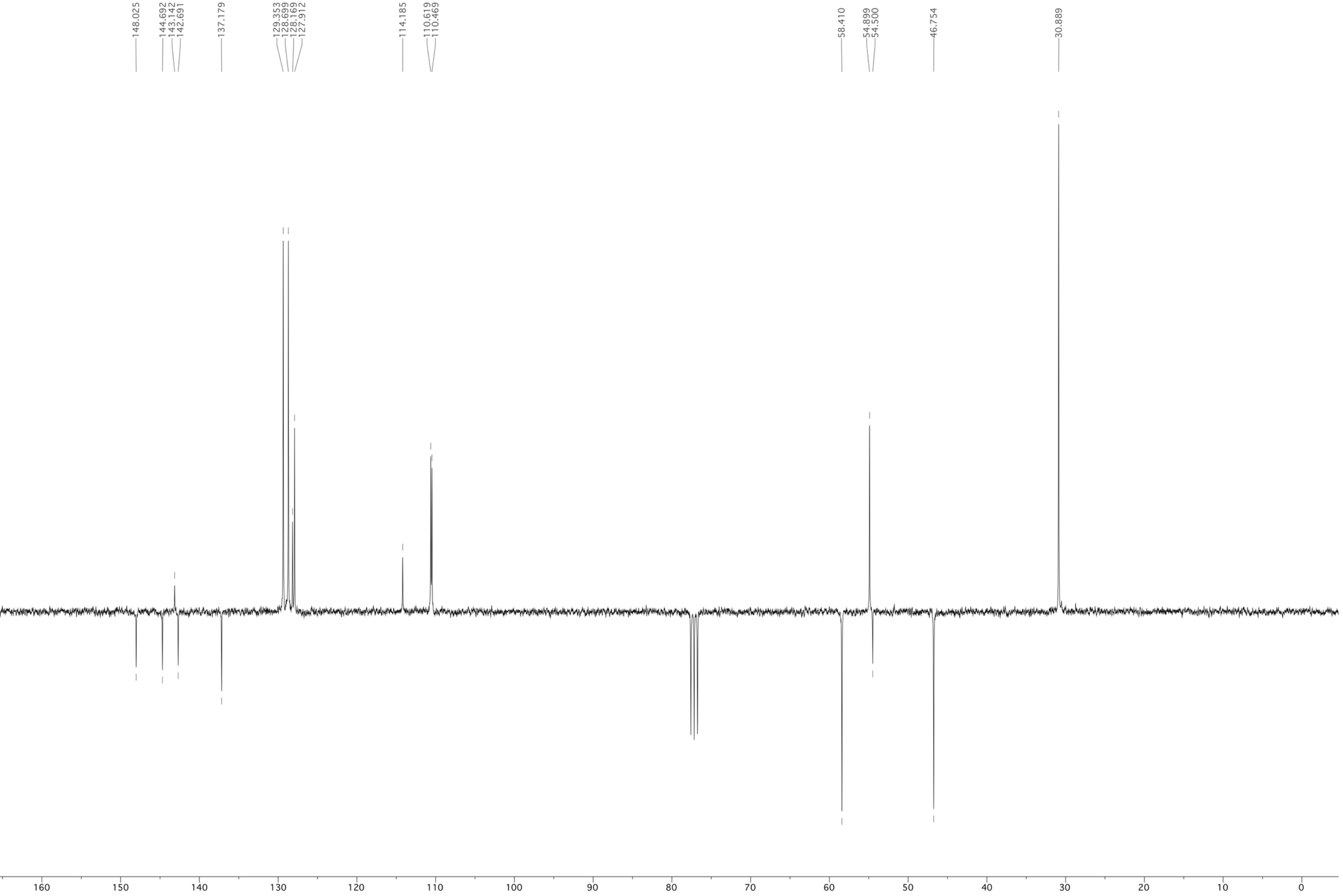

^{1}H NMR
300 MHz, $CDCl_3$

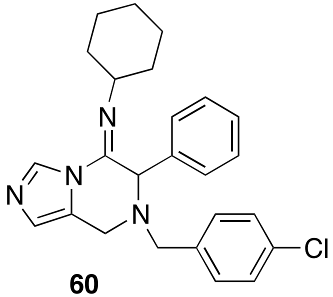

60

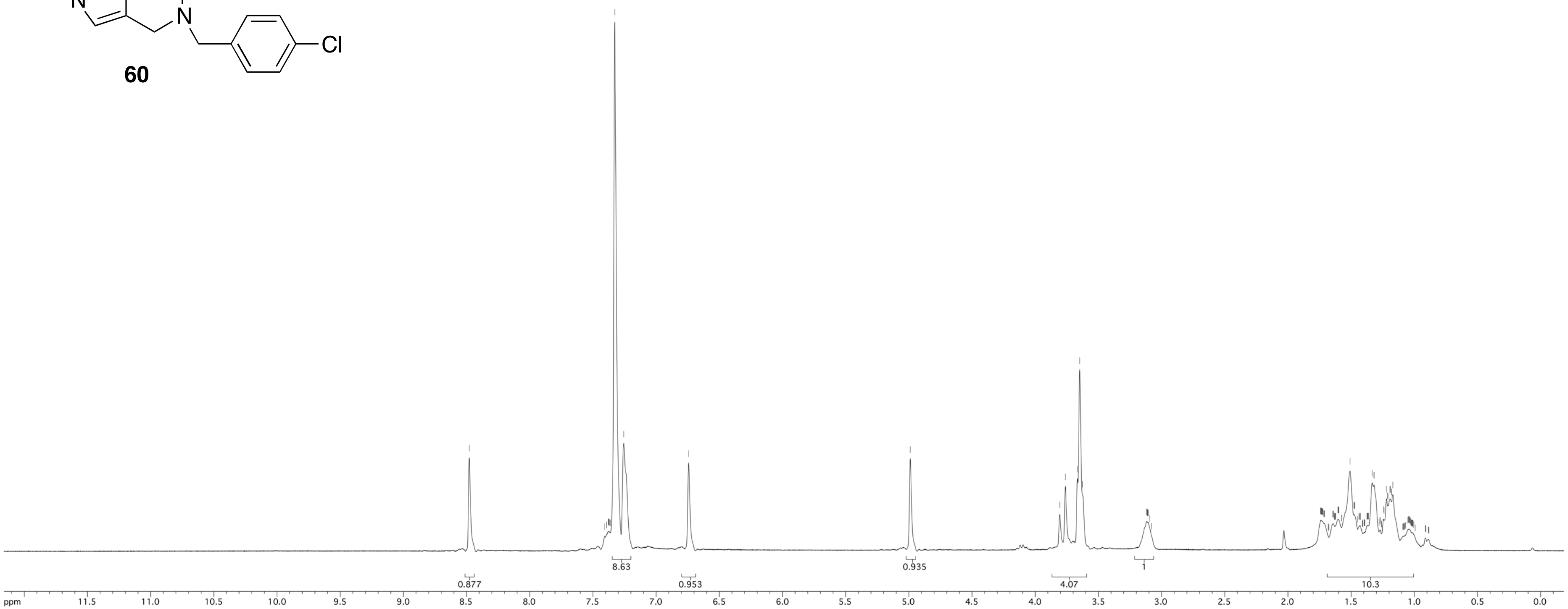

^{13}C NMR
75 MHz, $CDCl_3$

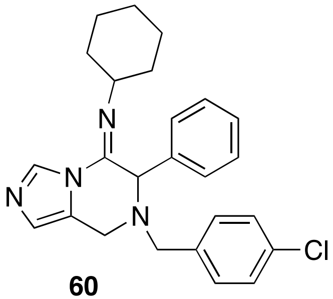

60

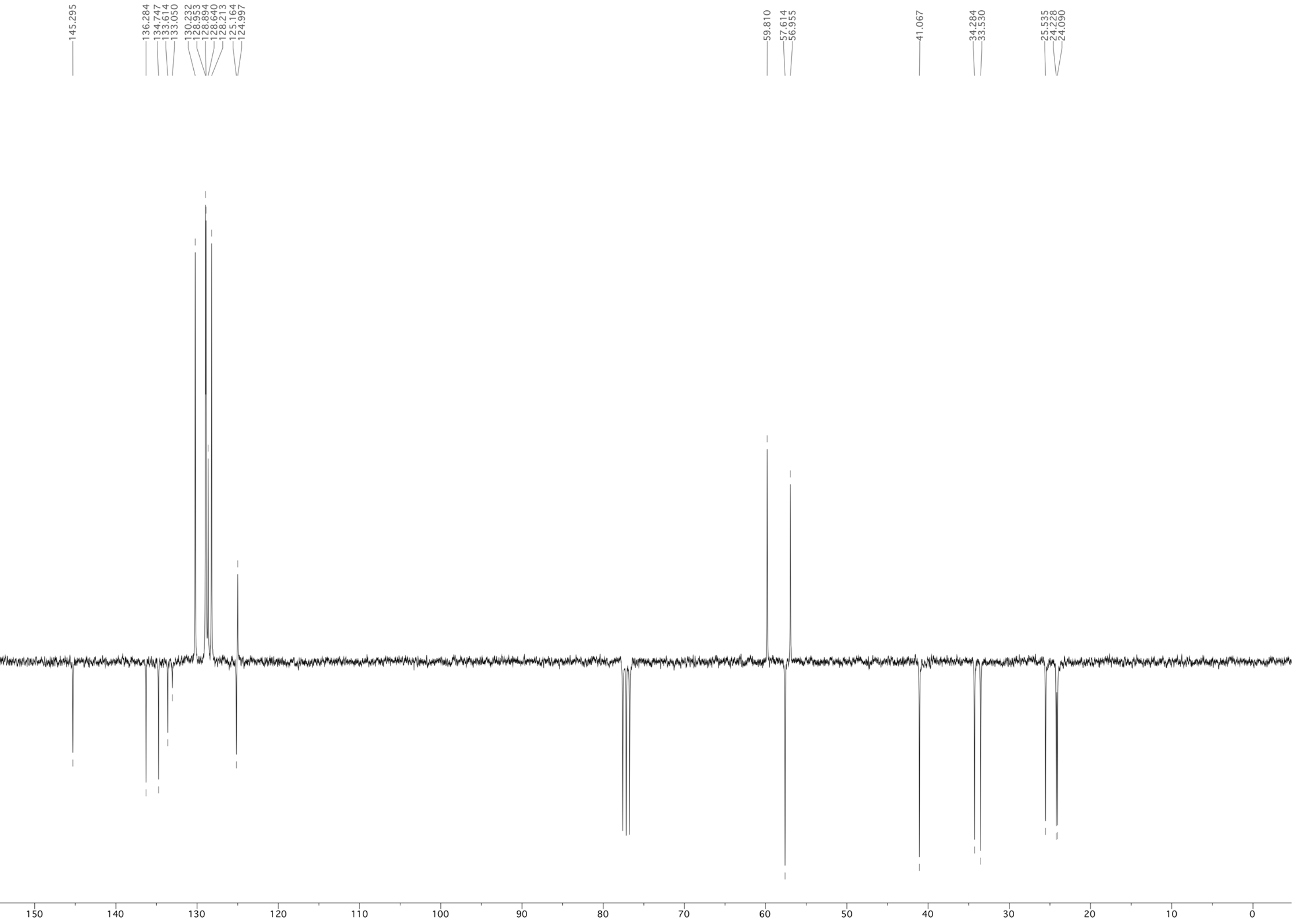

^1H NMR
300 MHz, $CDCl_3$

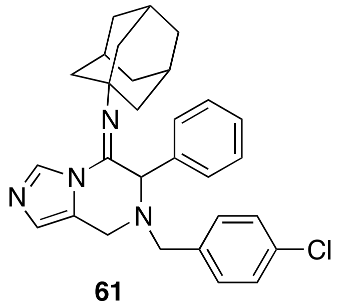

61

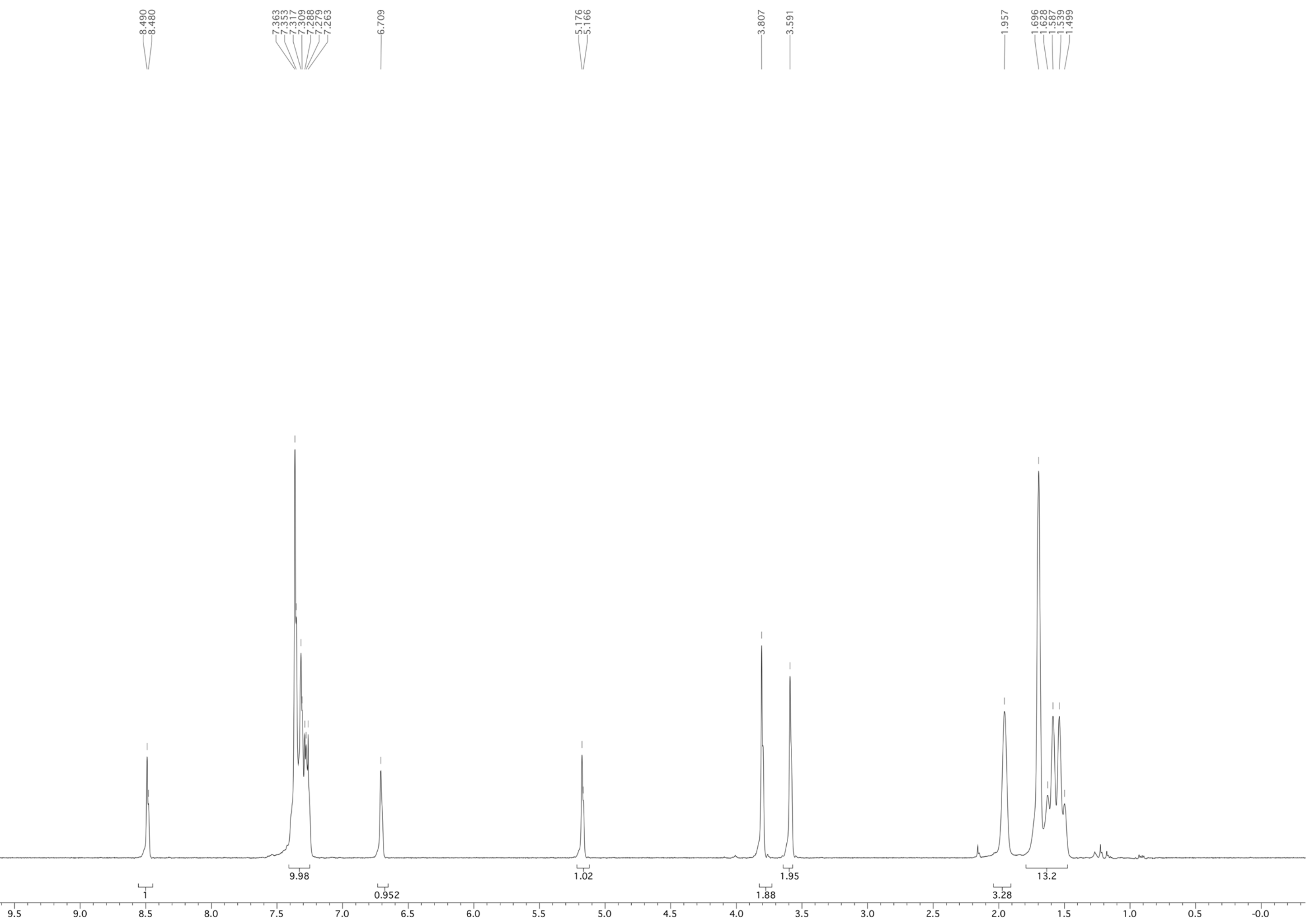

^{13}C NMR
75 MHz, $CDCl_3$

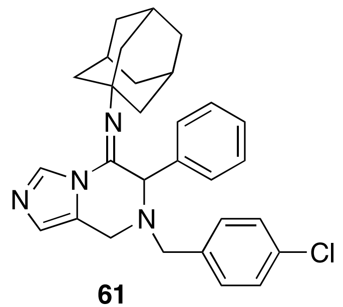

61

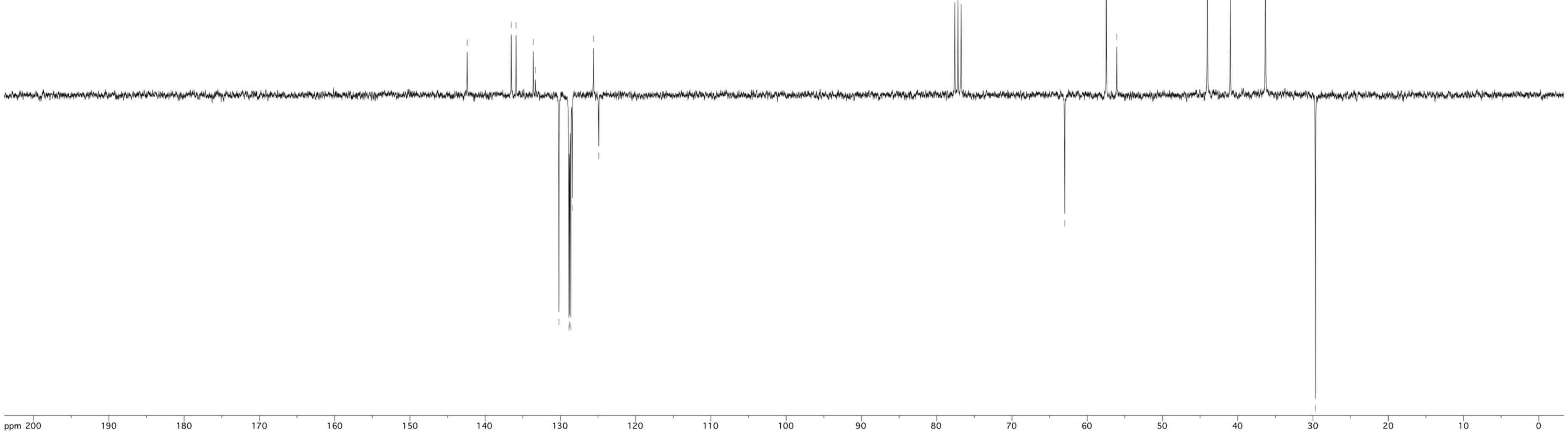

^{1}H NMR
300 MHz, $CDCl_3$

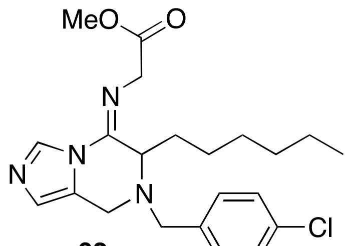

62

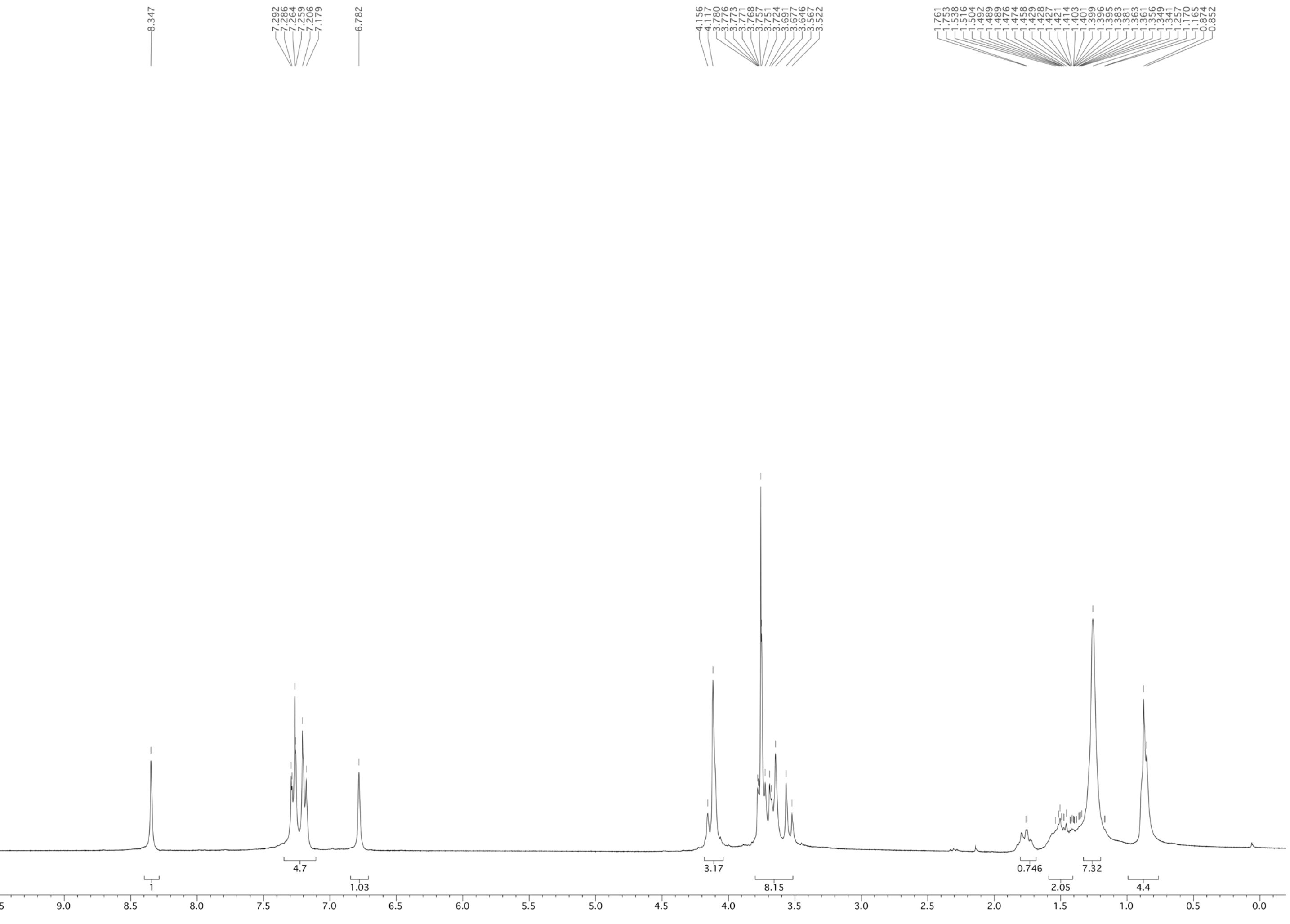

^{13}C NMR
75 MHz, $CDCl_3$

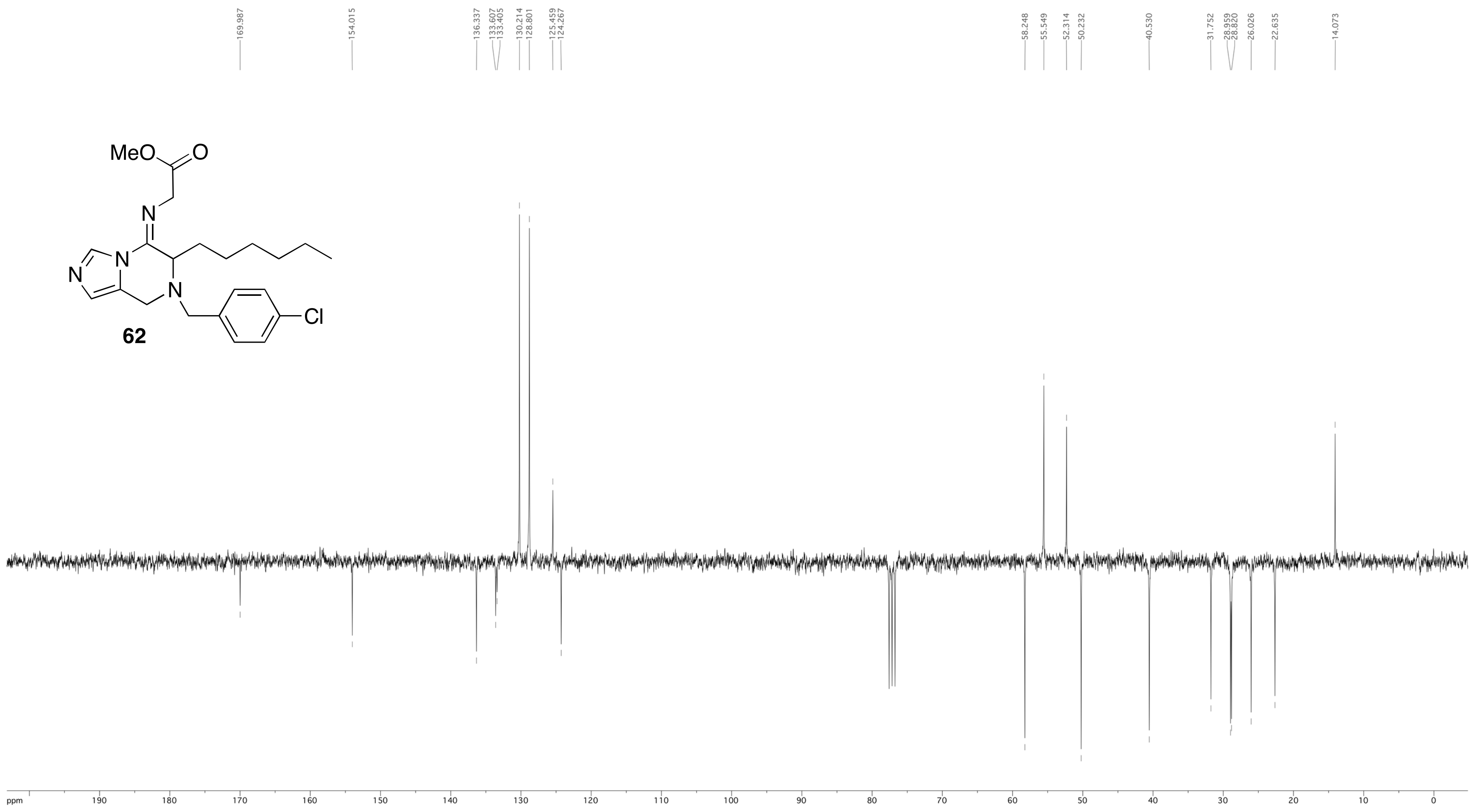

62

^{1}H NMR
300 MHz, $CDCl_3$

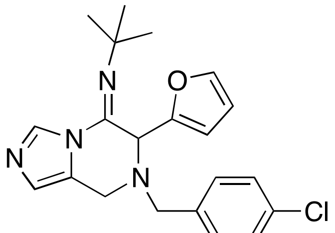

63

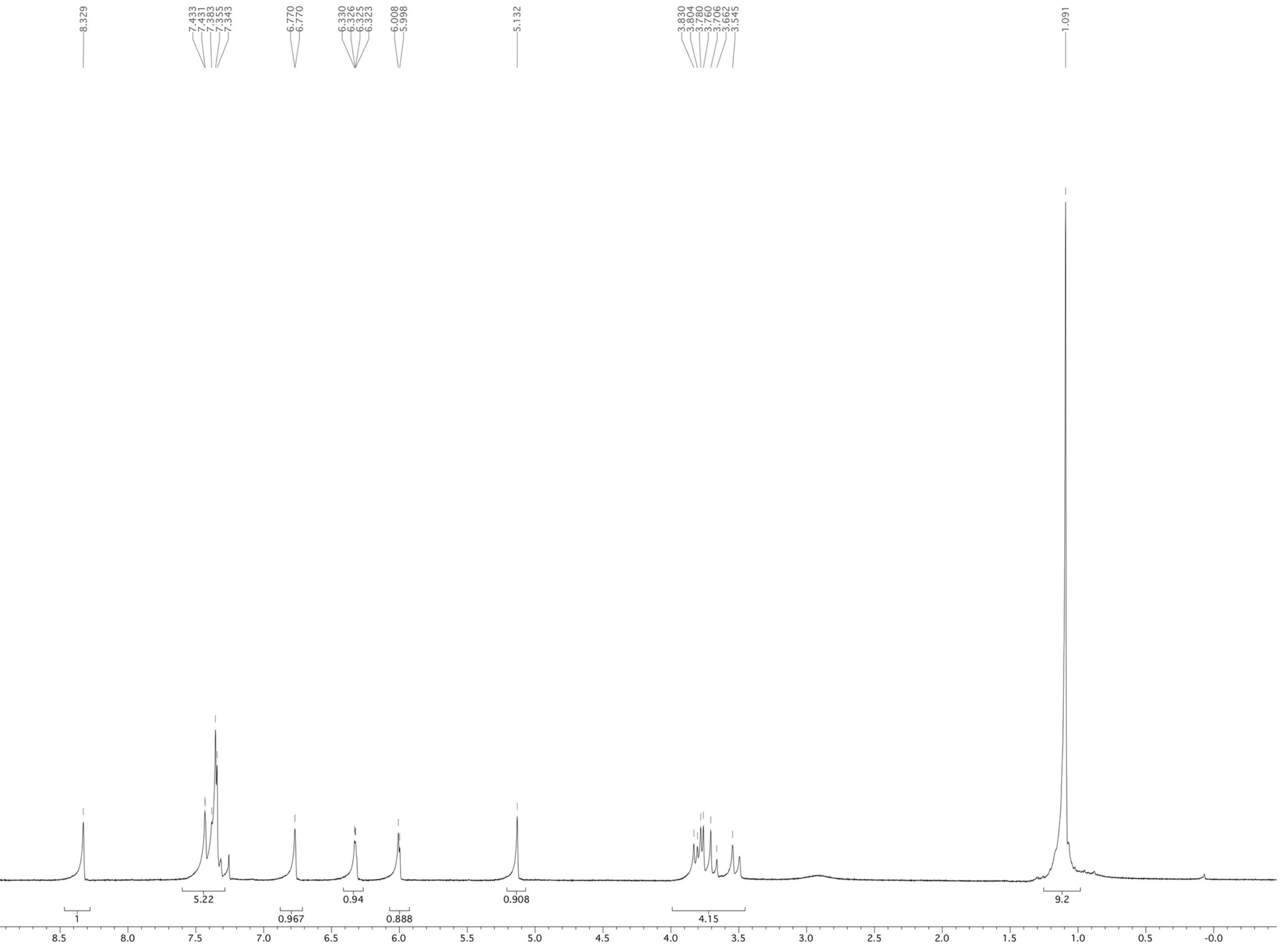

^{13}C NMR
75 MHz, $CDCl_3$

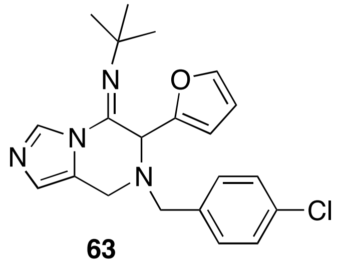

63

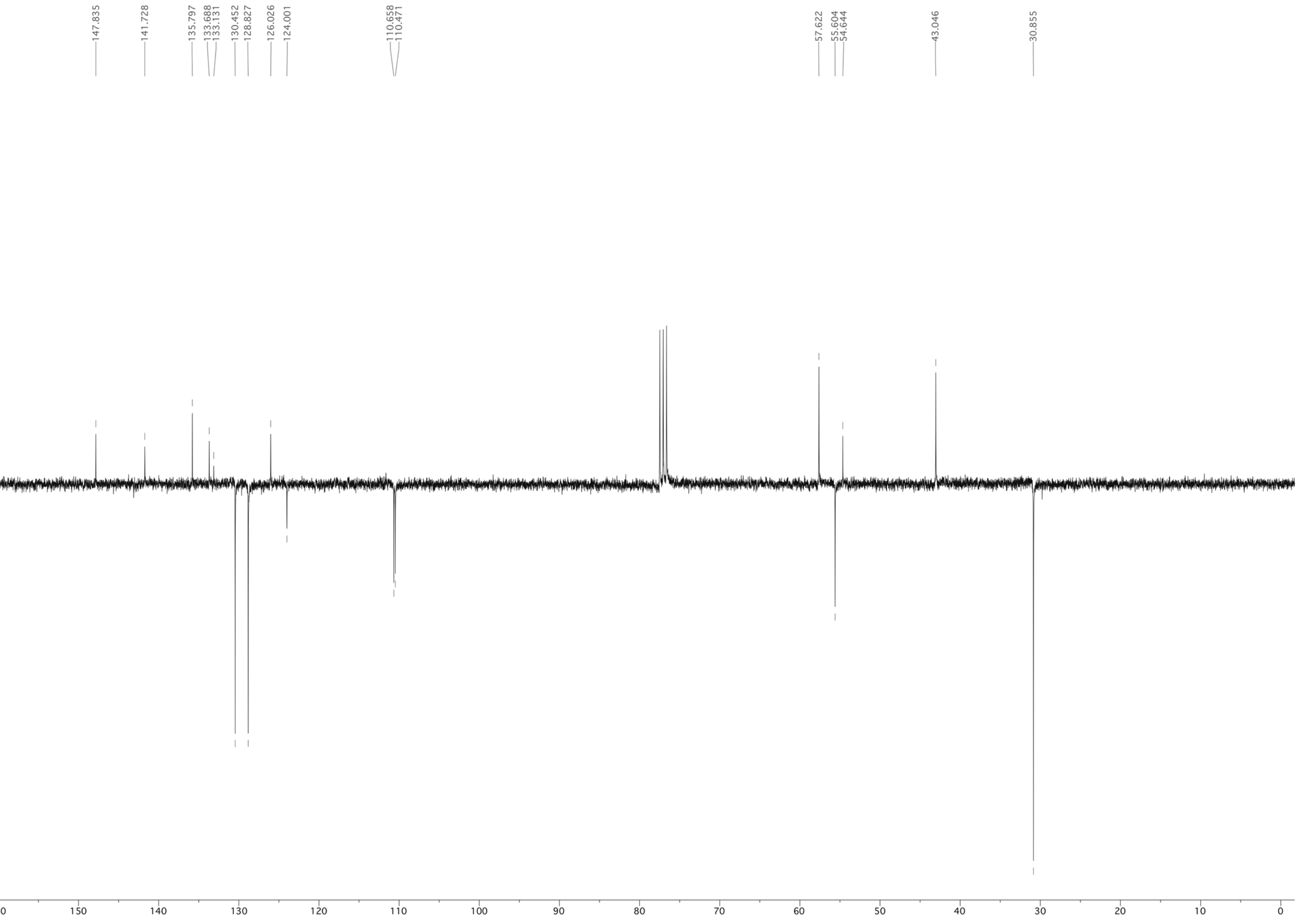

^1H NMR
300 MHz, $CDCl_3$

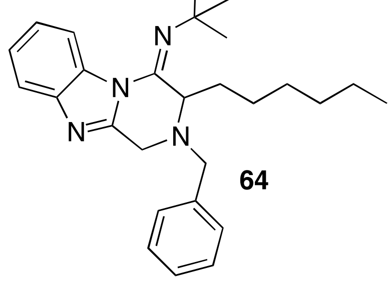

64

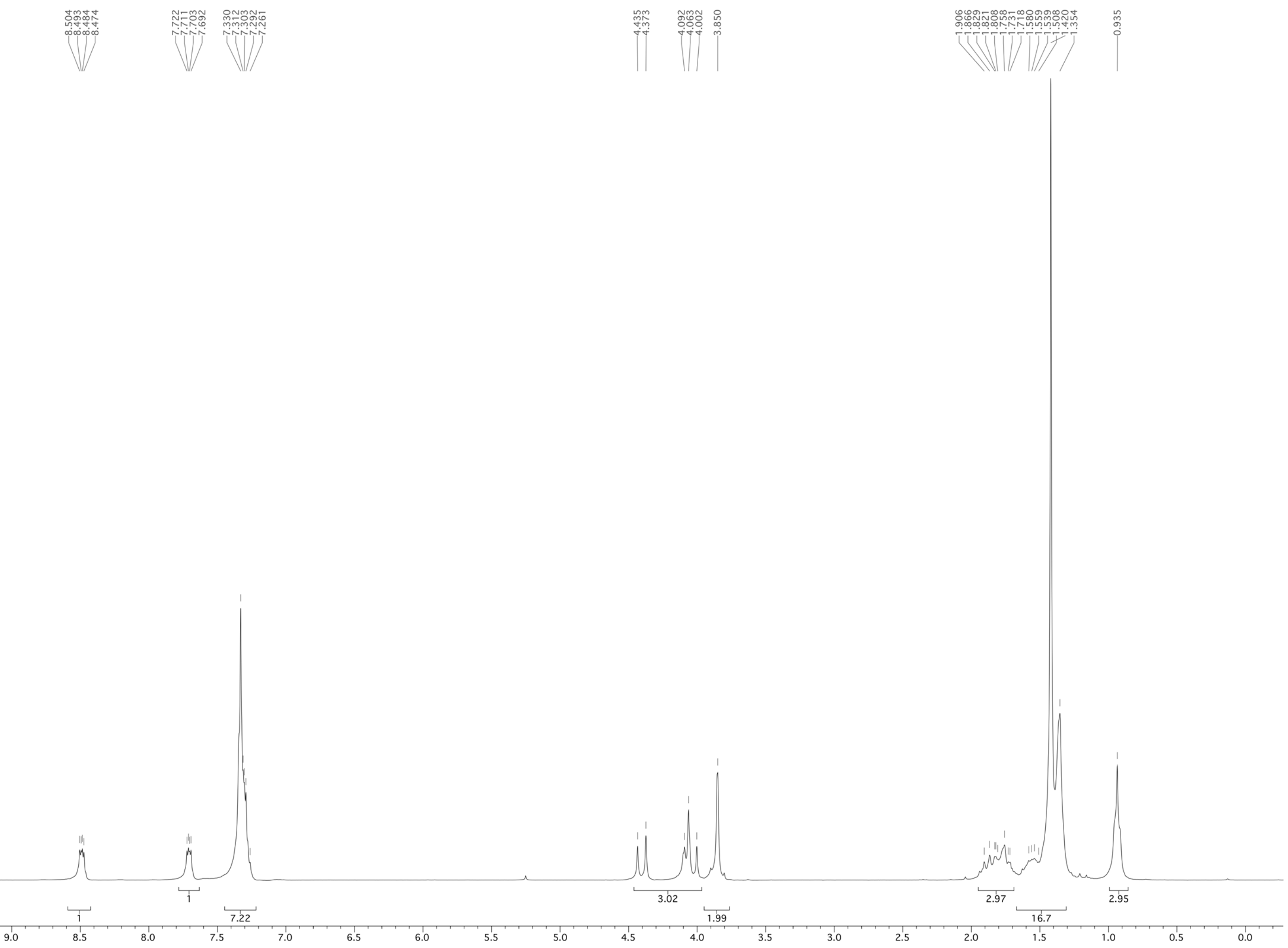

^{13}C NMR
75 MHz, $CDCl_3$

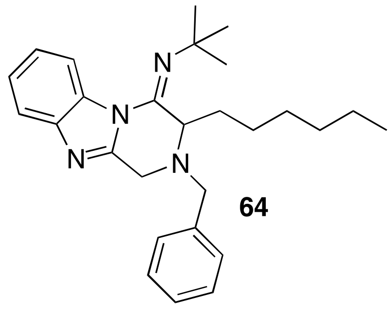

64

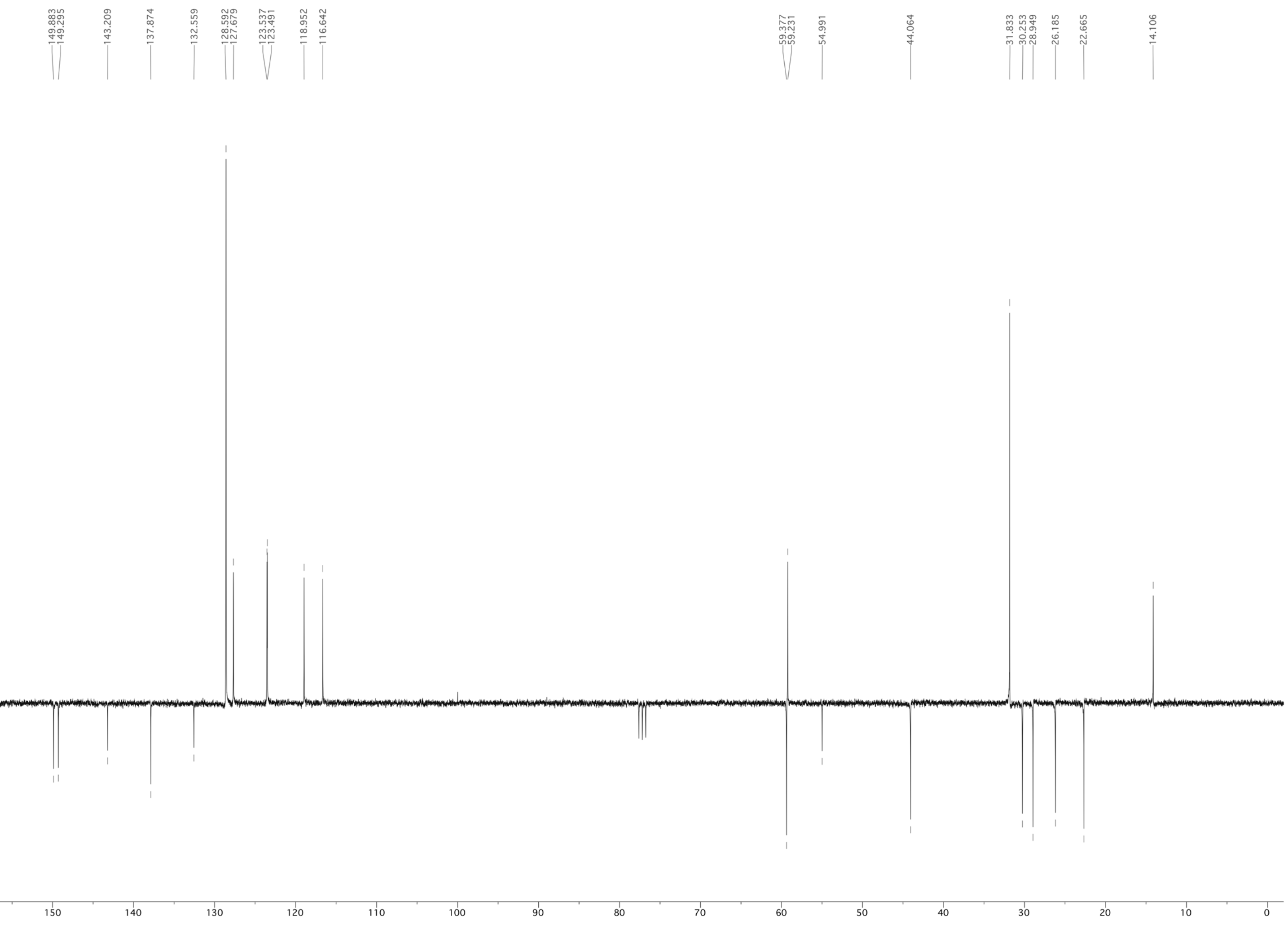

^1H NMR
300 MHz, $CDCl_3$

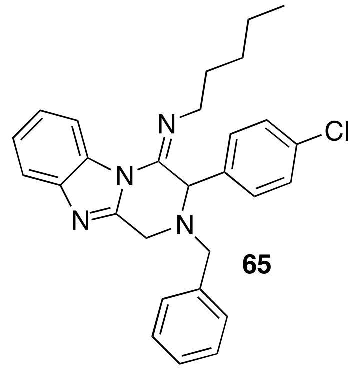

65

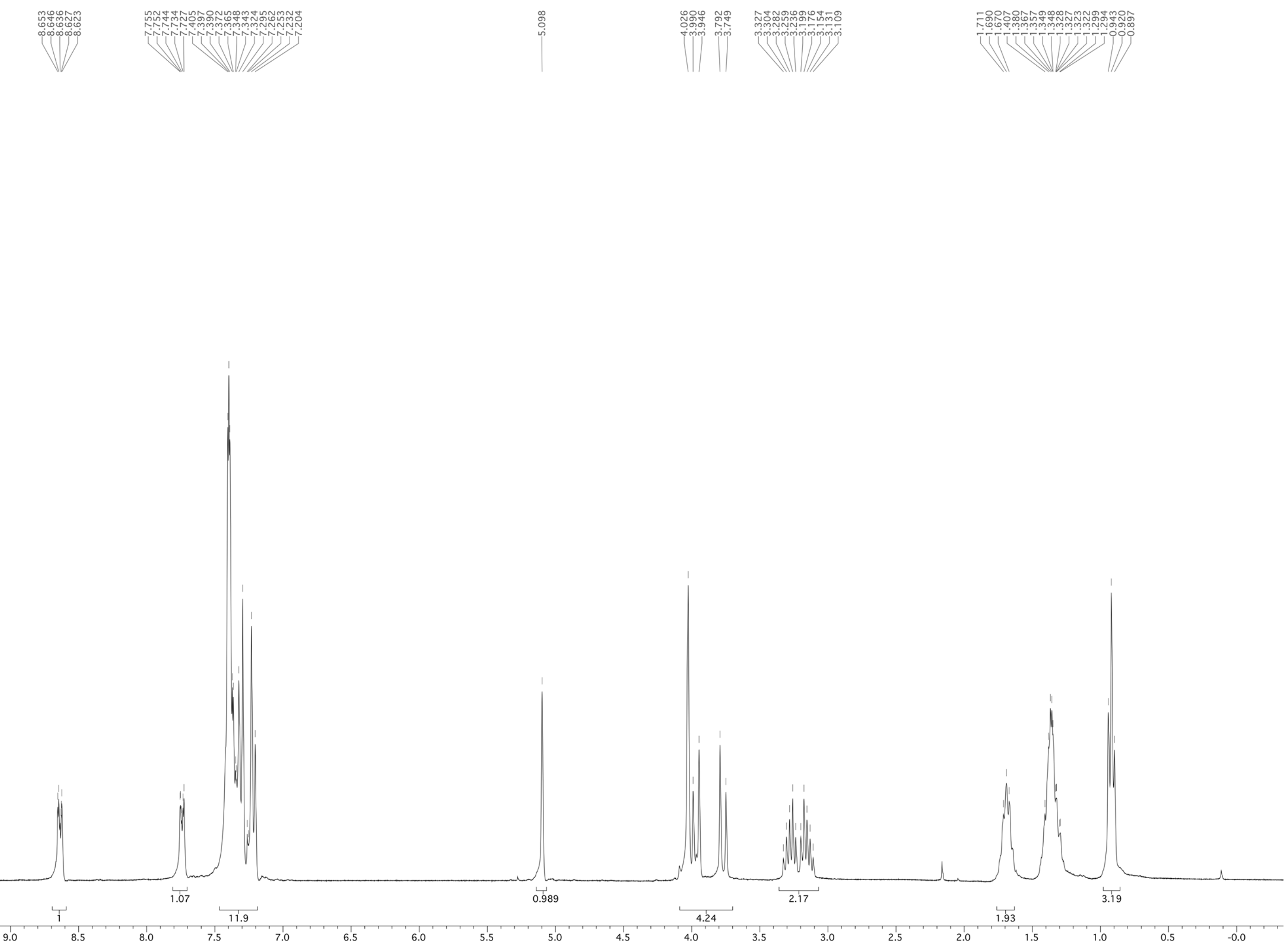

^{13}C NMR
75 MHz, $CDCl_3$

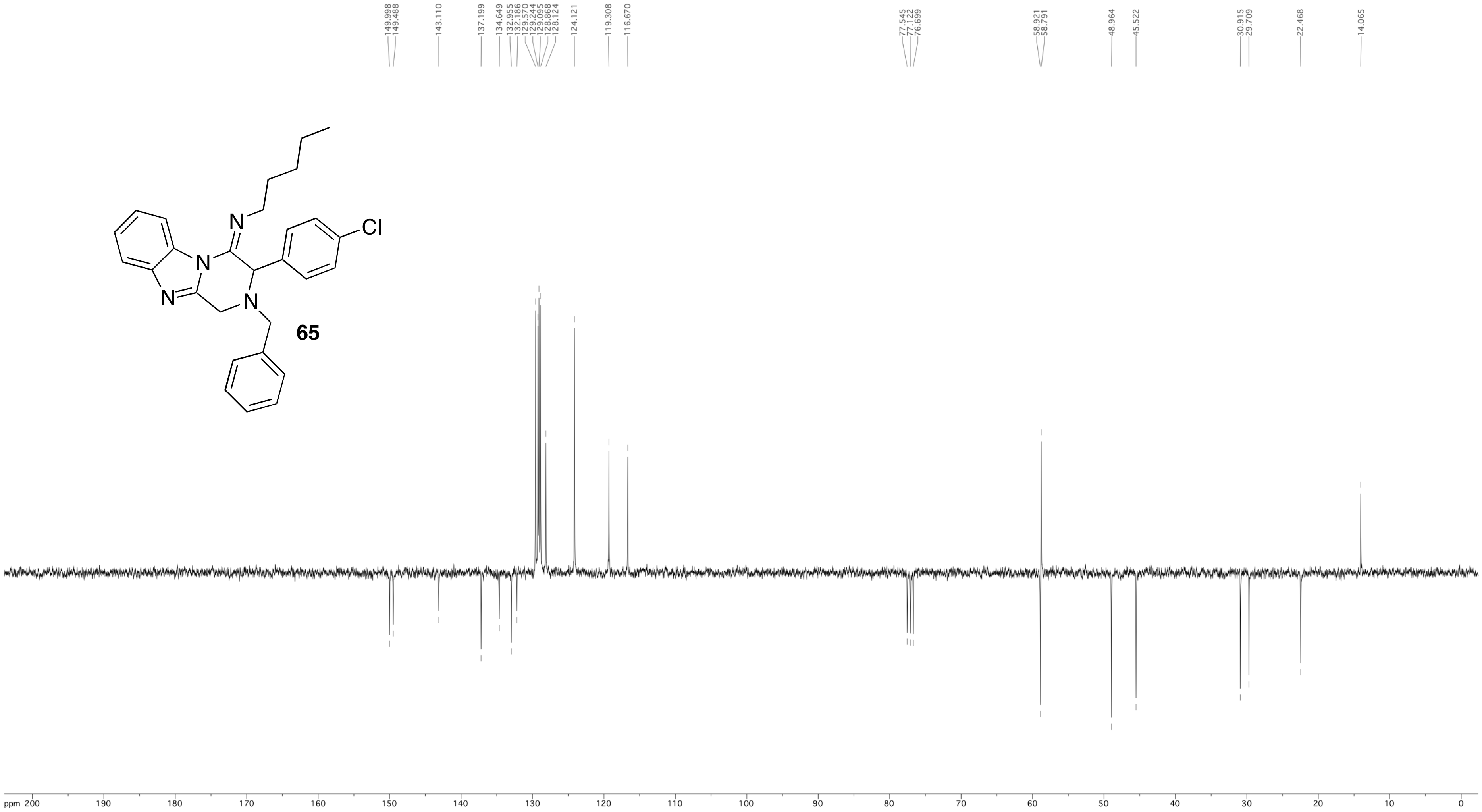

^{1}H NMR
300 MHz, $CDCl_3$

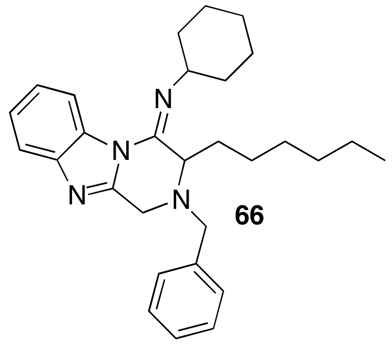

66

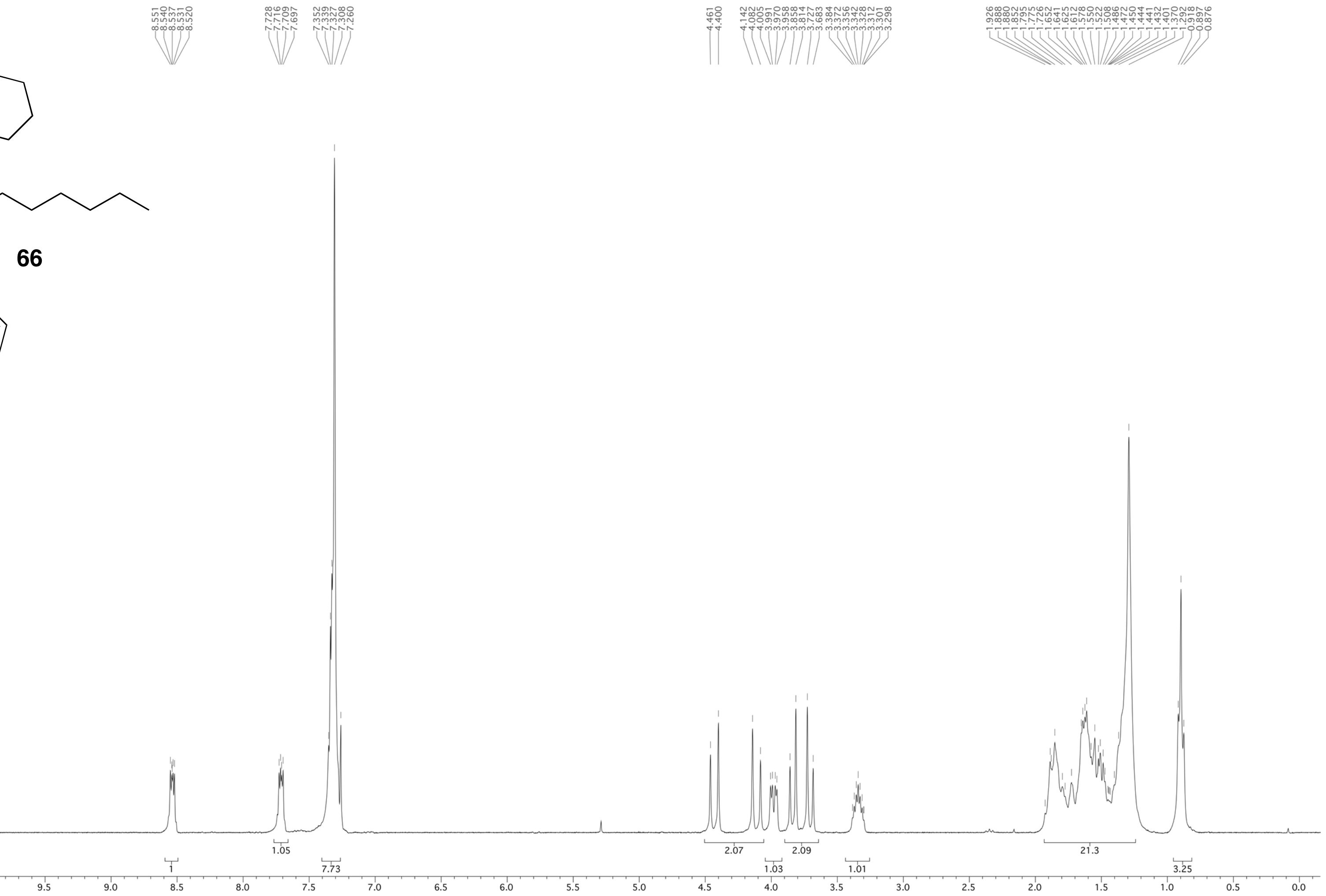

^{13}C NMR
75 MHz, $CDCl_3$

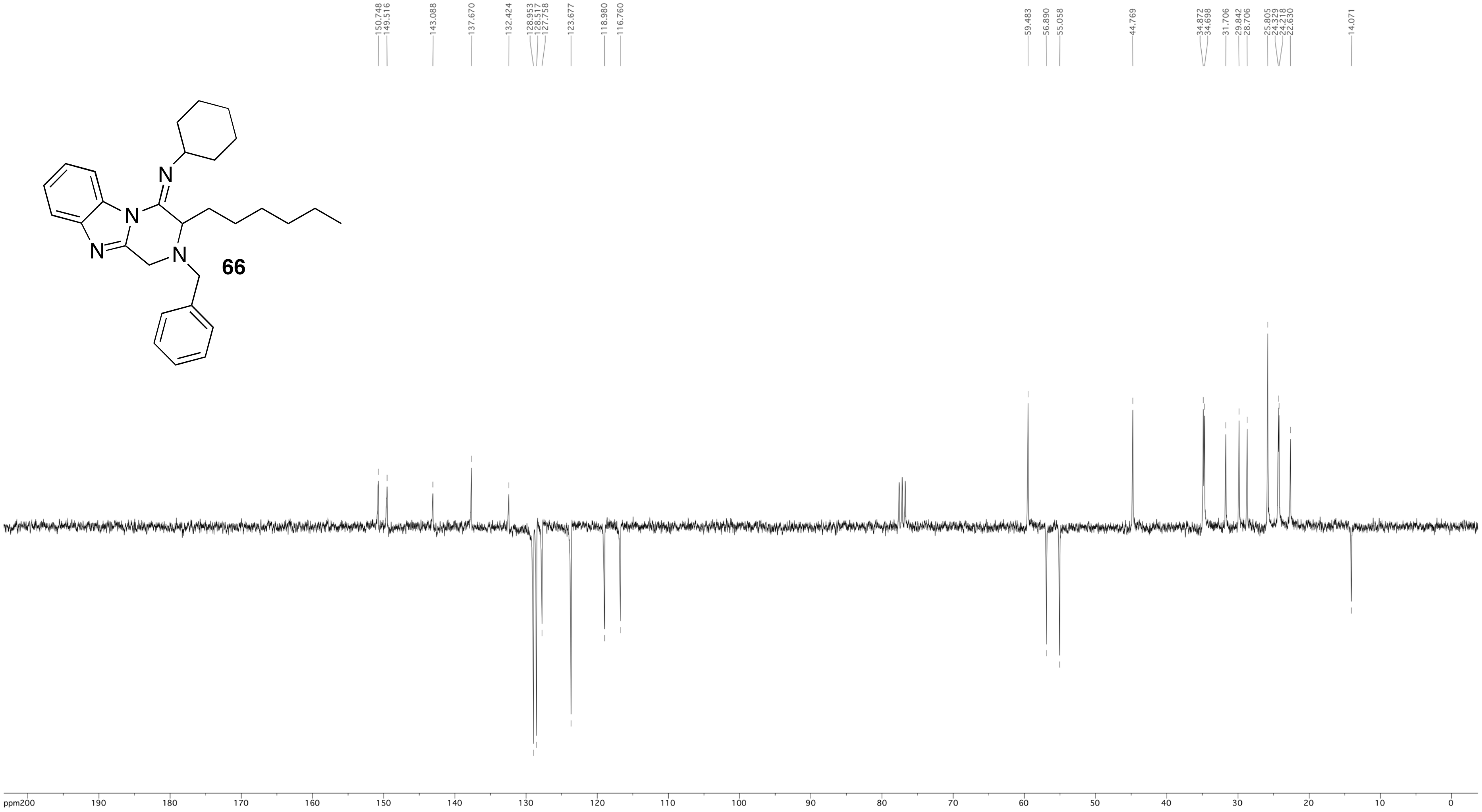

66

^{1}H NMR
300 MHz, $CDCl_3$

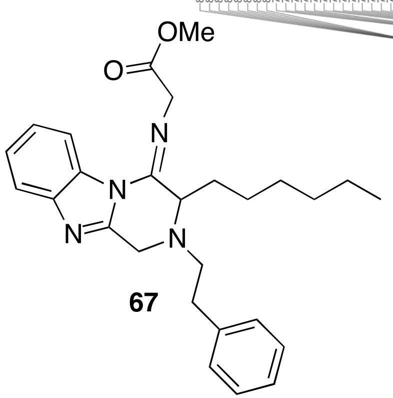

67

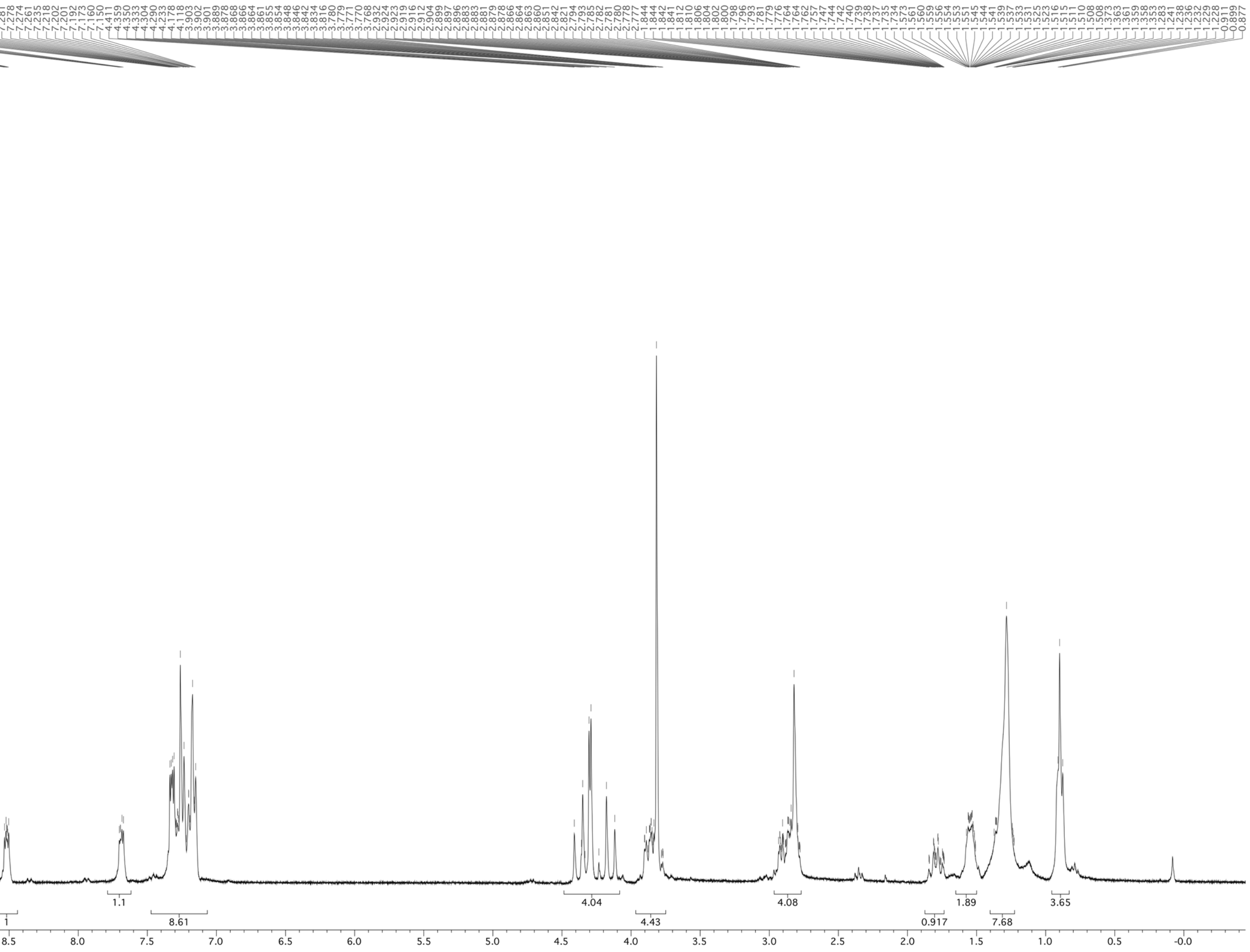

^{13}C NMR
75 MHz, $CDCl_3$

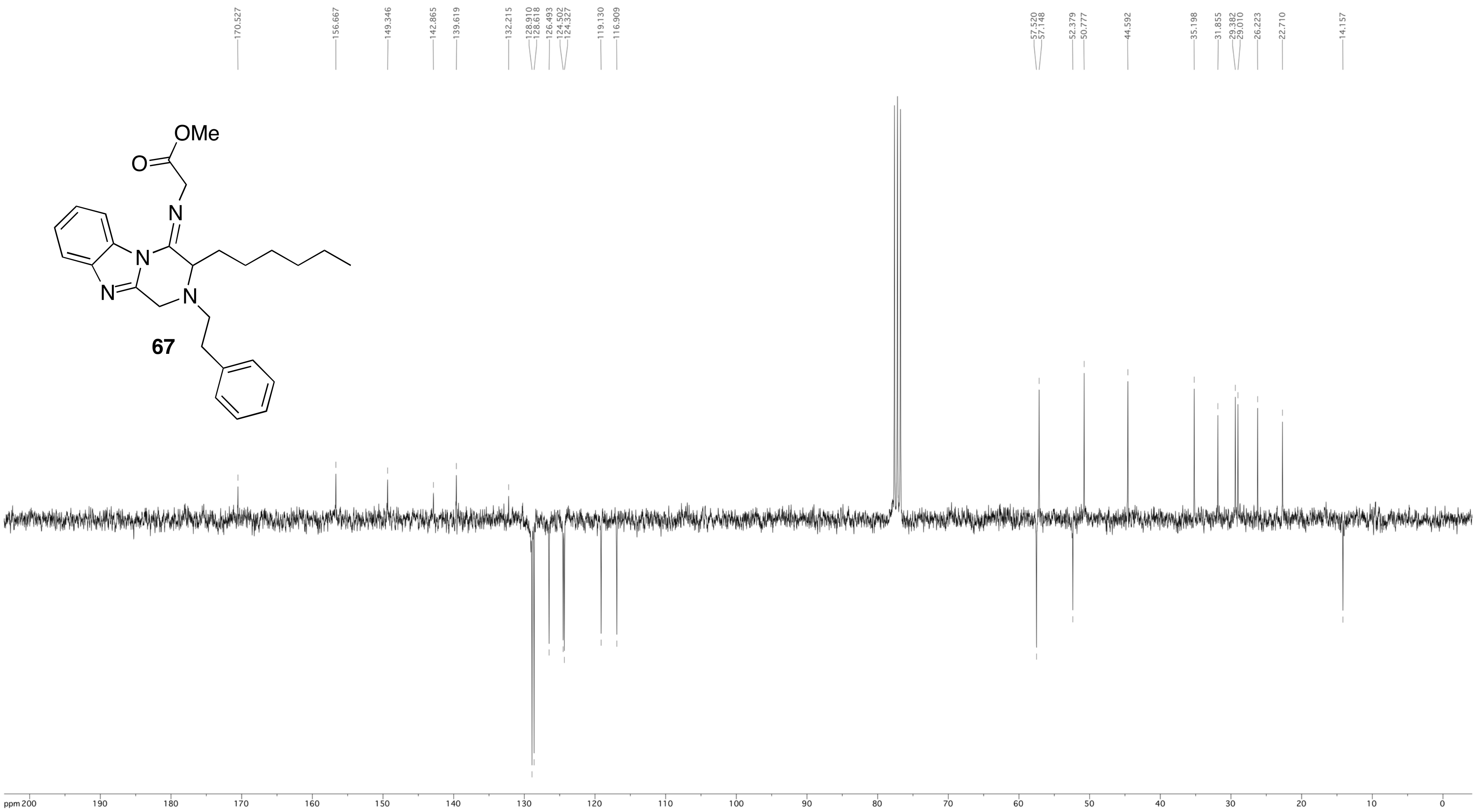

^1H NMR
400 MHz, $CDCl_3$

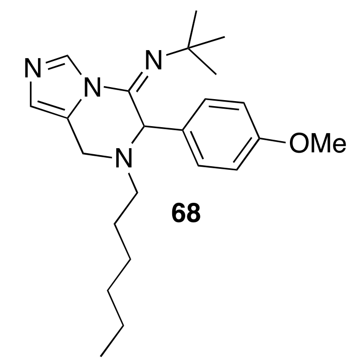

68

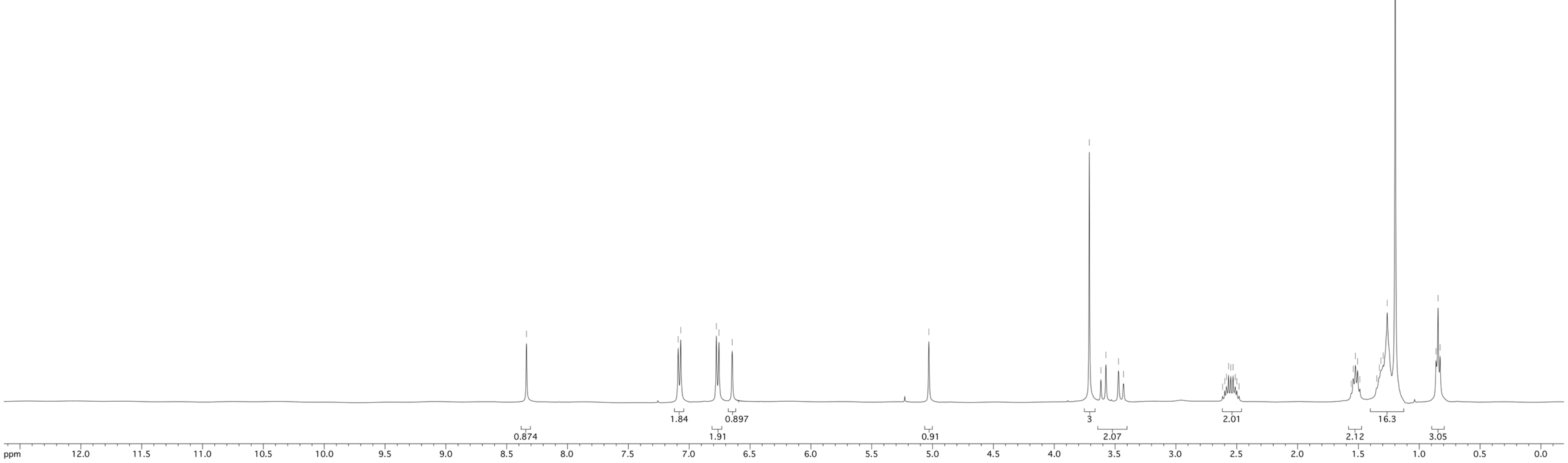

^{13}C NMR
100 MHz, $CDCl_3$

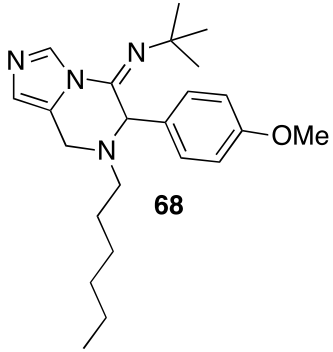

68

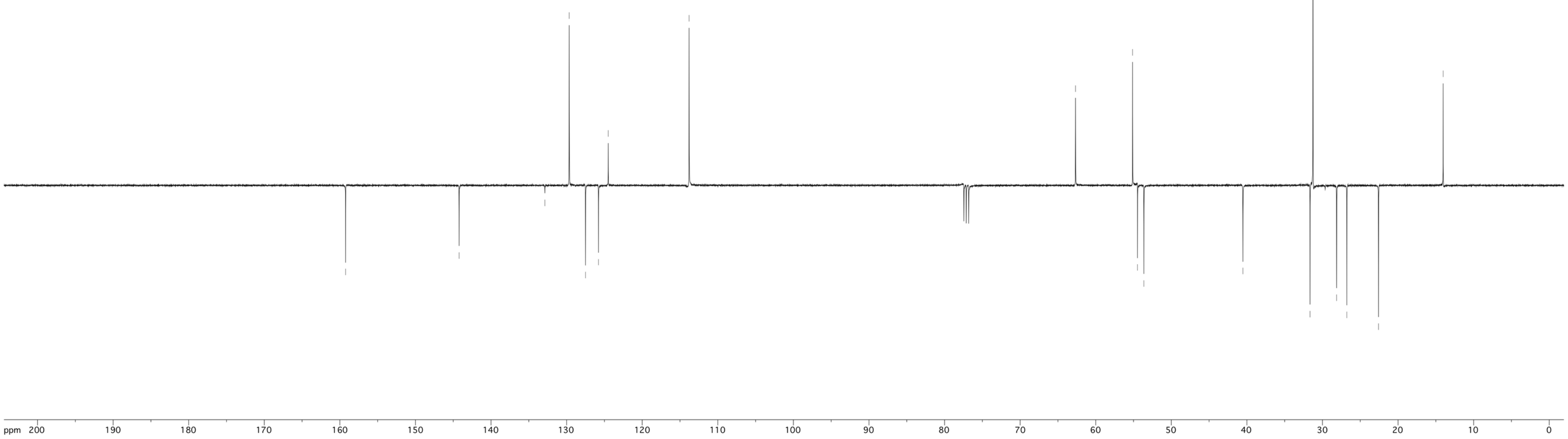

^{1}H NMR
400 MHz, $CDCl_3$

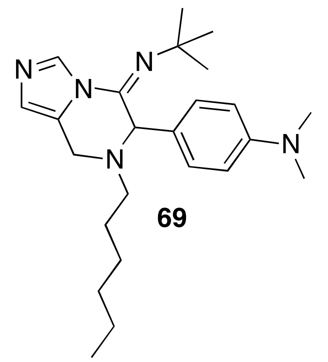

69

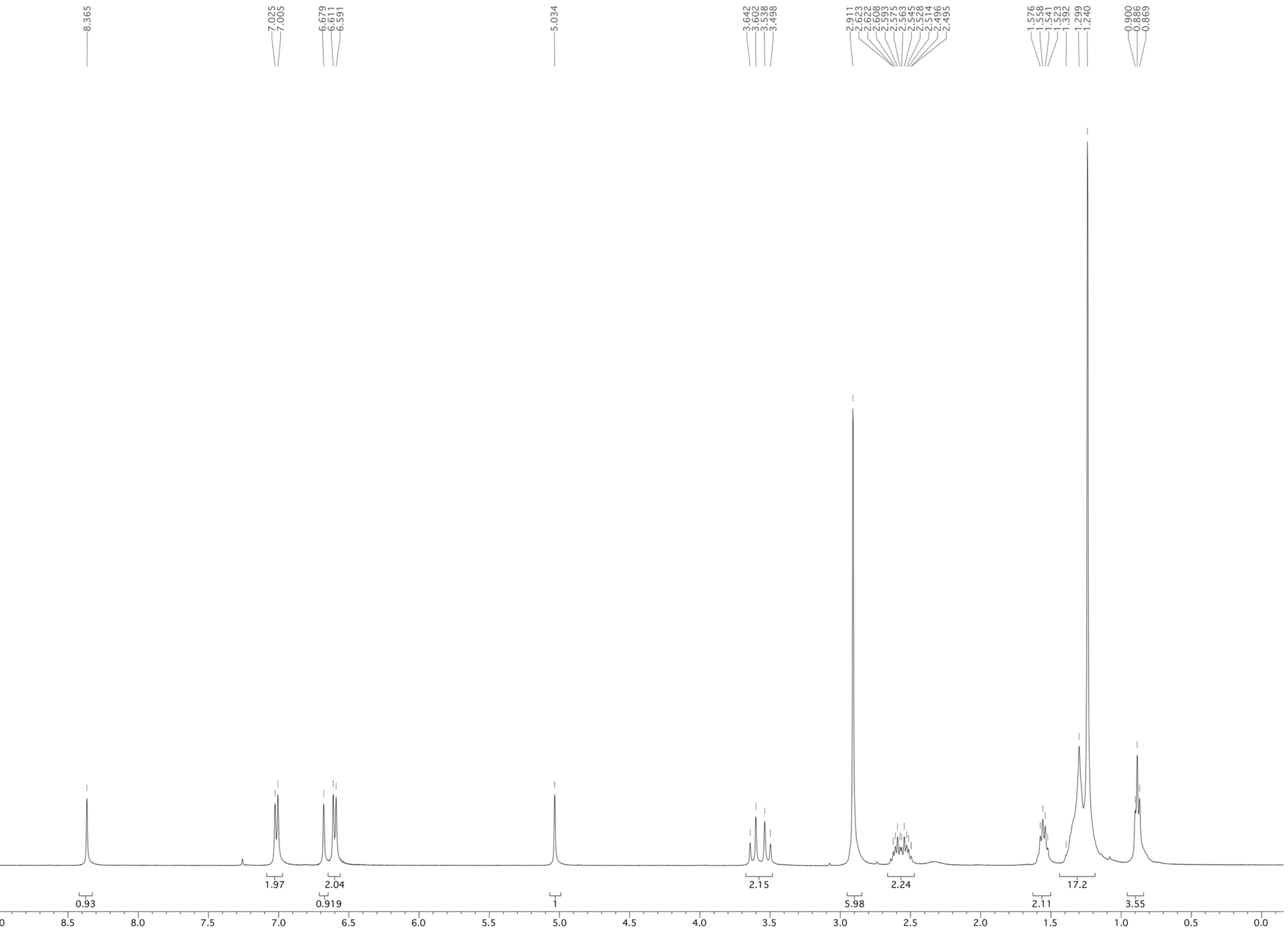

^{13}C NMR
100 MHz, $CDCl_3$

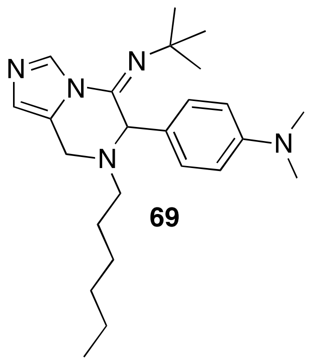

69

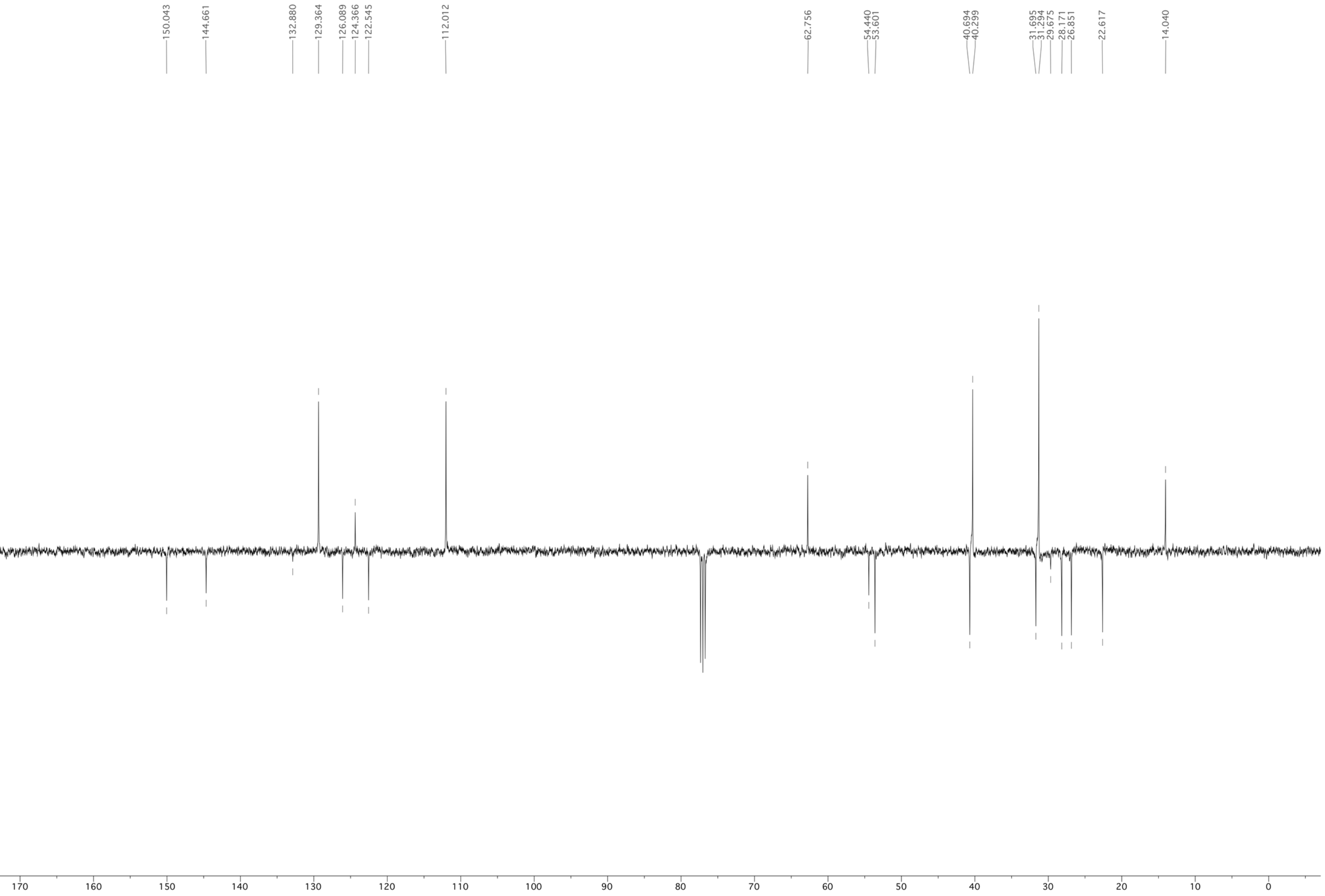

^{1}H NMR
400 MHz, $CDCl_3$

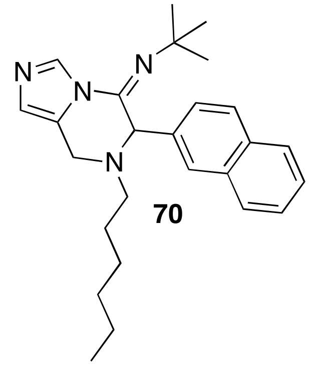

70

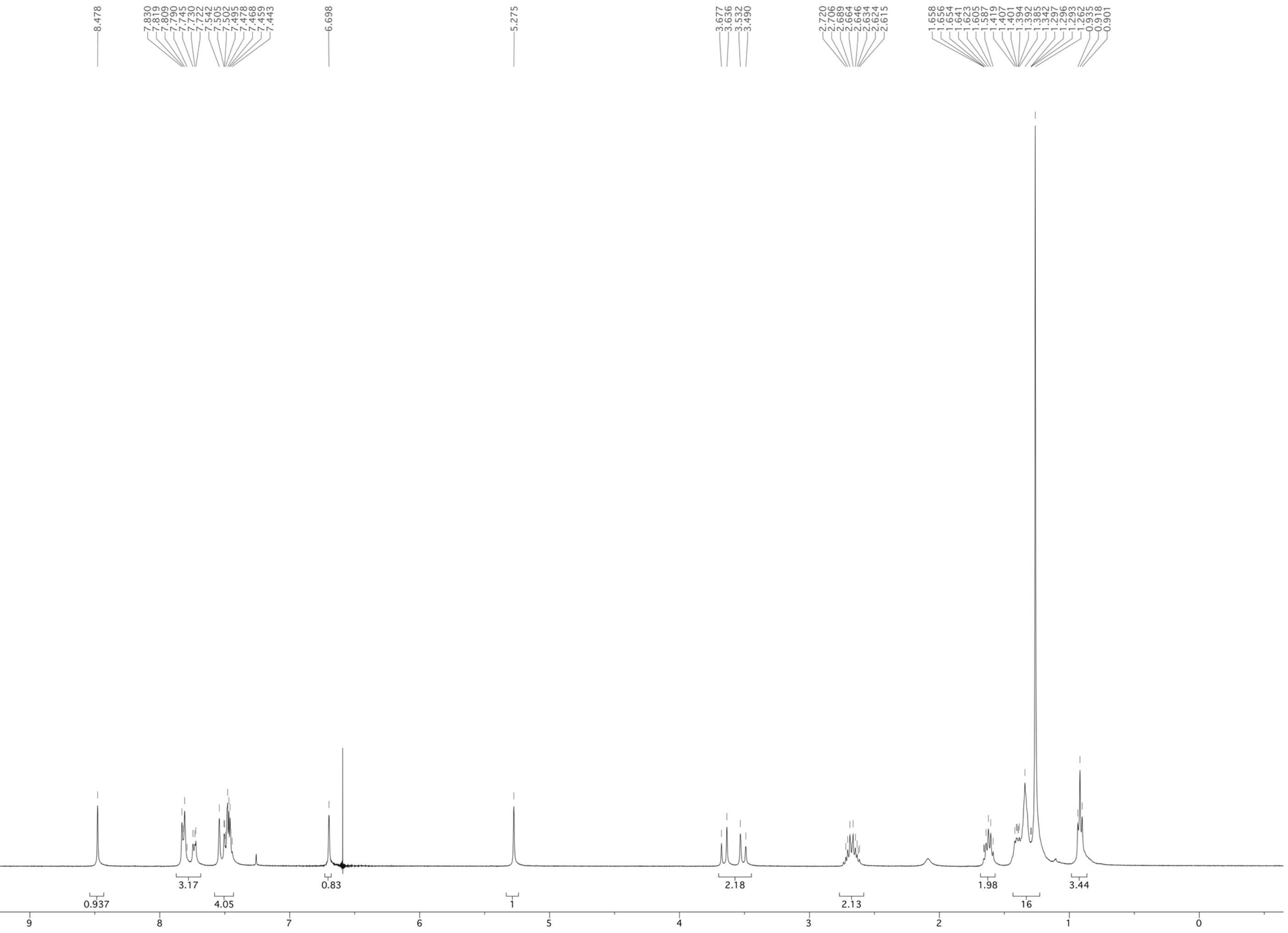

^{13}C NMR
100 MHz, $CDCl_3$

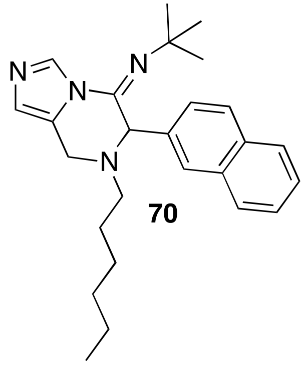

70

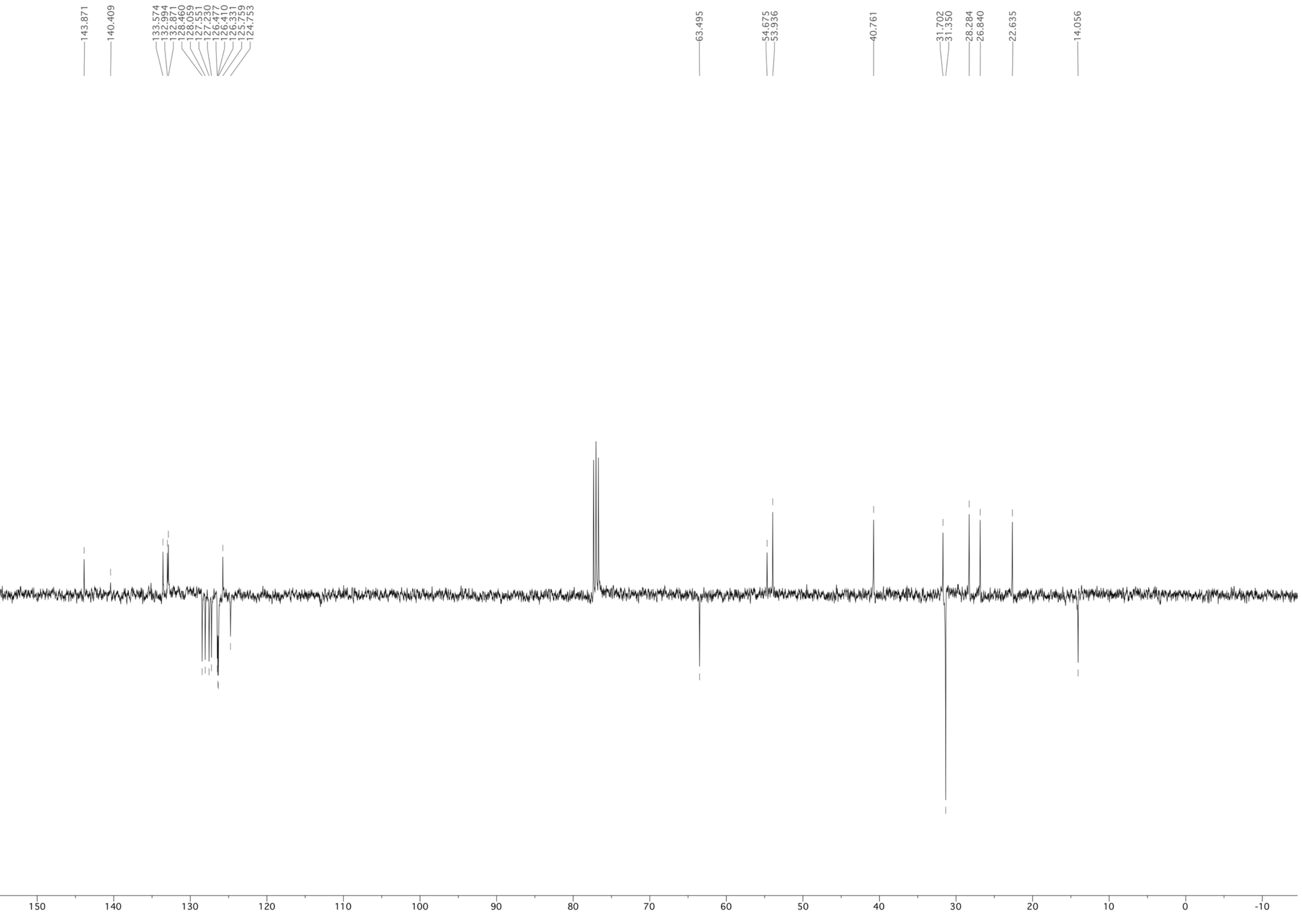

^1H NMR
400 MHz, $CDCl_3$

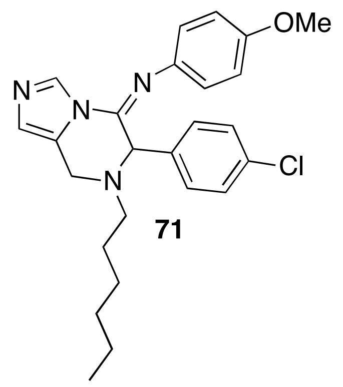

71

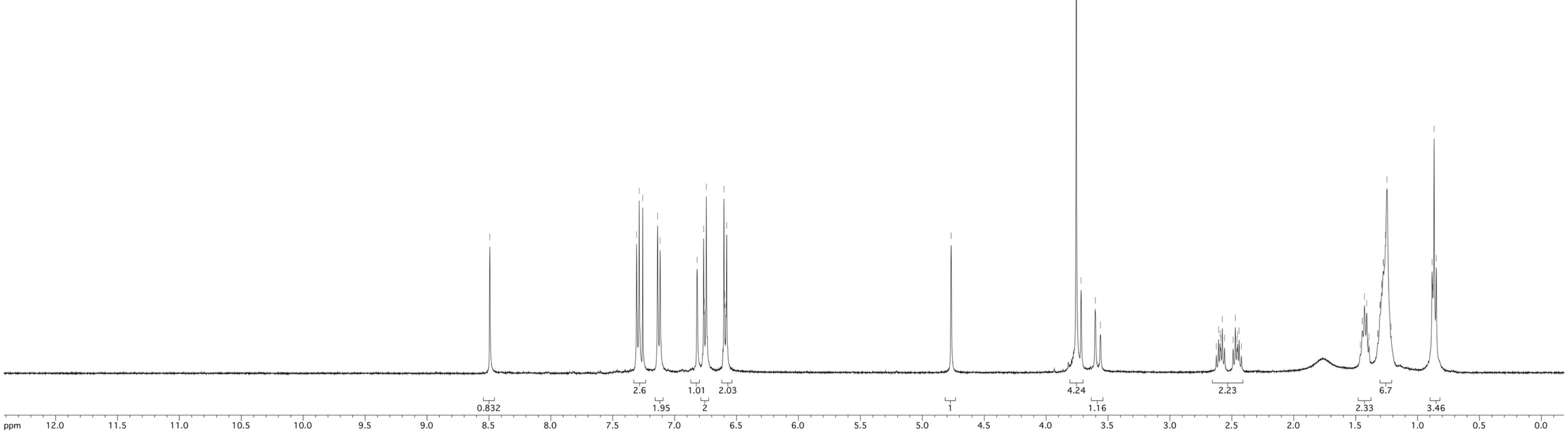

^{13}C NMR
100 MHz, $CDCl_3$

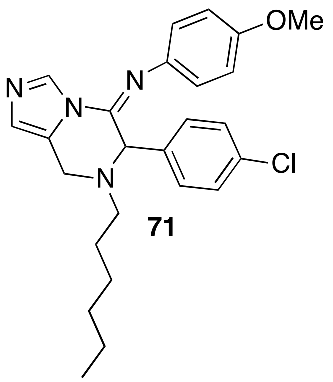

71

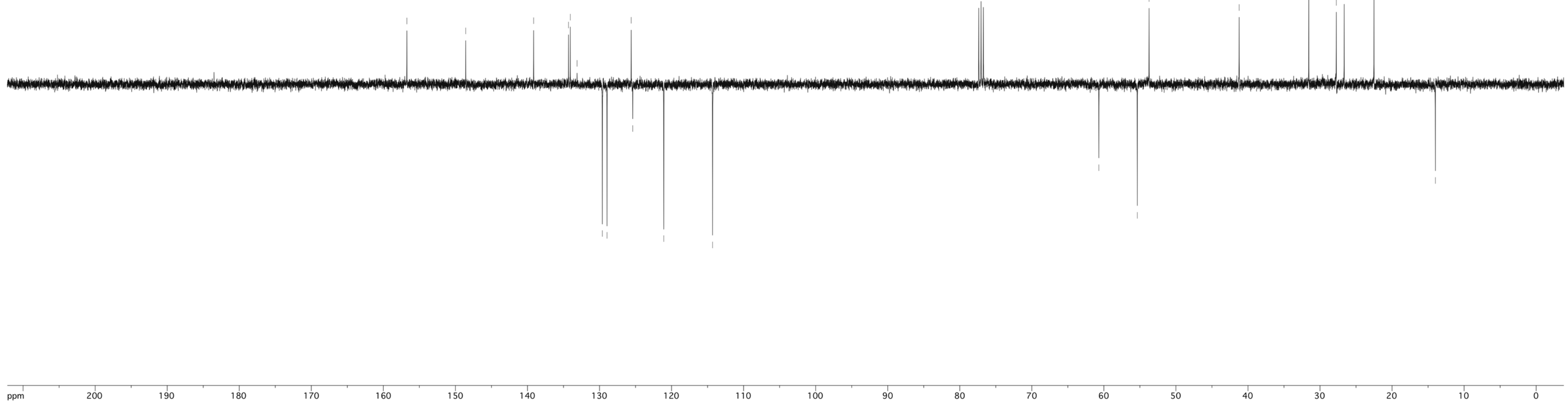

^{1}H NMR
400 MHz, $CDCl_3$

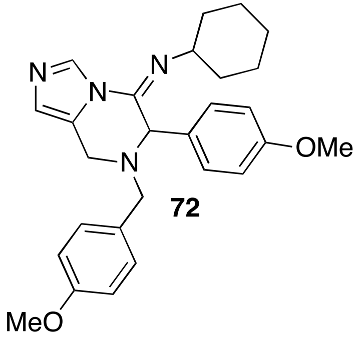

72

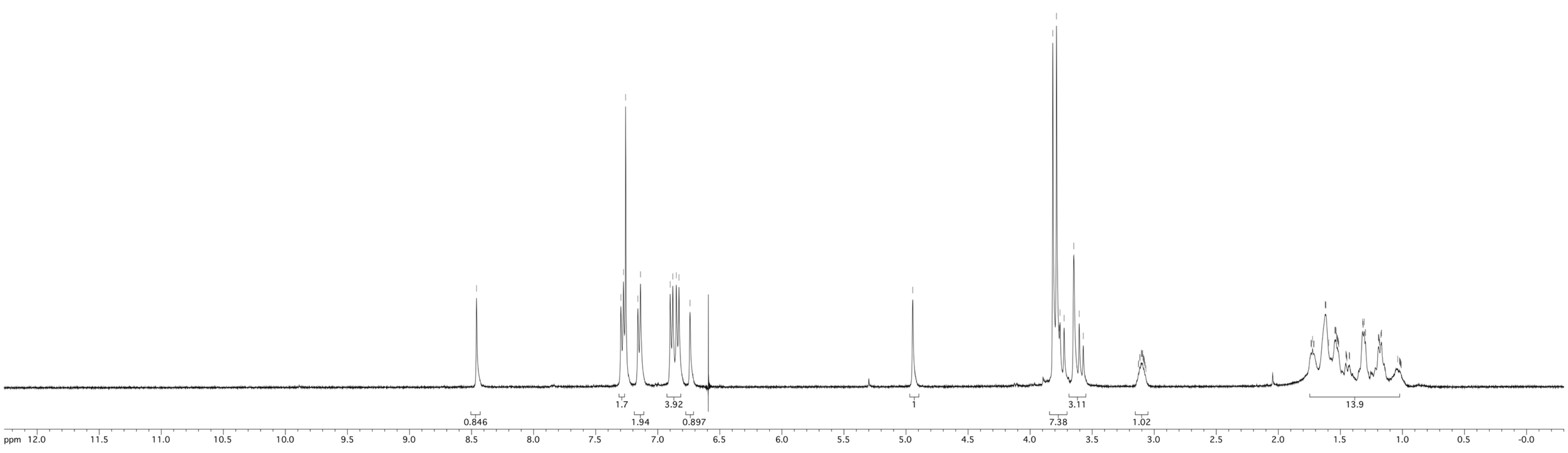

^{13}C NMR
100 MHz, $CDCl_3$

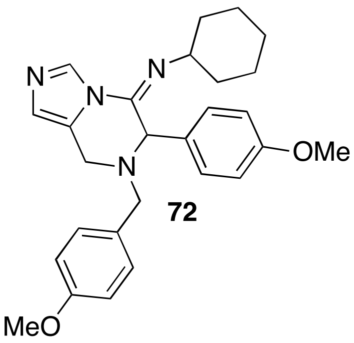

72

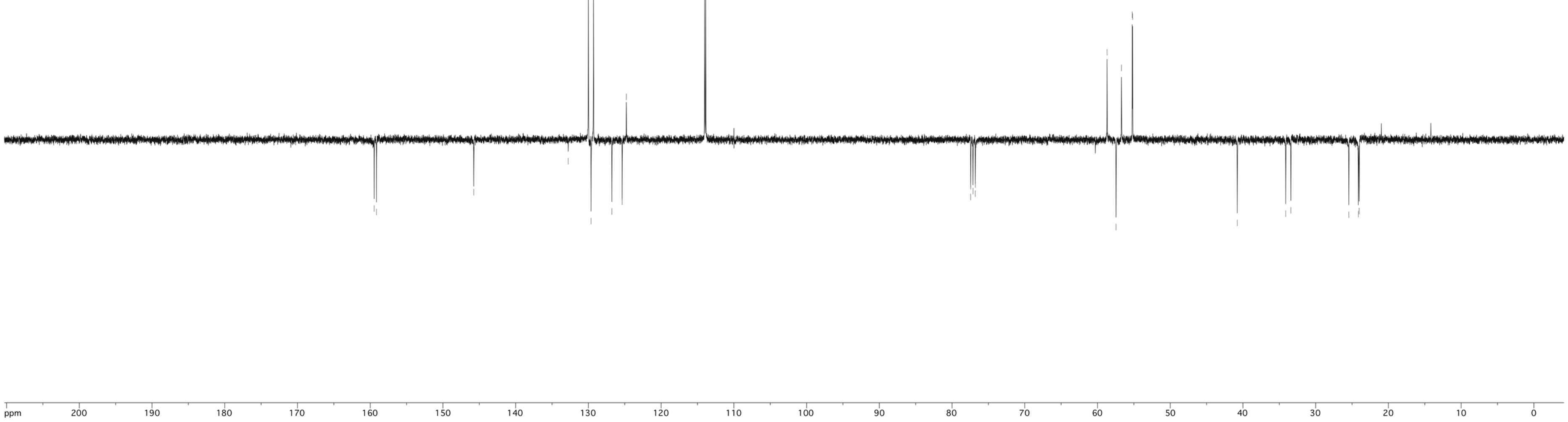

^{1}H NMR
400 MHz, $CDCl_3$

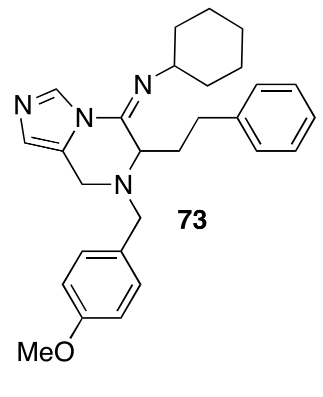

73

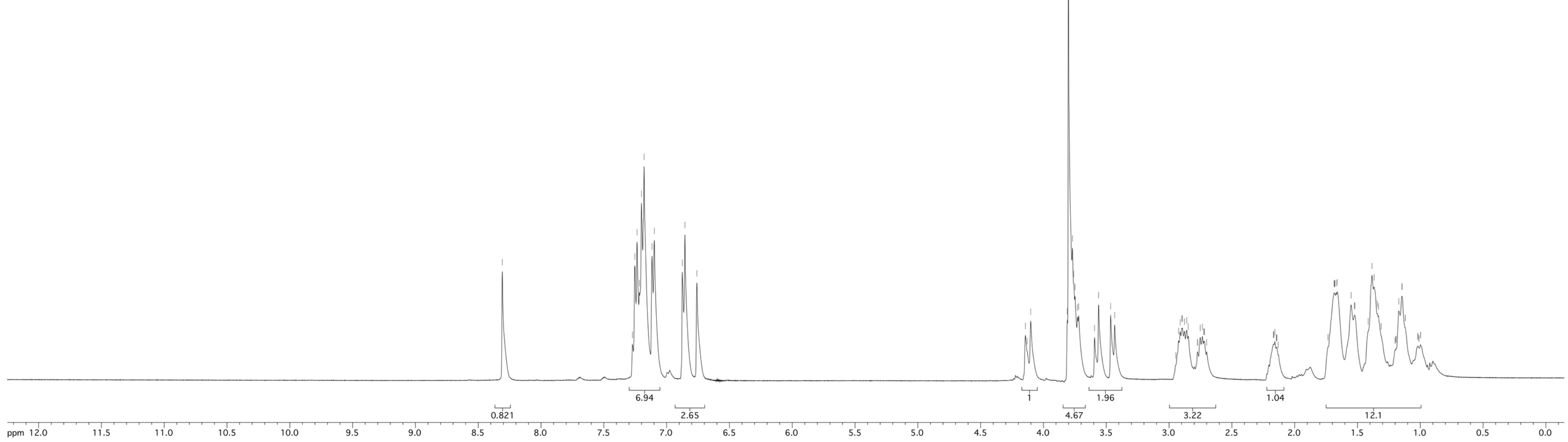

^{13}C NMR
100 MHz, $CDCl_3$

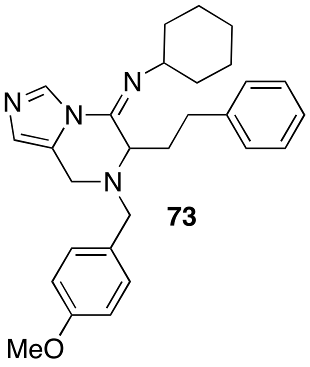

73

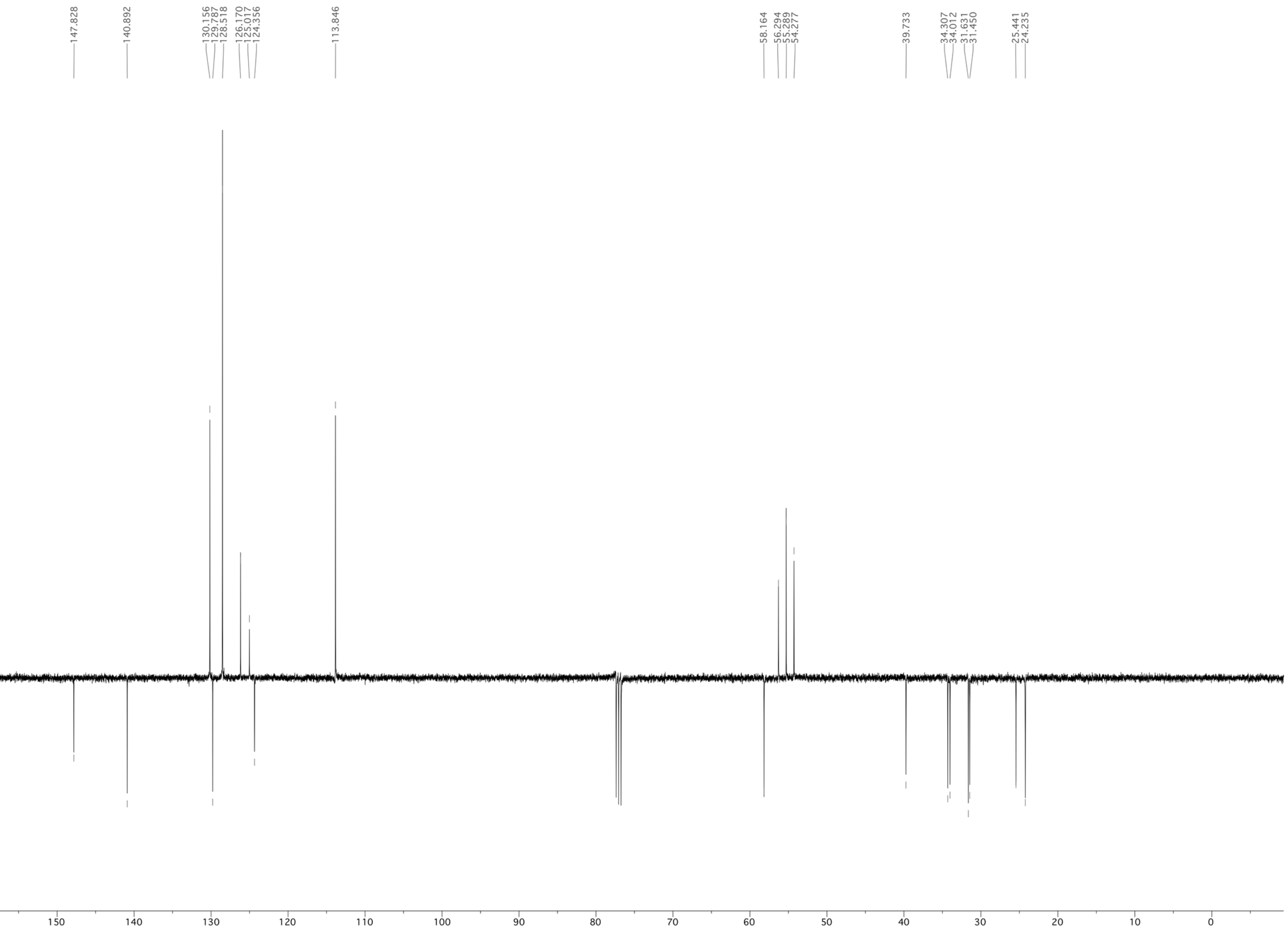

^{1}H NMR
400 MHz, $CDCl_3$

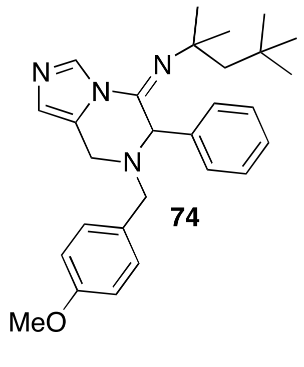

74

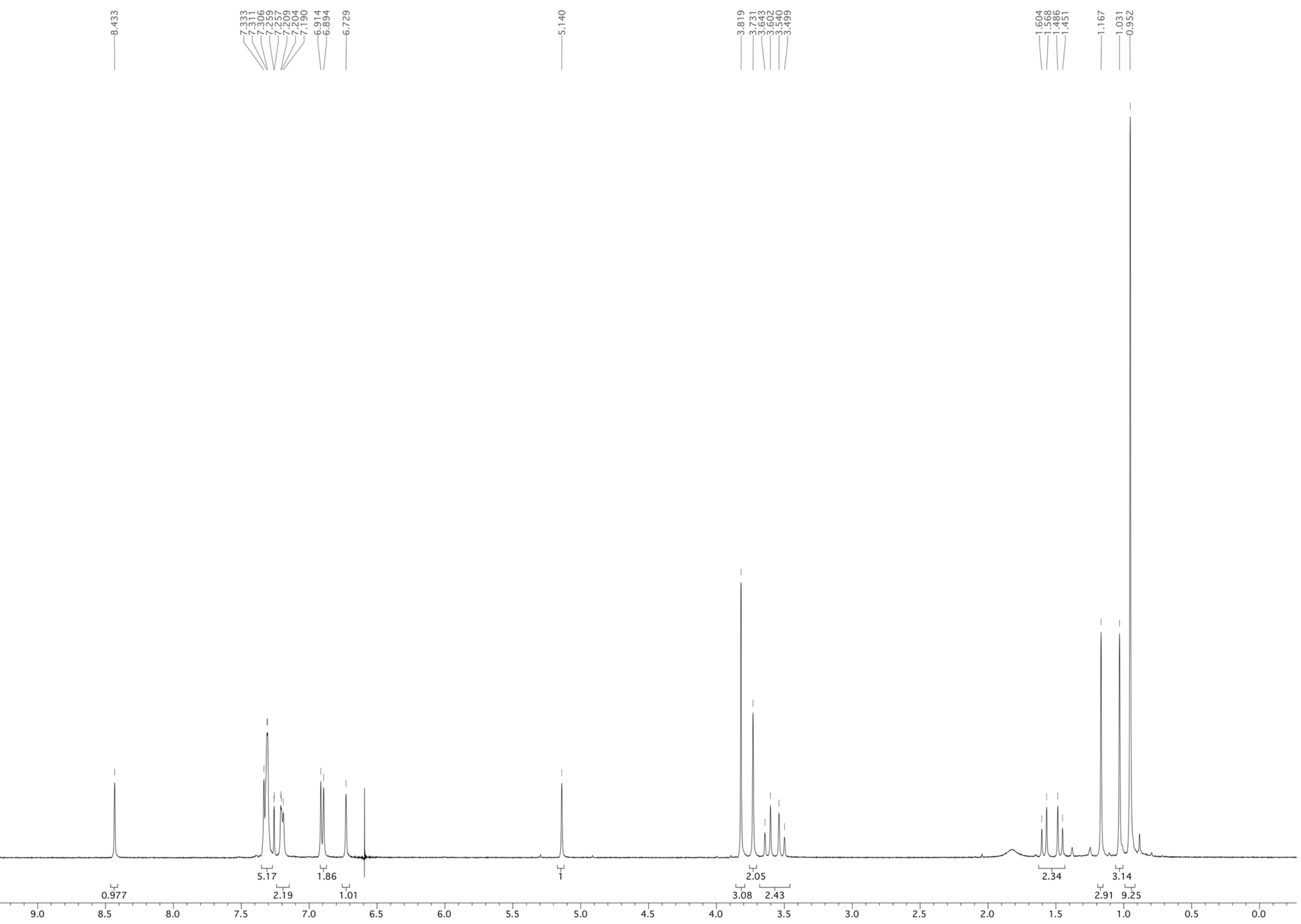

^{13}C NMR
100 MHz, $CDCl_3$

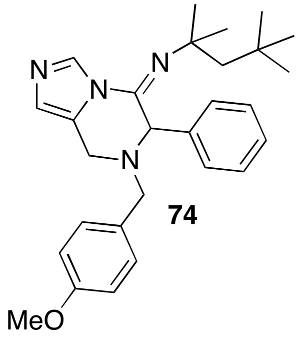

74

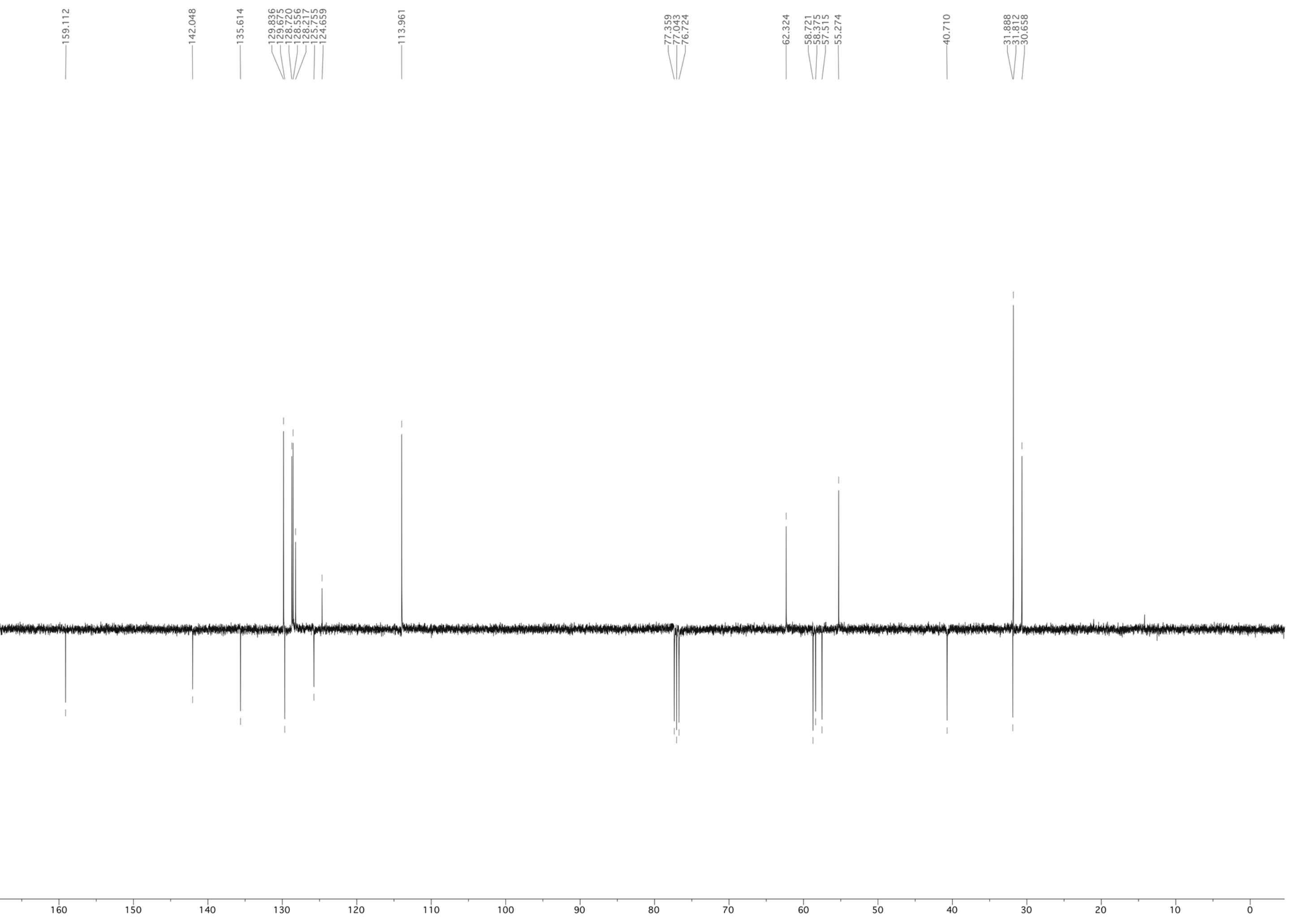

^{1}H NMR
400 MHz, $CDCl_3$

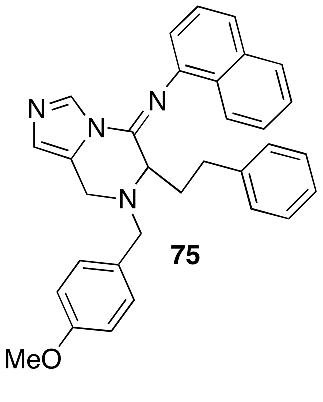

75

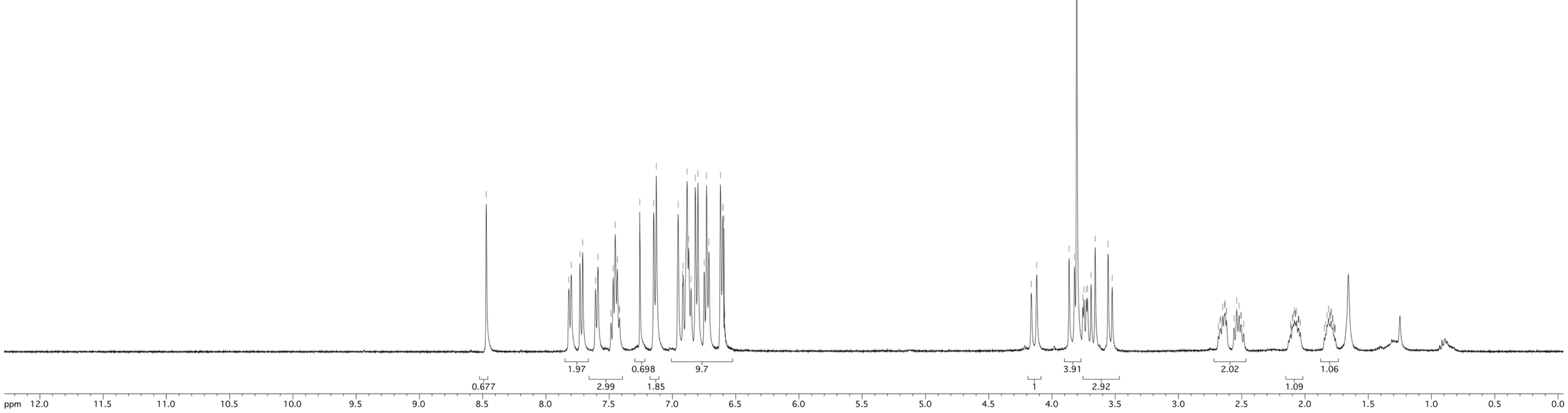

^{13}C NMR
100 MHz, $CDCl_3$

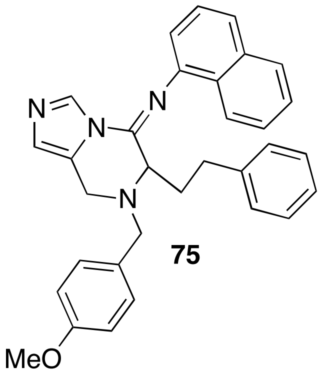

75

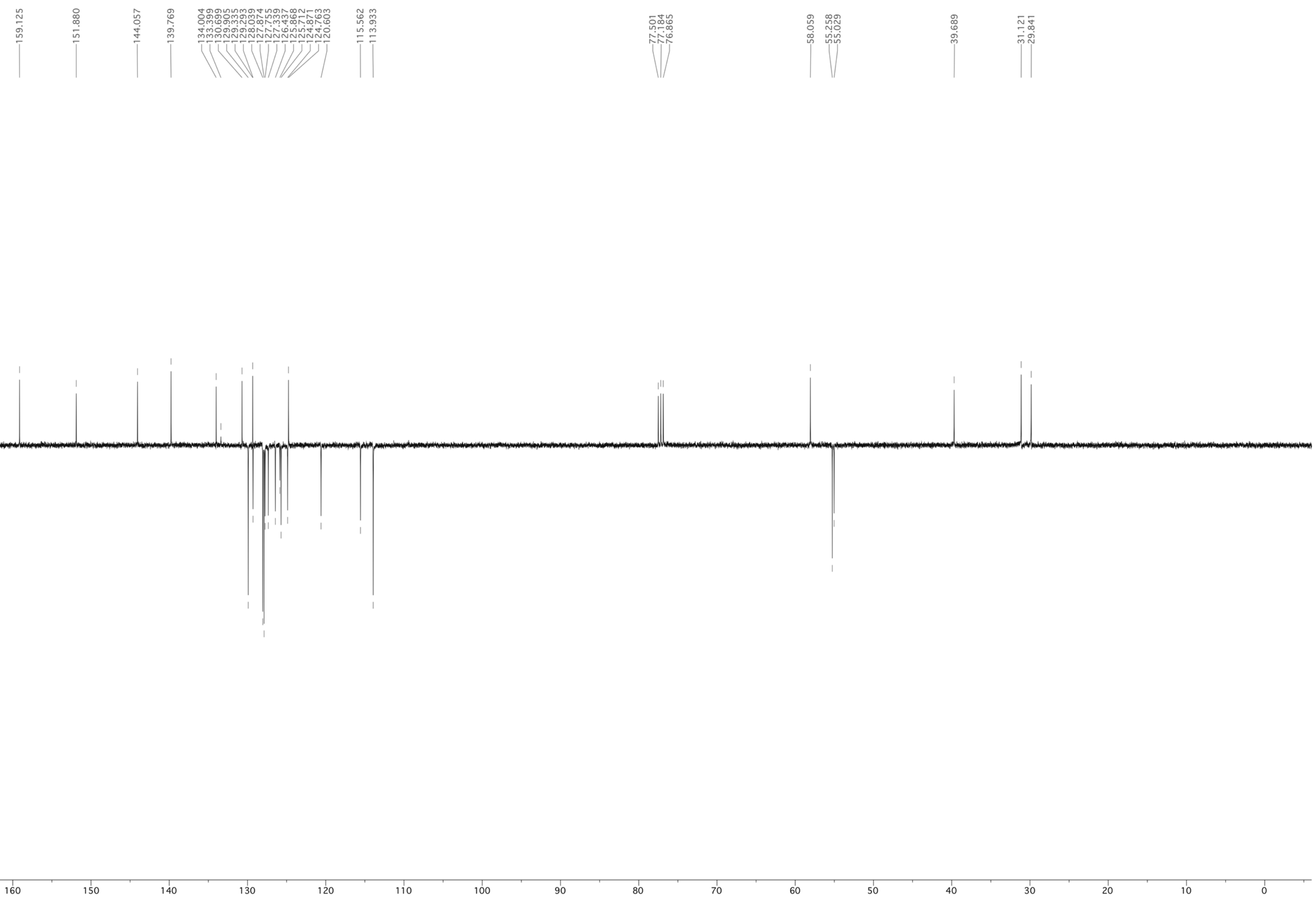

^1H NMR
400 MHz, $CDCl_3$

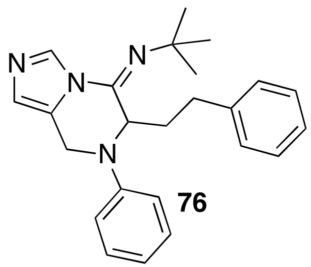

76

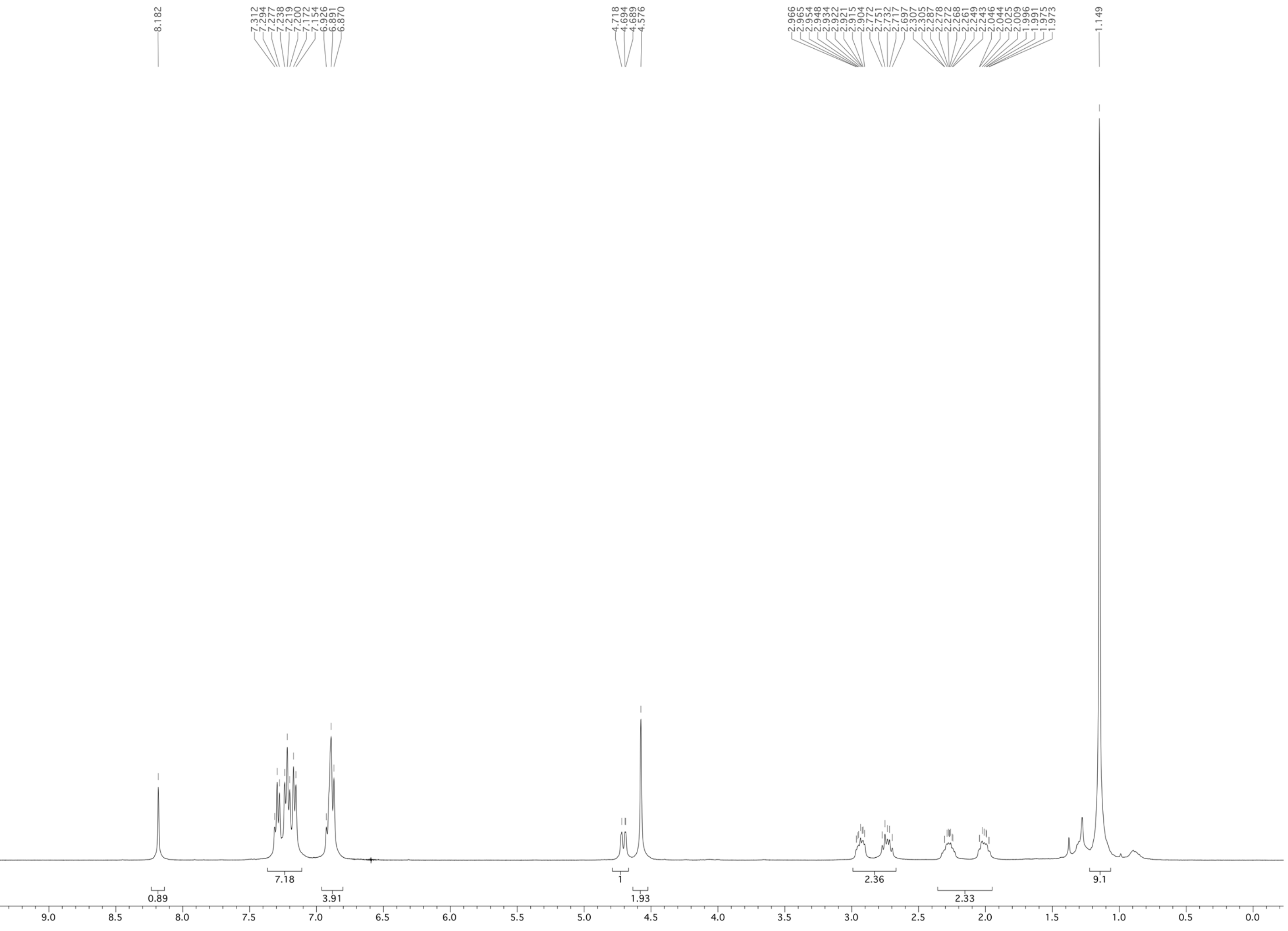

^{13}C NMR
100 MHz, $CDCl_3$

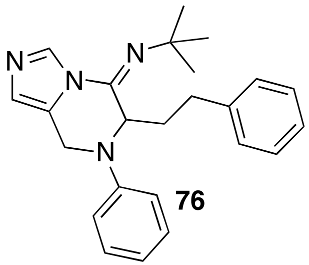

76

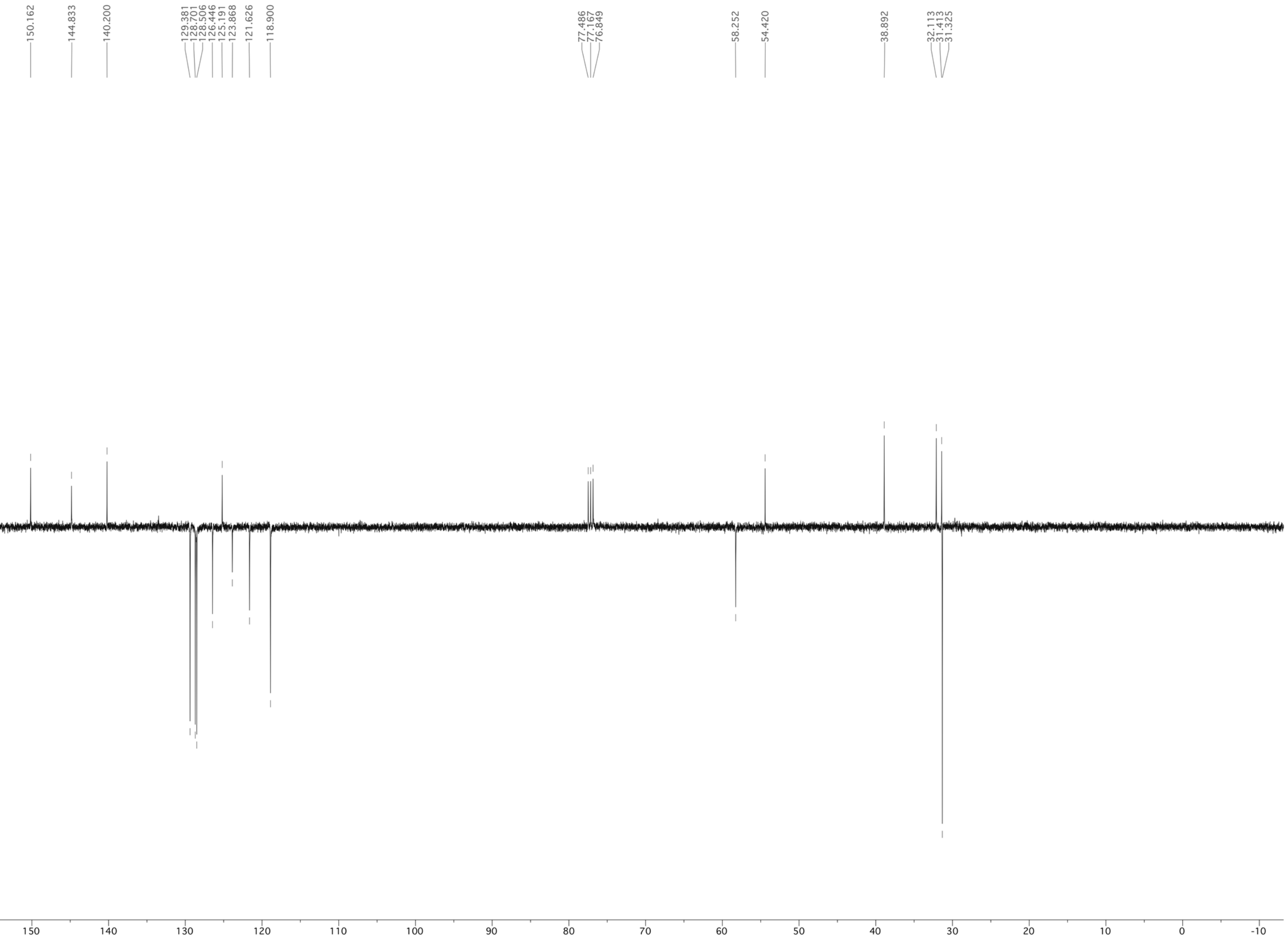

^{1}H NMR
400 MHz, $CDCl_3$

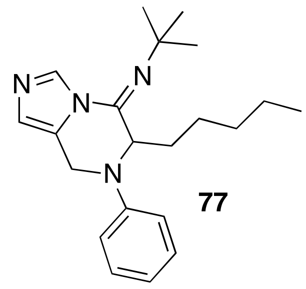

77

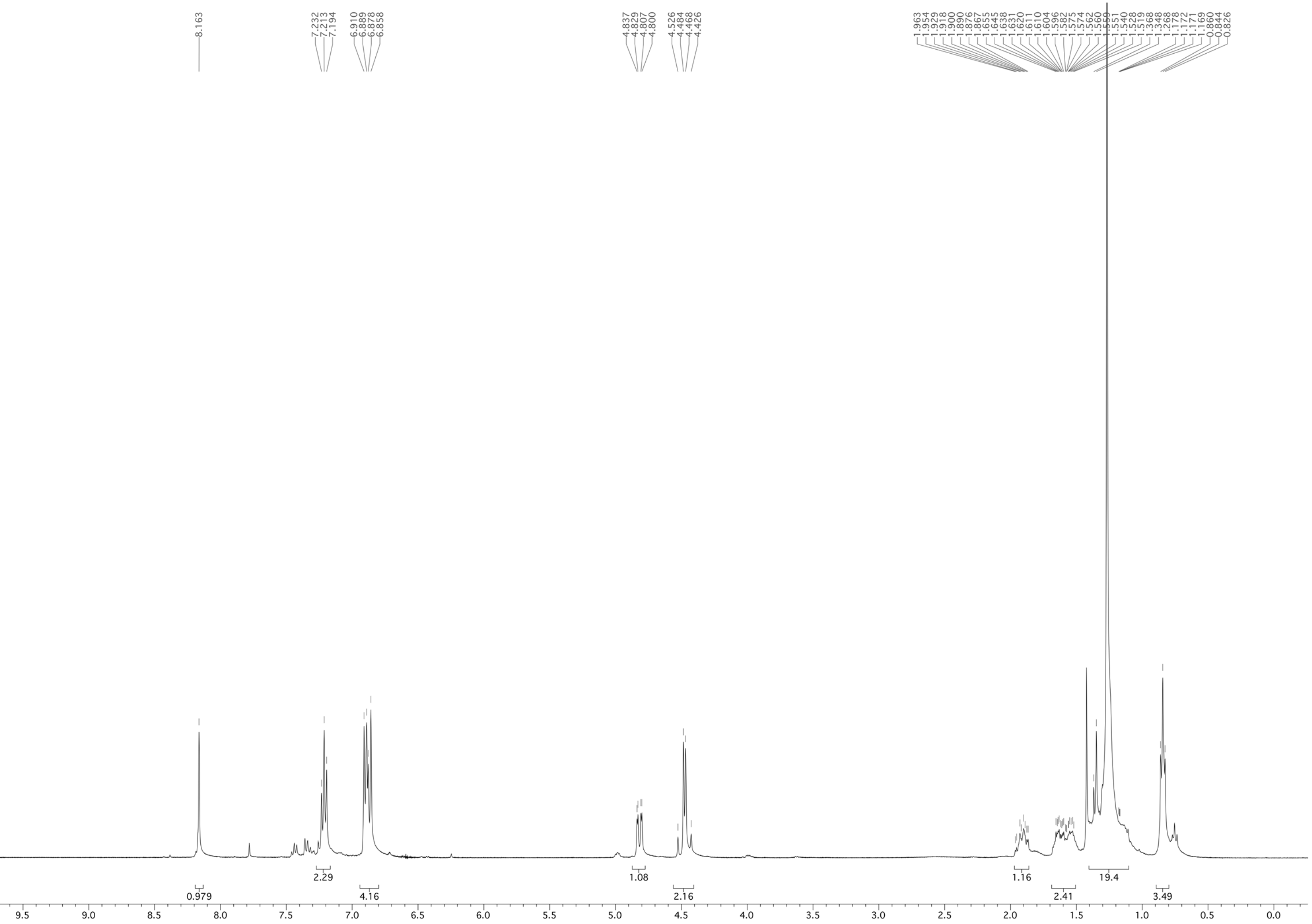

^{13}C NMR
100 MHz, $CDCl_3$

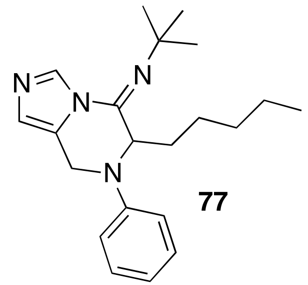

77

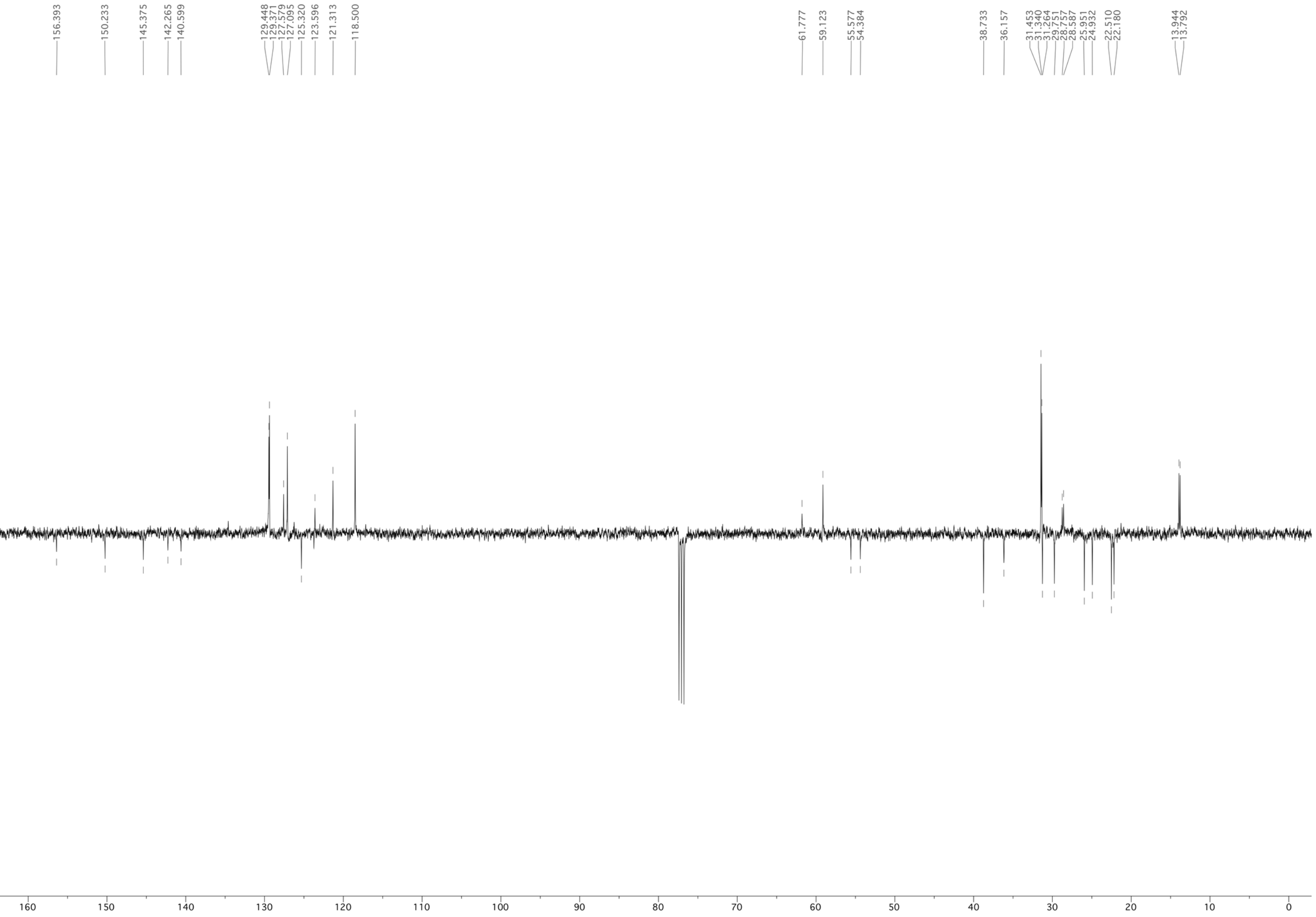

^{1}H NMR
400 MHz, $CDCl_3$

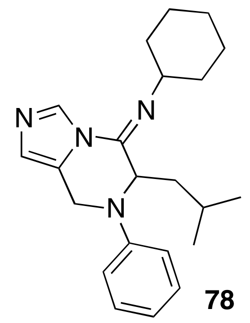

78

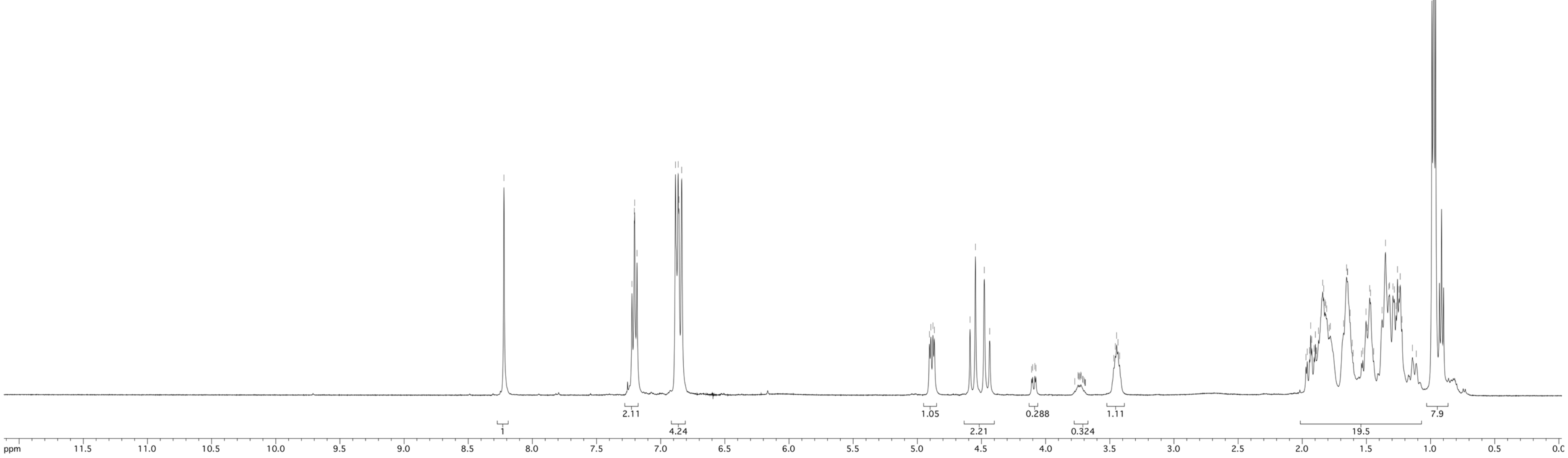

^{13}C NMR
100 MHz, $CDCl_3$

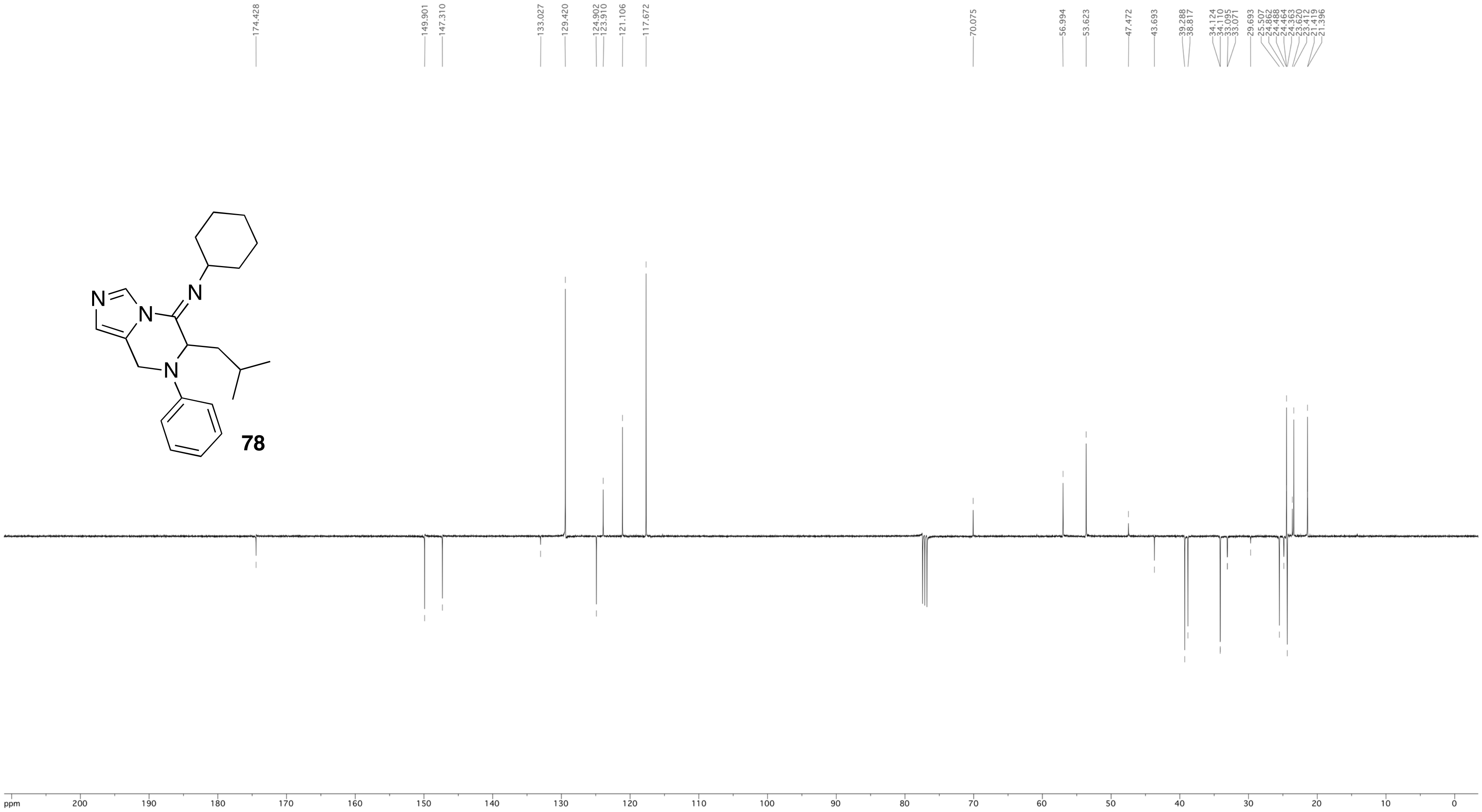

78

^1H NMR
400 MHz, $CDCl_3$

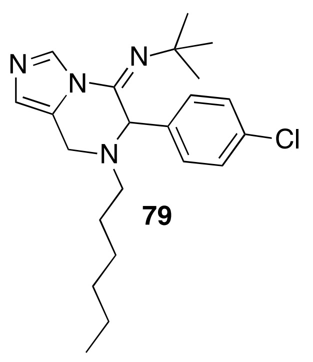

79

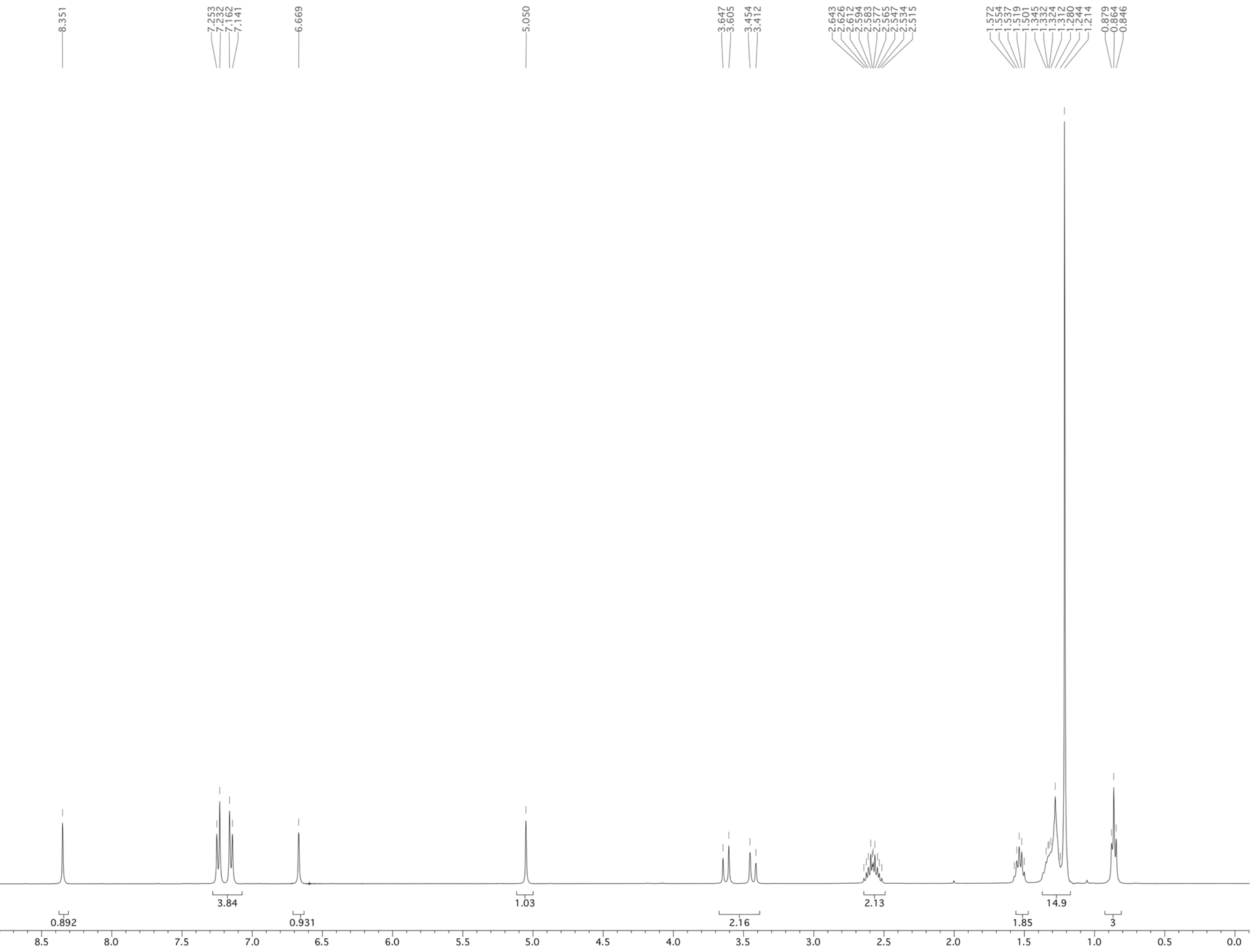

^{13}C NMR
100 MHz, $CDCl_3$

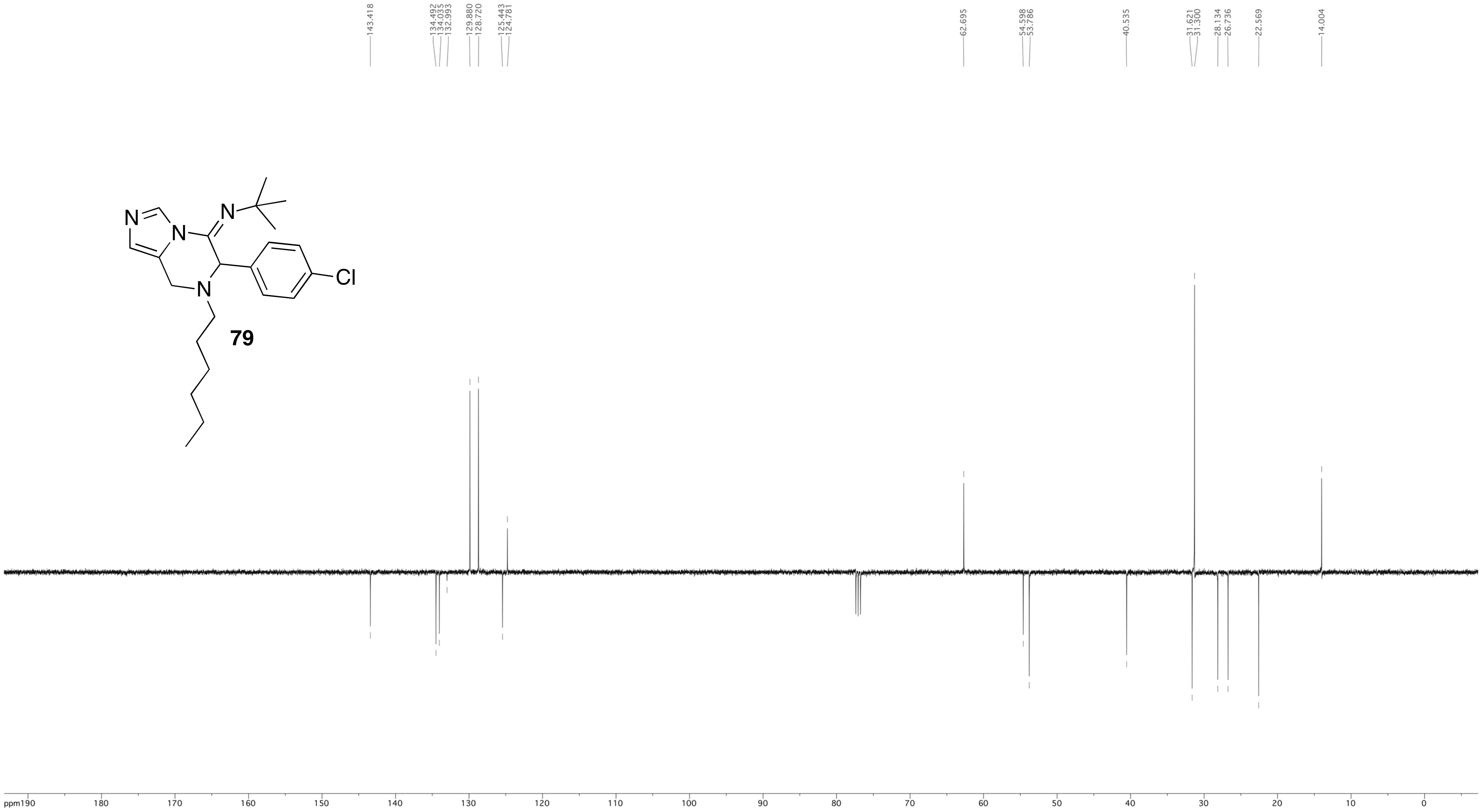

79

^{1}H NMR
400 MHz, $CDCl_3$

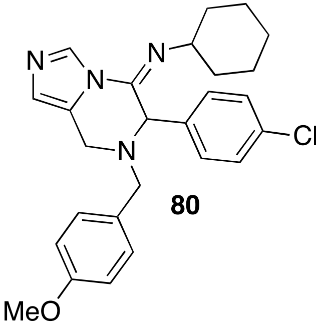

80

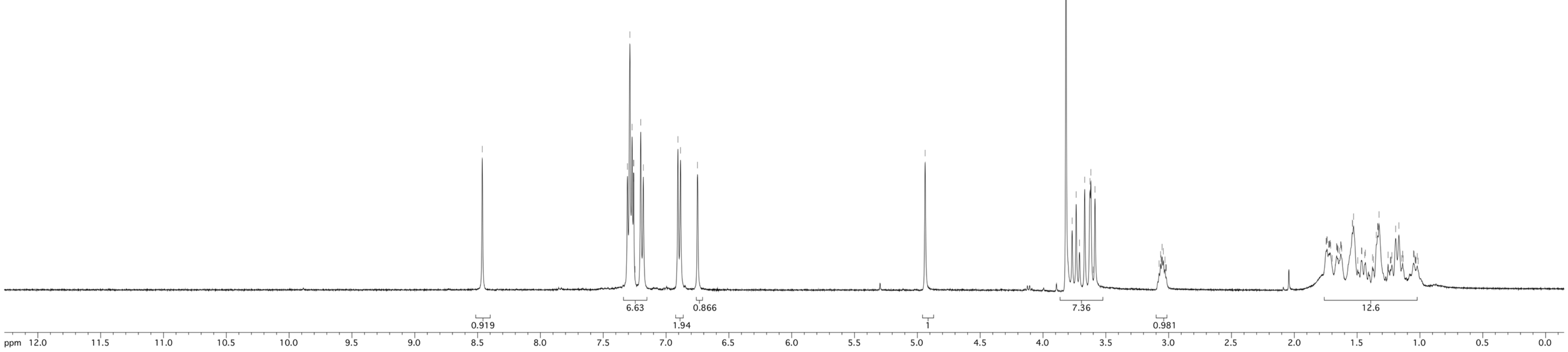

^{13}C NMR
100 MHz, $CDCl_3$

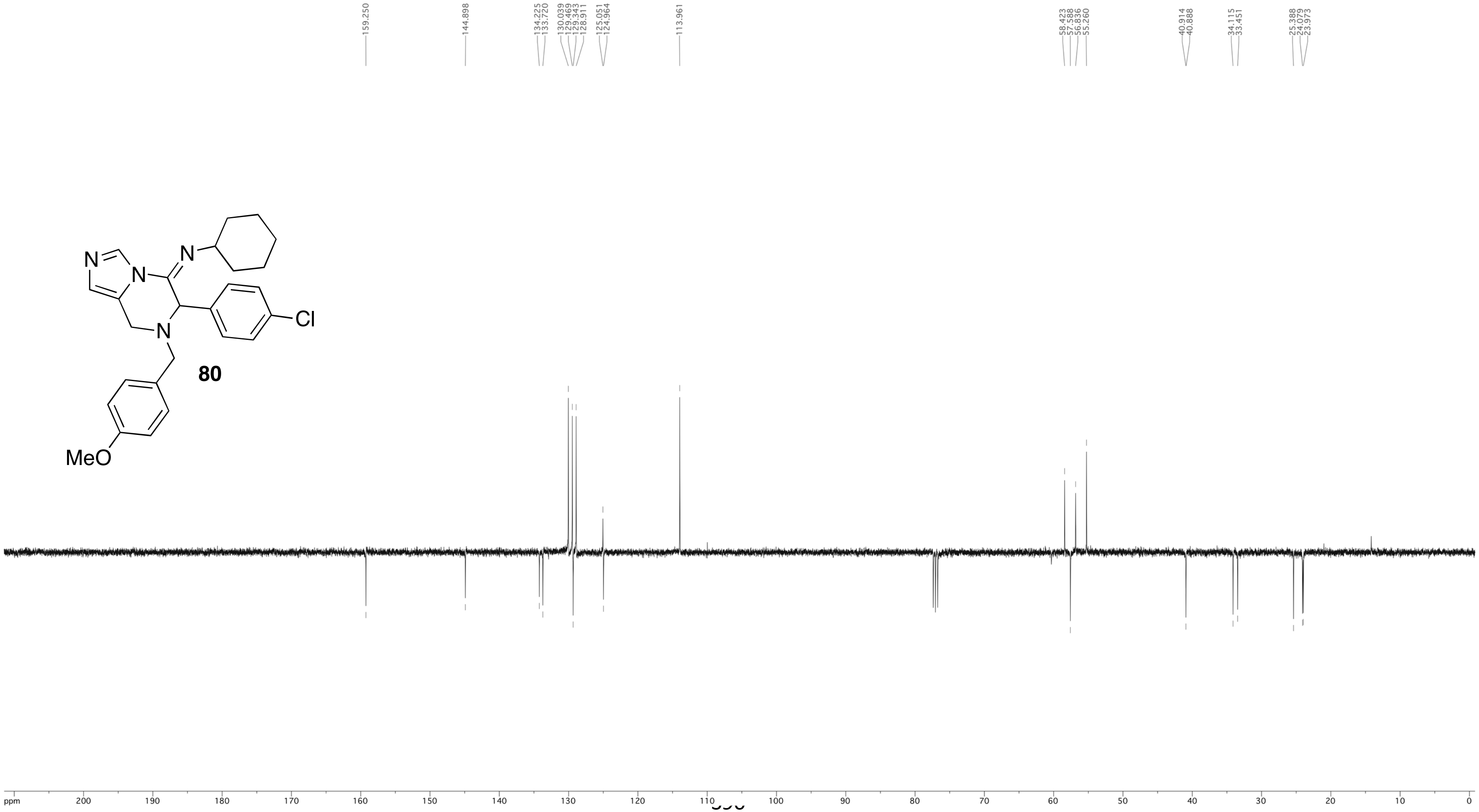

Supplement: Supplementary file 1 [file molecules-24-01959-s001.pdf]
